# Evaluation of bisphenylthiazoles as a promising class for combating multidrug-resistant fungal infections

**DOI:** 10.1371/journal.pone.0258465

**Published:** 2021-11-04

**Authors:** Mohamed Hagras, Nader S. Abutaleb, Ahmed M. Sayed, Ehab A. Salama, Mohamed N. Seleem, Abdelrahman S. Mayhoub

**Affiliations:** 1 Department of Pharmaceutical Organic Chemistry, College of Pharmacy, Al-Azhar University, Cairo, Egypt; 2 Department of Comparative Pathobiology, College of Veterinary Medicine, Purdue University, West Lafayette, Indiana, United States of America; 3 Department of Biomedical Sciences and Pathobiology, Virginia-Maryland College of Veterinary Medicine, Virginia Polytechnic Institute and State University, Blacksburg, Virginia, United States of America; 4 Department of Microbiology and Immunology, Faculty of Pharmacy, Zagazig University, Zagazig, Egypt; 5 Center for Emerging, Zoonotic and Arthropod-borne Pathogens, Virginia Polytechnic Institute and State University, Blacksburg, Virginia, United States of America; 6 Nanoscience Program, Zewail City of Science and Technology, University of Science and Technology, Giza, Egypt; UNITED STATES

## Abstract

To minimize the intrinsic toxicity of the antibacterial agent hydrazinyloxadiazole **1**, the hydrazine moiety was replaced with ethylenediamine (compound **7**). This replacement generated a potent antifungal agent with no antibacterial activity. Notably, use of a 1,2-diaminocyclohexane moiety, as a conformationally-restricted isostere for ethylenediamine, potentiated the antifungal activity in both the *cis* and *trans* forms of N-(5-(2-([1,1’-biphenyl]-4-yl)-4-methylthiazol-5-yl)-1,3,4-oxadiazol-2-yl)cyclohexane-1,2-diamine (compounds **16** and **17**). Both compounds **16** and **17** were void of any antibacterial activity; nonetheless, they showed equipotent antifungal activity *in vitro* to that of the most potent approved antifungal agent, amphotericin B. The promising antifungal effects of compounds **16** and **17** were maintained when assessed against an additional panel of 26 yeast and mold clinical isolates, including the *Candida auris* and *C*. *krusei*. Furthermore, compound **17** showed superior activity to amphotericin B *in vitro* against *Candida glabrata* and *Cryptococcus gattii*. Additionally, neither compound inhibited the normal human microbiota, and both possessed excellent safety profiles and were 16 times more tolerable than amphotericin B.

## 1. Introduction

More than 1.6 million patients die annually as a result of fungal infections. This crisis has positioned fungi at the same level of threat as tuberculosis, and the threat is 3-fold more serious than that of malaria [1]. *Candida*, *Aspergillus*, and *Cryptococcus* are the principal fungal pathogens responsible for human morbidity and mortality [2]. Clinical management of *Candida* infections is highly compromised by the increasing resistance to the current antifungal agents, such as fluconazole [3]. Further compounding the problem is the increasing incidence of infected patients with an immunodeficient status. Consequently, the mortality rate of candidemia currently ranges between 36% and 63% [3–5]. Recently, the isolation of panazole-resistant strains of *Aspergillus fumigatus* has been reported at an increasing frequency, thereby complicating disease management. Furthermore, cryptococcal meningitis is responsible for more than 180,000 deaths annually [6]. Its treatment options are limited due to the increased resistance and inadequacy of the current antifungal therapeutics [7]. Thus, the low number of available antifungals and the increasing resistance to them require the development of new classes of effective antifungals.

**Fig 1:**
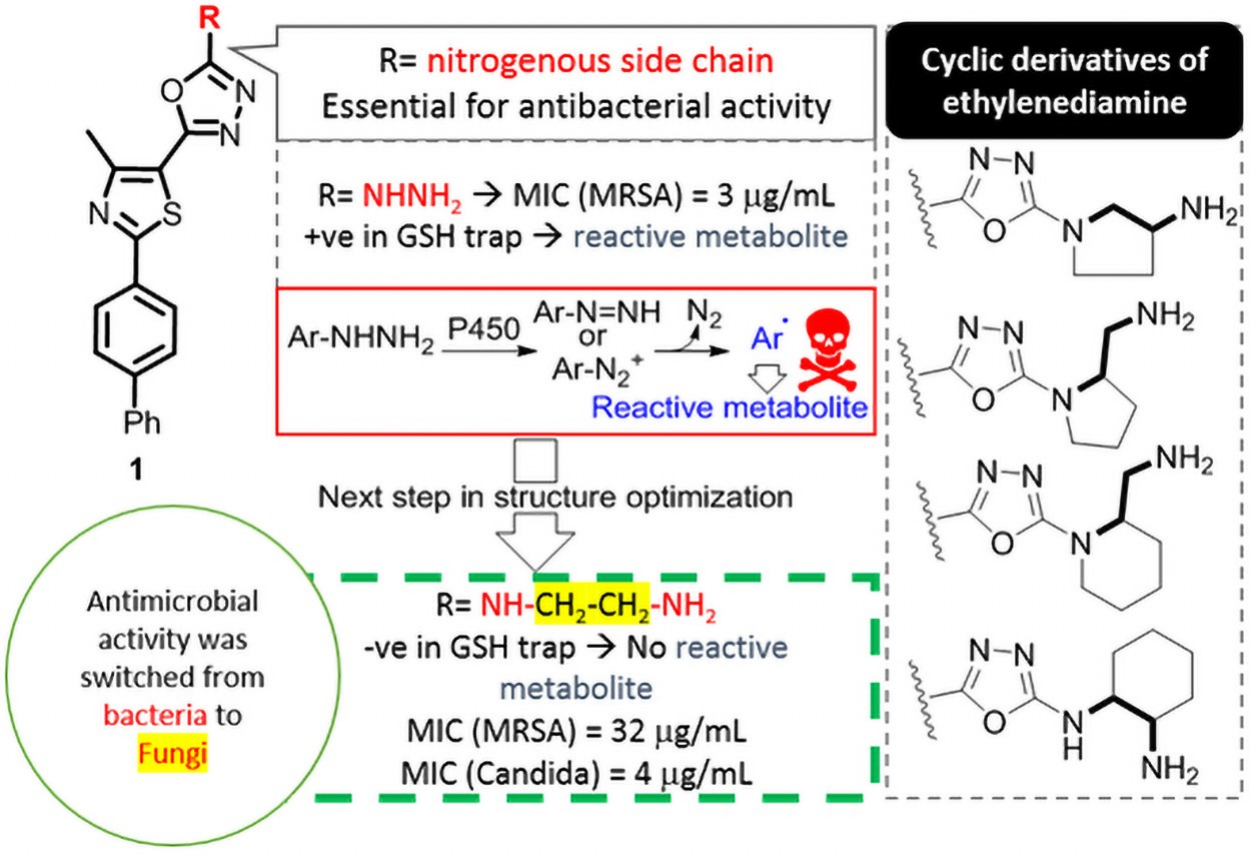
Overview of the previous observations and the newly designed structures in this study.

During the last five years, our group has optimized polysubstituted phenylthiazoles as a new class of antibacterial agents [8–10]. Our synthesized compounds were screened intuitively against bacterial and fungal species to assess their antimicrobial activity prior to further investigation. Through continuous modification, we discovered that the hydrazinyl cationic side chain provided the phenylthiazole core with potency against multidrug-resistant staphylococcal infections [11] (Fig 1). Unfortunately, activation of hydrazine inside the body is presumed to generate aryl free radicals, which results in serious oxidative stress and induces acute liver injury in the case of isoniazid and iproniazid (the two known hydrazine-containing antitubercular therapeutics with black-box warnings) [12–15]. To prevent the endogenous activation of hydrazine, we performed further derivatization by inserting methylene units between the two pharmacophoric nitrogen atoms to form ethylenediamine derivatives (1^st^ compound in Table 1). Interestingly, we found that this derivative exhibited dual antibacterial and antifungal activity (Fig 1), and we hypothesized that this mixed activity is due to the free rotation of the terminal nitrogen around the formed ethylene moiety.

**Table 1:** Initial antimicrobial screening (MICs in μg/mL) of bisphenylthiazoles.

Tested compounds/ control drugs	MRSA USA300	*C*. *difficile* ATCC BAA1870	*E*. *coli* JW55031 (*tolC*-mutant)	*E*. *coli* BW25113 (wild-type)	*C*. *albicans* SS5314 (wild-type)
**7**	32	>64	64	>64	4
**8**	32	16	16	64	16
**9**	>64	>64	>64	>64	>64
**10**	>64	32	>64	>64	8
**11**	>64	>64	>64	>64	>64
**12**	16	32	>64	>64	16
**13**	>64	>64	>64	>64	>64
**14**	>64	64	>64	>64	>64
**15**	16	16	16	>64	16
**16**	64	32	64	>64	1
**17**	>64	64	>64	>64	1
**18**	32	8	32	>64	16
**19**	>64	64	>64	>64	>64
**20**	>64	64	>64	>64	>64
**21**	>64	>64	>64	>64	>64
Linezolid	1	1	8	>64	NT^1^
Vancomycin	1	NT	NT	NT	NT
Gentamicin	NT	NT	≤0.5	≤0.5	NT
Fluconazole	NT	NT	NT	NT	1
Amphotericin B	NT	NT	NT	NT	1

NT^1^: not tested.

In the present work, we aimed to maximize the antifungal potency of bisphenylthiazoles by studying the structure-activity relationships (SAR) at oxadiazole position-2. In this regard, several bio-isosteres to the ethylenediamine moiety were utilized with a specific focus on cyclic alternatives and the stereochemistry of the two amines.

## 2. Results and discussion

### 2.1. Chemistry

Methylsulfone, as a good leaving group, was used to access nucleophilic aromatic substitution on the oxadiazole ring. Compound **6** was prepared as previously reported [11], starting from the acid hydrazine (compound **4**), and then the compound allowed to react with various amine-containing synthons to yield the desired final products, compounds **7–21** (Scheme 1).

**Scheme 1**. Reagents and conditions: (a) Absolute EtOH, ethyl 2-chloroacetoacetate, heat at reflux, 4 h, (b) Absolute EtOH, NH_2_NH_2_ H_2_O, heat at reflux, 8 h; (c) CS_2_, KOH, EtOH, heat at reflux, 12 h; (d) dimethyl sulfate, KOH, H_2_O, stirring at 23 °C, 2 h; (e) MCPBA, dry DCM, 23 °C, 16 h; (f) appropriate amine; K_2_CO_3_, DMF, heat at 80 °C for 0.5–12 h.

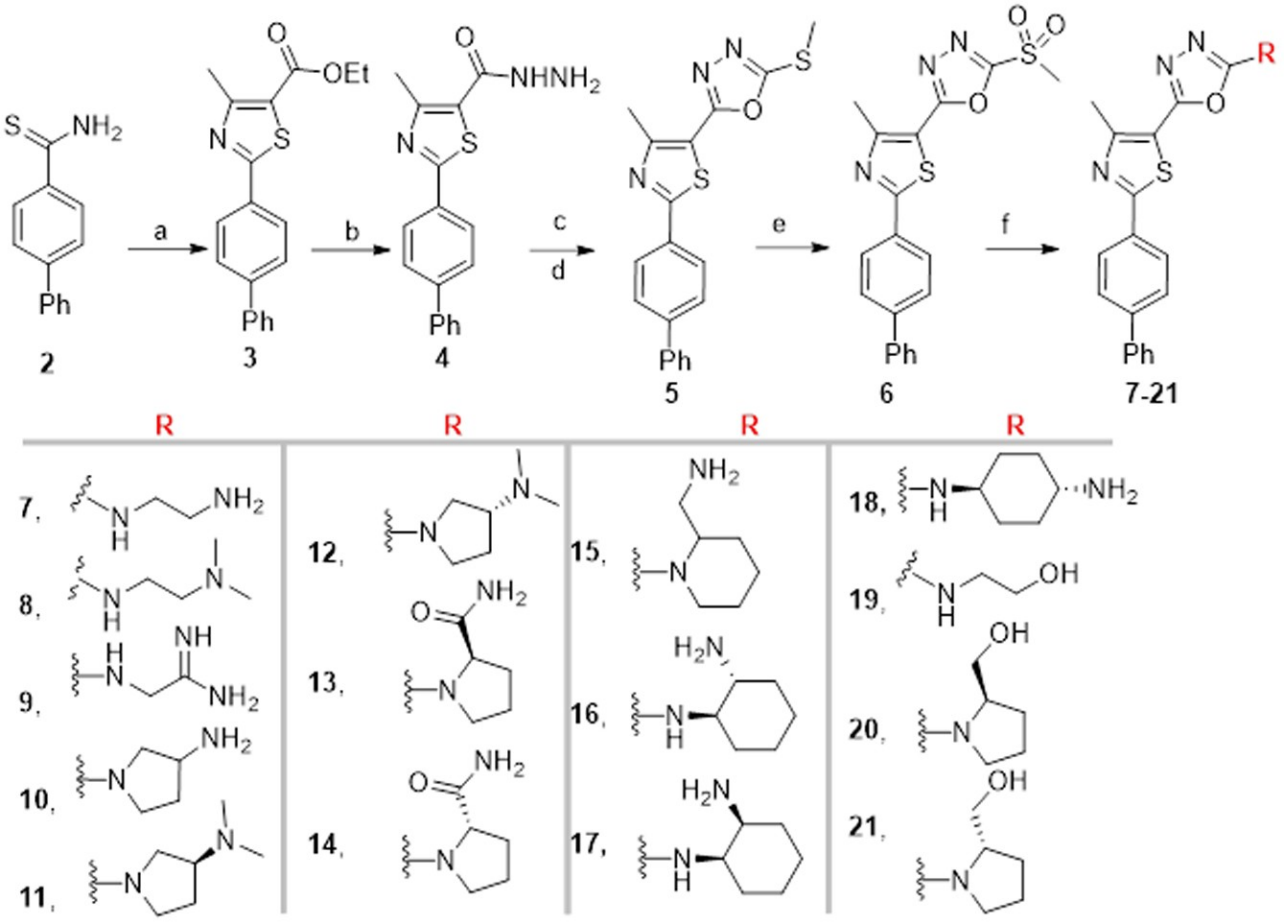


The stereochemistry of the final products was derived from their corresponding starting materials because a one-step substitution reaction was not expected to affect the spatial configuration of the final compounds. Of note, the chemical structures of compounds **10** and **15** were found to be joined by an oxadiazole ring via the pyrrolidine/piperidine nitrogen rather than the terminal primary amine-based on their spectral data. In brief, all diaminocyclohexane-containing compounds (**16–18**) displayed two broad deuterium-exchangeable signals, with one within the aromatic region between 6.7 and 7.9 ppm, which was equivalent to one proton. However, a signal that was equivalent to two protons was displayed in the upper field region at approximately δ 1.6. Therefore, the presence of only one broad signal, which was equivalent to two deuterium-exchangeable protons, in the cases of compounds **10** and **15**, indicates that the pyrrolidine and piperidine NH groups replaced the methylsulfonyl moiety of compound **6**, which confirms the proposed chemical structures of the final products (**10** and **15**).

### 2.2. Biological results and discussion

#### 2.2.1. Initial antimicrobial SAR

Our synthesized compounds were tested against two Gram-positive bacteria (MRSA and *Clostridioides difficile*), two Gram-negative bacterial strains (*Escherichia coli tolC*-mutant and wild-type), and a fungal species (*Candida albicans*). The ethylenediamine-containing derivative **7** showed moderate antifungal activity against *C*. *albicans* (MIC = 4 μg/mL) and weak antibacterial activity against the tested bacterial strains.

Because we anticipate that the high flexibility of the free-rotating ethylenediamine motif is responsible for the dual antibacterial/antifungal activities of compound **7**, we hypothesized that the antifungal effect would be enhanced when the spatial arrangement matched the bioactive form. Consequently, we synthesized a small set of conformationally-restricted analogs. In this regard, the cyclohexyl moiety with 1,2-diamine provided the most potent derivatives (compounds **16** and **17**). Both compounds were equipotent to amphotericin B against the tested strain (MIC = 1 μg/mL). The 1,4-diaminocyclohexane derivative (compound **18**) was prepared with the objective of testing the effect of increasing the carbon chain length between the two amines on the antimycotic activity. The MIC results revealed that increasing the number of carbons between the two amines had a negative impact on the antifungal activity, suggesting that a distance of two carbon units is optimal for the activity. However, all pyrrolidine-containing derivatives (**10–14**) were less active than the lead compound (**7)** (Table 1).

Next, we determined whether the hydroxyl group can act as a bio-isostere to the terminal amine. The high MIC values of the hydroxylated derivatives (**19–21**) against all tested microbes confirmed the importance of the terminal primary amine for the antimicrobial activity. Dimethylation (compound **8**) or replacement with an imidine group (compound **9**) significantly reduced the antimycotic activity.

Next, we were interested to investigate the anticommensal activity of our prioritized compounds (**7**, **16** and **17**). The human body contains a large number of beneficial microorganisms, which play a significant role in combating pathogens, provide essential elements to the host cells and facilitate the functionality of the host immune system [16]. The ideal antifungal therapeutic should not affect these important roles of the normal microbiota. Therefore, our most promising compounds **7**, **16** and **17** were tested against different normal human microbiota strains including *Lactobacillus gasseri*, *L*. *casei*, and *L*. *crispatus*. The compounds did not inhibit the tested normal human microbiota strains (Table 2).

**Table 2:** Activity of bisphenylthiazoles against the normal human microbiota (*Lactobacillus* spp).

Bacterial strains	Compounds/ Control antifungals (MICs in μg/mL)				
	7	16	17	Fluconazole	Amphotericin B
***Lactobacillus gasseri* HM-400**	64	>128	128	>128	>128
***Lactobacillus casei* ATCC-334**	>128	>128	128	>128	>128
***Lactobacillus crispatus* HM-370**	>128	>128	>128	>128	>128

*2*.*2*.*1*.*1*. *Profiling of antifungal activity*. We next moved to expand the antifungal assessment using a large panel of clinically important fungal strains. In general, compounds **7**, **16** and **17** exhibited a broad spectrum of activity against all the tested *Candida* and *Cryptococcus* isolates.

Compounds **16** and **17** exhibited potent activity against *C*. *albicans*, inhibiting the growth of tested strains at concentrations ranging from 2 to 4 μg/mL (Table 3). Additionally, these compounds maintained the same activity against the fluconazole-resistant *C*. *albicans* strain NR-29448. New compounds, capable of exhibiting potent activity against fluconazole-resistant *C*. *albicans* strains, are highly desirable because overcoming *C*. *albicans* infections is more difficult due to the increasing resistance to azoles, specifically fluconazole [17].

**Table 3:** Antifungal activity of bisphenylthiazoles against *Candida* and *Cryptococcus* clinical isolates.

Fungal strains	Compounds/Control antifungals MICs (μg/mL)					
	Fluconazole	Amphotericin B	Caspofungin	7	16	17
***C*. *albicans* ATCC 10231**	1	1	0.125	8	4	4
***C*. *albicans* NR-29448**	>128	1	0.25	16	2	2
***C*. *glabrata* ATCC 66032**	8	2	0.25	2	1	0.5
***C*. *glabrata* ATCC MYA-2950**	8	1	0.125	1	1	0.5
***C*. *parapsilosis* ATCC 22019**	1	0.5	0.5	2	1	1
***C*. *parapsilosis* CAB 502638**	0.5	0.5	0.5	2	1	1
***C*. *tropicalis* ATCC 1369**	0.5	1	0.125	8	4	4
***C*. *krusei* CAB 396420**	16	2	0.5	2	1	1
***C*. *krusei* ATCC 34135**	16	2	0.5	2	1	1
***C*. *neoformans* NR 41298**	2	1	NT^1^	2	1	1
***C*. *neoformans* NR 41300**	1	1	NT	4	1	1
***C*. *neoformans* NR 48770**	2	0.5	NT	2	1	1
***C*. *gattii* NR 43210**	4	0.5	NT	2	1	0.5
***C*. *gattii* NR 43209**	4	1	NT	2	1	0.5

NT^1^, not tested.

Moreover, the bisphenylthiazole activity was extended to include other non-albicans *Candida* (NAC) species such as *C*. *glabrata*, *C*. *parapsilosis*, *C*. *tropicalis* and *C*. *krusei* with MICs ranging from 0.5 to 4 μg/ mL (Table 3). The MICs of compounds **16** and **17** were 2–4 folds lower than that of amphotericin B and 8 to 16 folds lower than that of fluconazole, when tested against two *C*. *glabrata* strains. Caspofungin inhibited the tested *C*. *glabrata* strains at concentrations ranging from 0.125 μg/mL to 0.25 μg/mL. *C*. *glabrata* is the most common NAC and is isolated from vulvovaginal candidiasis and candiduria [18]. As a causative agent of superficial or systemic candidal infections, *C*. *glabrata* is ranked second or third according to the geographical distribution [19, 20]. In the United States, Europe, and Australia, *C*. *glabrata* was reported to be the second most common cause of vulvovaginal candidiasis after *C*. *albicans* [21]. Additionally, it has been reported that approximately 3% of *C*. *glabrata* isolates are resistant to echinocandins, the first-line treatment for this pathogen, and have high levels of resistance to azoles, that further complicates the treatment of *C*. *glabrata* infections [22].

Additionally, *C*. *krusei* was susceptible to both diaminocyclohexane derivatives (compounds **16** and **17)**; these compounds were one-fold more potent than the drug of choice (amphotericin B) in such cases and several times more potent than the most commonly prescribed antifungal drug (fluconazole). Currently, *C*. *krusei* is common in patients with hematological malignancies and causes invasive candidiasis. In addition, approximately 2.7% of NAC isolated cases belong to this strain [23, 24]. Most *C*. *krusei* isolates are intrinsically resistant to azoles [24]. Furthermore, compounds **16** and **17** exhibited potent activity against other tested NAC species such as *C*. *parapsilosis* and *C*. *tropicalis*, with MICs ranging from 1 to 4 μg/mL.

The newly synthesized compounds showed promise in controlling cryptococcal infections, which are responsible for hundreds of thousands of deaths annually [6]. Our most potent compounds inhibited the tested strains at concentrations as low as 0.5–1 μg/mL. They were equipotent to the drug of choice, amphotericin B.

Among all *Candida* infections, *C*. *auris* is of particular clinical concern because this particular species has emerged as a highly resistant fungal pathogen that has been isolated on five continents [25]. Despite implementing strict infection prevention and control measures, *C*. *auris* is associated with numerous nosocomial outbreaks worldwide [26, 27]. Additionally, *C*. *auris* showed an enhanced resistance profile to most antifungal agents, especially to first-line treatment with oral fluconazole, making *C*. *auris* infections one of the most difficult fungal infections to treat. Up to 93% of *C*. *auris* isolates have been reported to be fluconazole-resistant [28]. Unfortunately, fluconazole treatment failure has been reported for fluconazole-sensitive isolates in the USA [29]. Additionally, reduced susceptibility to other antifungals such as voriconazole, itraconazole, and isavuconazole, has been reported [30–32]. Moreover, some isolates were reported to be amphotericin B-resistant. Hence, the great need for novel broad-spectrum antifungal compounds cannot be overemphasized. Therefore, we screened our most promising compounds against ten *C*. *auris* strains. Interestingly, compounds **16** and **17** displayed potent activity against all *C*. *auris* strains, including fluconazole-resistant strains, inhibiting their growth, with MIC values ranging from 1 to 2 μg/mL (Table 4). The antifungal activity of both bisphenylthiazole compounds against *C*. *auris* strains was similar to that of the standard antifungal agent amphotericin B. Conversely, the lead compound **7** exhibited moderate activity against all tested *C*. *auris* strains with MIC values ranging from 2 to 8 μg/mL.

**Table 4:** The activity of bisphenylthiazoles against a panel of *Candida auris* clinical isolates.

Fungal strains	Compounds/Control antifungals MICs (μg/mL)				
	Fluconazole	Amphotericin B	7	16	17
***C*. *auris* 381**	1	1	8	1	1
***C*. *auris* 382**	8	1	8	2	2
***C*. *auris* 383**	>128	1	2	1	1
***C*. *auris* 384**	>128	1	4	1	1
***C*. *auris* 385**	>128	1	4	2	2
***C*. *auris* 386**	>128	2	8	1	1
***C*. *auris* 387**	8	1	8	2	2
***C*. *auris* 388**	>128	2	4	1	1
***C*. *auris* 389**	>128	2	8	2	2
***C*. *auris* 390**	>128	2	2	1	1

Next, we investigated whether the activity of bisphenylthiazoles could be expanded against molds. Compounds **16** and **17** exhibited reasonable activity against *Aspergillus fumigatus*, with MIC values of 4 μg/mL, which exceeded the activity of the lead compound (**7)** (Table 5). Due to the inadequacy of the current antifungals and the increased resistance of *Aspergillus* to the available drugs, the mortality rate of aspergillosis exceeds 88% [33].

**Table 5:** The activity of bisphenylthiazoles against *Aspergillus fumigatus* clinical isolates.

*Aspergillus* strains	Compounds/Control antifungals MICs (μg/mL)			
	Itraconazole	7	16	17
***A. fumigatus* NR 35303**	1	16	4	4
***A. fumigatus* NR 35304**	1	8	4	4

#### 2.2.2. The fungistatic activity of compound 17

After confirming the broad spectrum of activity of bisphenylthiazoles against fungal strains, we explored their mode of inducing cell death. The minimum fungicidal concentration of the most potent compound (compound **17**) was determined against several *Candida* strains including *C*. *albicans*, *C*. *auris*, *C*. *glabrata*, *C*. *tropicalis* and *C*. *krusei*. The compound showed fungistatic activity against the strains tested (Table 6). Fluconazole exhibited a fungistatic activity while amphotericin B exhibited a fungicidal effect with MFC values ranging from 1 to 2 μg/mL.

**Table 6:** The minimum inhibitory concentrations (MICs, in μg/mL) and minimum fungicidal concentrations (MFCs, in μg/mL) of compound 17 and the control drugs against *Candida auris* clinical isolates.

Tested strains	Compounds/Control antifungals					
	17		Amphotericin B		Fluconazole	
	MIC	MFC	MIC	MFC	MIC	MFC
***C*. *albicans* ATCC 10231**	4	>64	1	1	1	>128
***C*. *albicans* NR-29448**	2	>64	1	2	>128	>128
***Candida auris* 389**	2	>64	2	2	>128	>128
***Candida auris* 390**	1	>64	1	2	>128	>128
***C*. *glabrata* ATCC 66032**	0.5	>64	2	2	8	>128
***C*. *glabrata* ATCC MYA-2950**	0.5	>64	1	1	8	>128
***C*. *tropicalis* ATCC 1369**	4	>64	1	2	0.5	>128
***C*. *krusei* CAB 396420**	1	>64	2	2	16	>128

#### 2.2.3. Toxicity profile for bisphenylthiazole compounds

**Fig 2:**
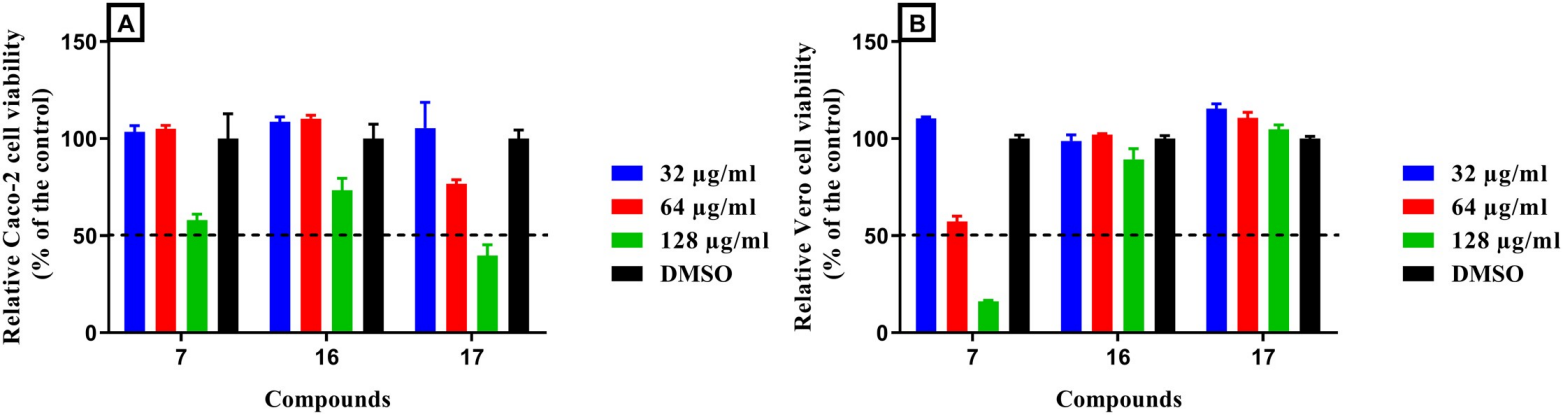
*In vitro* cytotoxicity assay of bisphenylthiazole compounds 7, 16 and 17 (tested in triplicates at 32, 64 and 128 μg/mL) against: A) human colorectal cells (Caco-2), and B) monkey kidney epithelial cells (Vero) after 24-hours exposure time, using the MTS 3-(4,5-dimethylthiazol-2-yl)-5-(3-carboxymethoxyphenyl)-2-(4-sulfophenyl)-2*H*-tetrazolium) assay. The data represent the percentage of viable Caco-2 or Vero cells measured as the average absorbance relative to DMSO after exposure to the tested compounds. Dimethyl sulfoxide (DMSO) was used as a negative control. Error bars represent the standard deviation values.

Mammalian toxicity is the main obstacle that prevents the development of new antifungal drugs due to the similarity between fungal and human cells as they are both eukaryotic cells. The currently available antifungals still have great toxicity in clinical applications [34]. For instance, the azoles are limited by their hepatotoxicity, which has resulted in discontinuation of therapy in some patients. Additionally, amphotericin B is associated with a major side effect, nephrotoxicity, which limits its use [35]. Consequently, the preliminary safety profiles of the top three bisphenylthiazole candidates, compounds **7**, **16** and **17**, were assessed using two mammalian cell lines. In general, the three tested compounds (**7**, **16** and **17)** were found to be non-toxic to Caco-2 and Vero cells at concentrations as high as 64 μg/mL, as shown by their 50% cytotoxic concentration (CC_50_), which is the concentration of the compound that leads to 50% viability of the treated cells (Fig 2). Interestingly, regardless of the high matching between the *cis*- and *trans*-diaminocyclohexyl derivatives of compounds **16** and **17** in their MIC values, the *trans* analog exhibited better tolerability by Caco-2 cells because its CC_50_ exceeded the limit of 128 μg/mL (Fig 2). In all cases, all tested compounds (**7**, **16** and **17)** were obviously less toxic than amphotericin B, which exhibited toxicity in Vero cells at a very low concentration (8 μg/mL), (S1 Fig) in agreement with previous reports [36–38].

#### 2.2.4. Antifungal synergy measurement by checkerboard testing

**Fig 3:**
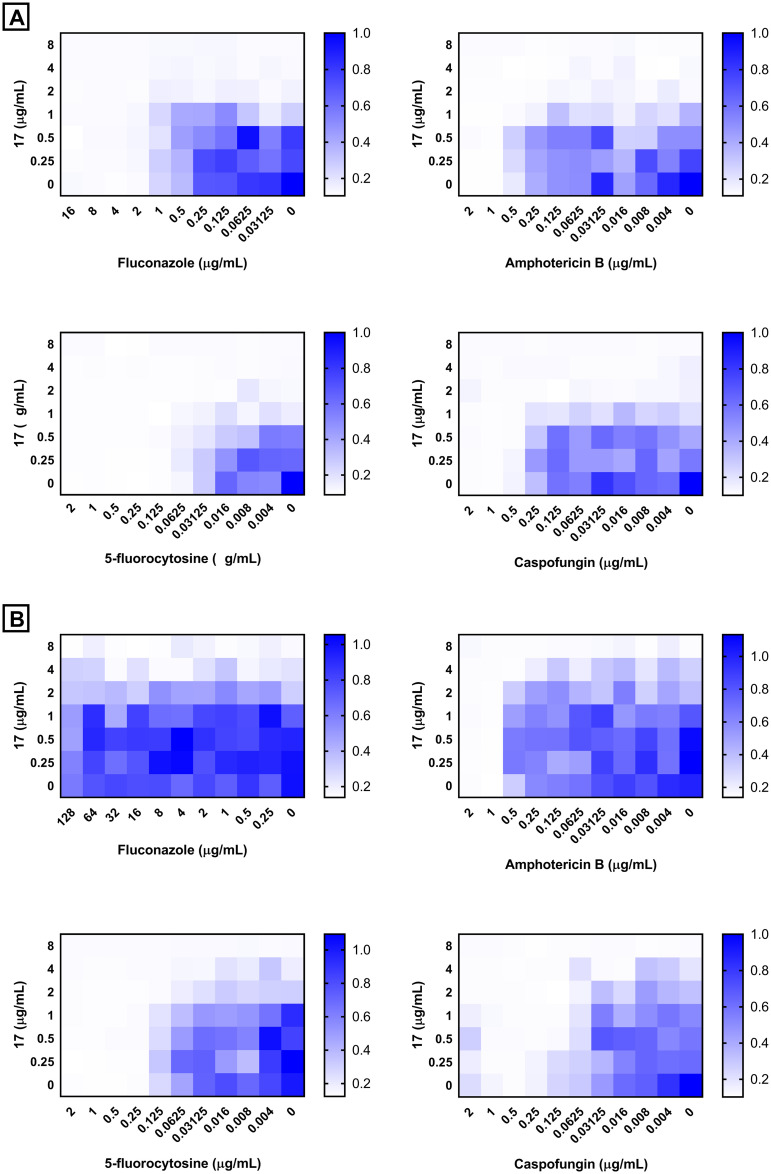
Interactions between compound 17 and the standard antifungals (fluconazole, amphotericin B, 5-fluorocytosine and caspofungin) against: A) fluconazole-sensitive *Candida auris* 381, and B) fluconazole-resistant *Candida auris* 385. A checkerboard assay was performed and bacterial growth (OD_600_), relative to the negative control, was measured using a spectrophotometer.

Combination therapy has become a standard for several diseases. This strategy is currently often used in the healthcare setting to exploit the advantages of drugs combinations such as different mechanisms of action, lower toxicity, potential synergism and less probability of development of resistance to both agents [39, 40]. Given the increasing morbidity and mortality associated with invasive fungal infections, treatment with a combination of antifungal agents is often considered [41]. We investigated the interactions between compound **17** and the conventional antifungal drugs using a checkerboard assay. As presented in Fig 3, compound **17** displayed an additive relationship when combined with either fluconazole, 5-fluorocytosine or caspofungin against the two tested *C*. *auris* isolates with a FICI ranging from 1.001 to 1.25 (S1 File). Since the treatment course of most invasive fungal infections is long, combination therapy is highly desirable to decrease the probability of developing resistance and the toxicity of the administered antifungals.

## 3. Conclusion

We examined the SAR at the oxadiazole-C2 and introduced two antifungal bisphenylthiazoles (compounds **16** and **17)** with optimized action and improved properties. Both compounds exhibited potent fungistatic activity against a panel of clinically-relevant fungal isolates including multi-drug resistant *C*. *auris*. Furthermore, their toxicity profile was better than that of amphotericin B. The active compounds developed in this study were selective for fungal cells without disrupting the representative normal human microbiota. Moreover, these compounds acted additively with the standard antifungal agents.

## 4. Materials and methods

### 4.1. General chemistry

The purity of all biologically-tested compounds was confirmed to be at least 95% using elemental analysis. All starting material chemicals and solvents were obtained from commercial sources. The masses were weighed on a microbalance with a resolution of 0.0001 g. Visualization on TLC was performed using UV light (254 nm). ^1^H NMR spectra were run at 400 MHz, and ^13^C spectra were determined at 100 MHz in deuterated dimethyl sulfoxide (DMSO-*d*_*6*_) on a Varian Mercury VX-400 NMR spectrometer. Chemical shifts are given in parts per million (ppm) on the delta (δ) scale. Chemical shifts were calibrated relative to those of the solvents. Flash chromatography was performed on 230–400 mesh silica. The progress of reactions was monitored with Merck silica gel IB2-F plates (0.25 mm thickness). Mass spectra were recorded at 70 eV. High-resolution mass spectra for all ionization techniques were obtained from a FinniganMAT XL95. Melting points were determined using capillary tubes with a Stuart SMP30 apparatus and are uncorrected. Microanalyses for C, H and N were performed at the Regional Center for Mycology and Biotechnology, Al-Azhar University. All yields reported referring to isolated yields. Compounds **3–6** were prepared as reported elsewhere [42].

#### 4.1.1. Compounds 7–21

*4*.*1*.*1*.*1*. *General procedure*. The methylsulfone **6** (0.1 g, 0.26 mmol) was stirred in dry DMF (5 mL) to complete dissolution, and then a proper alkylamine (0.4 mmol), used as obtained for the commercial source, was added. The reaction mixtures were flushed with dry nitrogen and heated to 80 °C. The progress of the reaction was monitored by TLC. After consuming all starting materials in a period ranging from 0.5 to 12 h, the reaction mixture was allowed to cool to room temperature and quenched with ice water (50 mL). The formed solids were filtered, washed with 50% ethanol and purified by crystallization from absolute ethanol to yield the desired products. The yields, physical properties and spectral analyses of isolated products are listed below:

#### 4.1.2. *N*-{5-[2-((1,1’-Biphenyl)-4-yl)-4-methylthiazol-5-yl]-1,3,4-oxadiazol-2-yl}ethane-1,2-diamine (7)

Following the general procedure, and using ethylenediamine (21 μL, 0.4 mmol), compound **7** was obtained as yellow solid (0.06 g, 66%) mp = 145–146 °C; ^1^H NMR (DMSO-*d*_*6*_) δ: 8.06 (d, *J* = 8.4 Hz, 2H), 7.93 (brs, 1H), 7.81 (d, *J* = 8.4 Hz, 2H), 7.71 (d, *J* = 7.2 Hz, 2H), 7.48 (t, *J* = 7.6 Hz, 2H), 7.38 (t, *J* = 6.8 Hz, 1H), 3.57–3.53 (m, 2H), 2.65 (s, 3H), 2.63–2.58 (m, 2H), 1.20 (brs, 2H); MS (*m/z*) 377; HRMS (EI) *m/z* 377.1310 M^+^, calcd for C_20_H_19_N_5_OS 377.1310; Anal. Calc. for: (C_20_H_19_N_5_OS): C, 63.64; H, 5.07; N, 18.55%; Found: C, 63.64; H, 5.07; N, 18.55%.

#### 4.1.3. N-{5-[2-((1,1’-Biphenyl)-4-yl)-4-methylthiazol-5-yl]-1,3,4-oxadiazol-2-yl}-N,N-dimethylethane-1,2-diamine (8)

Following the general procedure, and using *N*,*N*-dimethylenediamine (35 μL, 0.4 mmol), compound **8** was obtained as yellow solid (0.1 g, 98%) mp = 98 °C; ^1^H NMR (DMSO-*d*_*6*_) δ: 8.03 (d, *J* = 8.4 Hz, 2H), 7.80 (d, *J* = 8.4 Hz, 2H), 7.74 (d, *J* = 7.2 Hz, 2H), 7.51 (t, *J* = 7.6 Hz, 2H), 7.42 (t, *J* = 6.8 Hz, 1H), 6.93 (brs, 1H), 3.42–3.39 (m, 2H), 2.69 (s, 3H), 2.44–2.38 (m, 2H), 2.18 (s, 6H); MS (*m/z*) 405; HRMS (EI) *m/z* 405.1623 M^+^, calcd for C_22_H_23_N_5_OS 405.1623; Anal. Calc. for: (C_22_H_23_N_5_OS): C, 65.16; H, 5.72; N, 17.27%; Found: C, 65.16; H, 5.72; N, 17.27%.

#### 4.1.4. 2-{[5-(2-((1,1’-biphenyl)-4-yl)-4-methylthiazol-5-yl)-1,3,4-oxadiazol-2-yl]amino} acetimidamide (9)

Following the general procedure, using 2-aminoacetimidamide dihydrobromide (93 mg, 0.4 mmol) and potassium carbonate anhydrous (0.1 g, 0.7 mmol), compound **9** was obtained as brown solid (0.06 g, 58%) mp = 292–294 °C; ^1^H NMR (DMSO-*d*_*6*_) δ: ^1^H NMR (DMSO-*d*_*6*_) δ: 8.06 (d, *J* = 8.4 Hz, 2H), 7.96 (brs, 2H), 7.85 (d, *J* = 8.4 Hz, 2H), 7.73 (d, *J* = 7.2 Hz, 2H), 7.53 (t, *J* = 7.6 Hz, 2H), 7.43 (t, *J* = 6.8 Hz, 1H), 6.68 (brs, 1H), 3.83 (s, 2H), 2.68 (s, 3H); MS (*m/z*) 390; HRMS (EI) *m/z* 390.1263 M^+^, calcd for C_20_H_18_N_6_OS 390.1263; Anal. Calc. for: (C_20_H_18_N_6_OS): C, 61.52; H, 4.65; N, 21.52%; Found: C, 61.52; H, 4.65; N, 21.52%.

#### 4.1.5. 5-{2-[(1,1’-Biphenyl)-4-yl]-4-methylthiazol-5-yl}-N-(pyrrolidin-3-yl)-1,3,4-oxadiazol-2-amine (10)

Following the general procedure, using 3-amino-pyrrolidine dihydrochloride (60 mg, 0.4 mmol) and potassium carbonate anhydrous (0.1 g, 0.7 mmol), compound **10** was obtained as yellow solid (0.07 g, 76%) mp = 116–117 °C; ^1^H NMR (DMSO-*d*_*6*_) δ: 8.05 (d, *J* = 8.4 Hz, 2H), 7.82 (d, *J* = 8.4 Hz, 2H), 7.74 (d, *J* = 7.2 Hz, 2H), 7.51 (t, *J* = 7.6 Hz, 2H), 7.42 (t, *J* = 6.8 Hz, 1H), 3.63–3.51 (m, 3H), 2.72 (s, 3H), 2.07–2.05 (m, 2H), 1.77–1.76 (m, 2H), 1.21 (brs, 2H); MS (*m/z*) 403; HRMS (EI) *m/z* 403.1467 M^+^, calcd for C_22_H_21_N_5_OS 403.1467; Anal. Calc. for: (C_22_H_21_N_5_OS): C, 65.49; H, 5.25; N, 17.36%; Found: C, 65.49; H, 5.25; N, 17.36%.

#### 4.1.6. (R)-1-{5-[2-((1,1’-biphenyl)-4-yl)-4-methylthiazol-5-yl]-1,3,4-oxadiazol-2-yl}-N,N-dimethylpyrrolidin-3-amine (11)

Following the general procedure, and using ((*R*)-(+)-3-(dimethylamino)pyrrolidine dihydrochloride (74 mg, 0.4 mmol) and potassium carbonate anhydrous (0.1 g, 0.7 mmol), compound **11** was obtained as yellow solid (0.08 g, 79%) mp = 165–167 °C; ^1^H NMR (DMSO-*d*_*6*_) δ: 8.06 (d, *J* = 8.4 Hz, 2H), 7.83 (d, *J* = 8.4 Hz, 2H), 7.75 (d, *J* = 7.2 Hz, 2H), 7.51 (t, *J* = 7.6 Hz, 2H), 7.42 (t, *J* = 6.8 Hz, 1H), 3.68–3.65 (m, 3H), 3.50–3.48 (m, 2H), 2.87–2.84 (m, 1H), 2.70 (s, 3H), 2.18 (s, 6H), 1.87–1.85 (m, 1H); MS (*m/z*) 431; HRMS (EI) *m/z* 431.1780 M^+^, calcd for C_24_H_25_N_5_OS 431.1780; Anal. Calc. for: (C_24_H_25_N_5_OS): C, 66.80; H, 5.84; N, 16.23%; Found: C, 66.80; H, 5.84; N, 16.23%.

#### 4.1.7. (S)-1-{5-[2-((1,1’-biphenyl)-4-yl)-4-methylthiazol-5-yl]-1,3,4-oxadiazol-2-yl}-N,N-dimethylpyrrolidin-3-amine (12)

Following the general procedure, and using (*S*)-(-)-3-(dimethylamino)pyrrolidine (45 μL, 0.4 mmol), compound **12** was obtained as yellow solid (0.06 g, 61%) mp = 185–187 °C; ^1^H NMR (DMSO-*d*_*6*_) δ: 8.05 (d, *J* = 8.4 Hz, 2H), 7.85 (d, *J* = 8.4 Hz, 2H), 7.74 (d, *J* = 7.2 Hz, 2H), 7.51 (t, *J* = 7.6 Hz, 2H), 7.40 (t, *J* = 6.8 Hz, 1H), 3.67–3.60 (m, 3H), 3.51–3.44 (m, 2H), 2.86–2.83 (m, 1H), 2.69 (s, 3H), 2.18 (s, 6H), 1.85–1.80 (m, 1H); MS (*m/z*) 431; HRMS (EI) *m/z* 431.1780 M^+^, calcd for C_24_H_25_N_5_OS 431.1780; Anal. Calc. for: (C_24_H_25_N_5_OS): C, 66.80; H, 5.84; N, 16.23%; Found: C, 66.80; H, 5.84; N, 16.23%.

#### 4.1.8. (S)-1-{5-[2-((1,1’-biphenyl)-4-yl)-4-methylthiazol-5-yl]-1,3,4-oxadiazol-2-yl}pyro-lidine-2-carboxamide (13)

Following the general procedure, and using L-prolinamide (45 mg, 0.4 mmol), compound **13** was obtained as yellow solid (0.08 g, 77%) mp = 115–116 °C; ^1^H NMR (DMSO-*d*_*6*_) δ: 8.06 (d, *J* = 8.4 Hz, 2H), 7.83 (d, *J* = 8.4 Hz, 2H), 7.74 (d, *J* = 7.2 Hz, 2H), 7.51 (t, *J* = 7.6 Hz, 2H), 7.40 (t, *J* = 6.8 Hz, 1H), 7.16 (brs, 2H), 4.29–4.23 (m, 1H), 3.63–3.56 (m, 2H), 2.65 (s, 3H), 2.27–2.25 (m, 1H), 1.96–1.93 (m, 3H); MS (*m/z*) 431; HRMS (EI) *m/z* 431.1416 M^+^, calcd for C_23_H_21_N_5_O_2_S 431.1416; Anal. Calc. for: (C_23_H_21_N_5_O_2_S): C, 64.02; H, 4.91; N, 16.23%; Found: C, 64.02; H, 4.91; N, 16.23%.

#### 4.1.9. (R)-1-{5-[2-((1,1’-biphenyl)-4-yl)-4-methylthiazol-5-yl]-1,3,4-oxadiazol-2-yl}pyro-lidine-2-carboxamide (14)

Following the general procedure, and using D-prolinamide (45 mg, 0.4 mmol), compound **14** was obtained as yellow solid (0.08 g, 79%) mp = 119–120 °C; ^1^H NMR (DMSO-*d*_*6*_) δ:8.05 (d, *J* = 8.4 Hz, 2H), 7.83 (d, *J* = 8.4 Hz, 2H), 7.74 (d, *J* = 7.2 Hz, 2H), 7.51 (t, *J* = 7.6 Hz, 2H), 7.47 (t, *J* = 6.8 Hz, 1H), 7.16 (brs, 2H), 4.29–4.27 (m, 1H), 3.63–3.53 (m, 2H), 2.67 (s, 3H), 2.29–2.25 (m, 1H), 1.97–1.93 (m, 3H); MS (*m/z*) 431; HRMS (EI) *m/z* 431.1416 M^+^, calcd for C_23_H_21_N_5_O_2_S 431.1416; Anal. Calc. for: (C_23_H_21_N_5_O_2_S): C, 64.02; H, 4.91; N, 16.23%; Found: C, 64.02; H, 4.91; N, 16.23%.

#### 4.1.10. {1-[5-(2-((1,1’-Biphenyl)-4-yl)-4-methylthiazol-5-yl)-1,3,4-oxadiazol-2-yl]piperidin-2-yl}methanamine (15)

Following the general procedure, and using 2-(aminomethyl)piperidine (45 μL, 0.4 mmol), compound **15** was obtained as yellow solid (0.06 g, 59%) mp = 225–227 °C; ^1^H NMR (DMSO-*d*_*6*_) δ: 8.05 (d, *J* = 8.4 Hz, 2H), 7.83 (d, *J* = 8.4 Hz, 2H), 7.74 (d, *J* = 7.2 Hz, 2H), 7.51 (t, *J* = 7.6 Hz, 2H), 7.42 (t, *J* = 6.8 Hz, 1H), 3.85–3.82 (m, 1H), 3.15–3.13 (m, 2H), 2.69 (s, 3H), 1.73–1.49 (m, 7H), 1.29 (brs, 2H), 1.08–1.03 (m, 1H); MS (*m/z*) 431; HRMS (EI) *m/z* 431.1780 M^+^, calcd for C_24_H_25_N_5_OS 431.1780; Anal. Calc. for: (C_24_H_25_N_5_OS): C, 66.80; H, 5.84; N, 16.23%; Found: C, 66.80; H, 5.84; N, 16.23%.

#### 4.1.11. N-{5-[2-((1,1’-Biphenyl)-4-yl)-4-methylthiazol-5-yl]-1,3,4-oxadiazol-2-yl}cyclo-hexane-trans-1,2-diamine (16)

Following the general procedure, and using (±)-*trans*-1,2-diaminocyclohexane (45 μL, 0.4 mmol), compound **16** was obtained as orange solid (0.08 g, 82%) mp = 180–183 °C; ^1^H NMR (DMSO-*d*_*6*_) δ: 7.98 (d, *J* = 8.4 Hz, 2H), 7.81 (d, *J* = 8.4 Hz, 2H), 7.73 (d, *J* = 7.2 Hz, 2H), 7.48 (t, *J* = 7.6 Hz, 2H), 7.42 (t, *J* = 6.8 Hz, 1H), 6.71 (brs, 1H), 3.85–3.83 (m, 2H), 2.70 (s, 3H), 2.10–1.99 (m, 2H), 1.88 (brs, 2H), 1.72–1.64 (m, 2H), 1.30–1.23 (m, 4H); MS (*m/z*) 431; HRMS (EI) *m/z* 431.1780 M^+^, calcd for C_24_H_25_N_5_OS 431.1780; Anal. Calc. for: (C_24_H_25_N_5_OS): C, 66.80; H, 5.84; N, 16.23%; Found: C, 66.80; H, 5.84; N, 16.23%.

#### 4.1.12. *cis*-*N*-{5-[2-((1,1’-Biphenyl)-4-yl)-4-methylthiazol-5-yl]-1,3,4-oxadiazol-2-yl}cyclo-hexane-1,2-diamine (17)

Following the general procedure, and using (±)-*cis*-1,2-diaminocyclohexane (45 μL, 0.4 mmol), compound **17** was obtained as brown solid (0.08 g, 85%) mp = 145–146 °C; ^1^H NMR (DMSO-*d*_*6*_) δ: 7.95 (d, *J* = 8.4 Hz, 2H), 7.78 (d, *J* = 8.4 Hz, 2H), 7.72 (d, *J* = 7.2 Hz, 2H), 7.47 (t, *J* = 7.6 Hz, 2H), 7.39 (t, *J* = 6.8 Hz, 1H), 7.03 (brs, 1H), 3.74–3.72 (m, 2H), 2.87–2.85 (m, 2H), 2.68 (s, 3H), 1.80–1.69 (m, 2H), 1.44 (brs, 2H), 1.26–1.20 (m, 4H); MS (*m/z*) 431; HRMS (EI) *m/z* 431.1780 M^+^, calcd for C_24_H_25_N_5_OS 431.1780; Anal. Calc. for: (C_24_H_25_N_5_OS): C, 66.80; H, 5.84; N, 16.23%; Found: C, 66.80; H, 5.84; N, 16.23%.

#### 4.1.13. trans-N-{5-[2-((1,1’-Biphenyl)-4-yl)-4-methylthiazol-5-yl]-1,3,4-oxadiazol-2-yl}cyclo-hexane-1,4-diamine (18)

Following the general procedure, and using *trans*-1,4-diaminocyclohexane (45 mg, 0.4 mmol), compound **18** was obtained as yellow solid (0.08 g, 80%) mp = 260–263 °C; ^1^H NMR (DMSO-*d*_*6*_) δ: 8.05 (d, *J* = 8.4 Hz, 2H), 7.93 (brs, 1H), 7.83 (d, *J* = 8.4 Hz, 2H), 7.78 (d, *J* = 7.2 Hz, 2H), 7.51 (t, *J* = 7.6 Hz, 2H), 7.40 (t, *J* = 6.8 Hz, 1H), 3.80–3.78 (m, 2H), 2.69 (s, 3H), 2.05–2.03 (m, 4H), 1.91–1.85 (m, 4H), 1.28 (brs, 2H); MS (*m/z*) 431; HRMS (EI) *m/z* 431.1780 M^+^, calcd for C_24_H_25_N_5_OS 431.1780; Anal. Calc. for: (C_24_H_25_N_5_OS): C, 66.80; H, 5.84; N, 16.23%; Found: C, 66.80; H, 5.84; N, 16.23%.

#### 4.1.14. 2-{[5-(2-((1,1’-Biphenyl)-4-yl)-4-methylthiazol-5-yl)-1,3,4-oxadiazol-2-yl]amino} ethan-1-ol (19)

Following the general procedure, and using 2-aminoethan-1-ol (21 μL, 0.4 mmol), compound **19** was obtained as yellow solid (0.06 g, 65%) mp = 210–213 °C; ^1^H NMR (DMSO-*d*_*6*_) δ: 8.06 (d, *J* = 8.4 Hz, 2H), 7.90 (brs, 1H), 7.83 (d, *J* = 8.4 Hz, 2H), 7.73 (d, *J* = 7.2 Hz, 2H), 7.51 (t, *J* = 7.6 Hz, 2H), 7.41 (t, *J* = 6.8 Hz, 1H), 4.78 (brs, 1H), 3.58–3.53 (m, 2H), 3.38–3.35 (m, 2H), 2.68 (s, 3H); MS (*m/z*) 378; HRMS (EI) *m/z* 378.1150 M^+^, calcd for C_20_H_18_N_4_O_2_S 378.1150; Anal. Calc. for: (C_20_H_18_N_4_O_2_S): C, 63.47; H, 4.79; N, 14.80%; Found: C, 63.47; H, 4.79; N, 14.80%.

#### 4.1.15. (S)-{1-[5-(2-((1,1’-Biphenyl)-4-yl)-4-methylthiazol-5-yl)-1,3,4-oxadiazol-2-yl]pyro-lidin-2-yl}methanol (20)

Following the general procedure, and using (*S*)-(+)-2-pyrrolidinemethanol (40 μL, 0.4 mmol), compound **20** was obtained as yellow solid (0.06 g, 61%) mp = 174–175 °C; ^1^H NMR (DMSO-*d*_*6*_) δ: 8.06 (d, *J* = 8.4 Hz, 2H), 7.83 (d, *J* = 8.4 Hz, 2H), 7.76 (d, *J* = 7.2 Hz, 2H), 7.51 (t, *J* = 7.6 Hz, 2H), 7.41 (t, *J* = 6.8 Hz, 1H), 4.88 (brs, 1H), 3.93–3.90 (m, 1H), 3.55–3.48 (m, 2H), 3.43–3.41 (m, 2H), 2.70 (s, 3H), 2.05–1.90 (m, 4H); MS (*m/z*) 418; HRMS (EI) *m/z* 418.1463 M^+^, calcd for C_23_H_22_N_4_O_2_S 418.1463; Anal. Calc. for: (C_23_H_22_N_4_O_2_S): C, 66.01; H, 5.30; N, 13.39%; Found: C, 66.01; H, 5.30; N, 13.39%.

#### 4.1.16. (R)-{1-[5-(2-((1,1’-Biphenyl)-4-yl)-4-methylthiazol-5-yl)-1,3,4-oxadiazol-2-yl]pyro-lidin-2-yl}tmethanol (21)

Following the general procedure, and using (*R*)-(-)-2-pyrrolidinemethanol (40 μL, 0.4 mmol), compound **21** was obtained as yellow solid (0.07 g, 69%) mp = 230–231 °C; ^1^H NMR (DMSO-*d*_*6*_) δ: 8.06 (d, *J* = 8.4 Hz, 2H), 7.83 (d, *J* = 8.4 Hz, 2H), 7.76 (d, *J* = 7.2 Hz, 2H), 7.53 (t, *J* = 7.6 Hz, 2H), 7.41 (t, *J* = 6.8 Hz, 1H), 4.85 (brs, 1H), 3.93–3.90 (m, 1H), 3.60–3.58 (m, 2H), 3.38–3.36 (m, 2H), 2.70 (s, 3H), 2.08–1.92 (m, 4H); MS (*m/z*) 418; HRMS (EI) *m/z* 418.1463 M^+^, calcd for C_23_H_22_N_4_O_2_S 418.1463; Anal. Calc. for: (C_23_H_22_N_4_O_2_S): C, 66.01; H, 5.30; N, 13.39%; Found: C, 66.01; H, 5.30; N, 13.39%.

### 4.2. Microbial strains, cell lines and culture media

The bacterial and fungal strains (S2 Table in S1 File) used in this study were clinical isolates obtained from the American Type Culture Collection (ATCC, Manassas, VA, USA), the Centers for Disease Control and Prevention (CDC) and Biodefense and Emerging Infections Research Resources Repository (BEI Resources) (Manassas, VA, USA). RPMI 1640 (Thermo Fisher Scientific, Waltham, MA), YPD broth, YPD agar, cation adjusted Mueller-Hinton broth, brain heart infusion broth, and lactobacilli MRS broth (Becton, Dickinson and Company, Franklin Lakes, NJ) were purchased from commercial vendors. Phosphate buffered saline was purchased from Fisher Scientific (Waltham, MA). Yeast extract, L-cysteine, vitamin K, 3-(*N*-Morpholino)propanesulfonic acid (MOPS) and hemin were obtained from Sigma-Aldrich (St. Louis, MO). Human colorectal adenocarcinoma epithelial cells (Caco-2) (ATCC HTB-37), and monkey kidney epithelial cells (Vero) (ATCC CCL-81-VHG) were obtained from the American Type Culture Collection (ATCC) (Manassas, VA, USA).

#### 4.2.1. Initial screening of the bisphenylthiazoles against Gram-positive and Gram-negative bacteria

The minimum inhibitory concentrations (MICs) of the tested compounds and control drugs against clinically-relevant bacterial strains were determined using the broth microdilution method, according to guidelines outlined by the Clinical and Laboratory Standards Institute (CLSI) [43], or as described previously [10].

#### 4.2.2. Determination of the minimum inhibitory concentration (MIC) of bisphenylthiazoles against a panel of *Candida*, *Cryptococcus* and *Aspergillus* strains and determination of the minimum fungicidal concentration (MFC)

Serial dilutions (from 128 μg/mL to 0.06 μg/mL) of the tested compounds and control drugs (fluconazole, itraconazole and amphotericin) (in triplicates), were screened against clinical isolates of *Candida albicans*, *Candida auris*, *Candida glabrata*, *Candida parapsilosis*, *Candida tropicalis*, *Candida krusei*, *Cryptococcus neoformans*, *Cryptococcus gattii* and *Aspergillus fumigatus* species using the broth microdilution method according to the Clinical and Laboratory Standards Institute guidelines [44]. MICs were determined as the lowest concentration of the compounds that inhibited the fungal growth by 50% after incubation at 35 °C for 24 h (for *Candida* and *Aspergillus*), or 48 h (for *Cryptococcus*). The minimum fungicidal concentration (MFC) of these compounds was tested by plating 4 μL from wells with no growth onto YPD agar plates. The plates were incubated at 37 ºC for 24 hours before recording the MFC, which was categorized as the lowest concentration that reduced fungal growth by 99.9% [45]. The experiment was repeated three independent times.

#### 4.2.3. Safety profile assessment of bisphenylthiazoles in Caco-2 and Vero cells

To explore the tolerability of bisphenylthiazoles, their cytotoxicity profile was assessed in human colorectal adenocarcinoma (Caco-2) and monkey kidney epithelial cells (Vero) as described earlier [8, 46–48]. Briefly, compounds were incubated with the cells for 24 hours. Then, cells were incubated with MTS reagent for 3 hours before measuring the absorbance values (OD_490_). The experiment was repeated in two independent times.

#### 4.2.4. *In vitro* antimicrobial evaluation of bisphenylthiazoles against normal human microflora

Broth microdilution was utilized to determine the activity of bisphenylthiazoles against the human gut microflora as described elsewhere [49, 50]. A bacterial solution equivalent to 0.5 McFarland standards was prepared and diluted in MRS broth to achieve a bacterial concentration of approximately 5 x 10^5^ CFU/mL. Then, compounds/control antifungals (in triplicates), were added to the plates and serially diluted with media containing bacteria. The plates were incubated for 48 hours at 37 °C before recording the MIC values. The experiment was repeated three independent times.

#### 4.2.5. Evaluation of synergistic interactions using a checkerboard assay

To evaluate the interactions between bisphenylthiazoles and the standard antifungal agents (fluconazole, amphotericin B, 5-fluorocytosine and caspofungin) against 2 *C*. *auris* clinical isolates, a standard checkerboard assay was utilized as described in previous studies [51–53]. The fractional inhibitory concentration index (FICI) was calculated for each interaction. Interactions where the FICI was ≤0.5 were categorized as synergistic (SYN). FICI values of >0.5–1.25 were categorized as additive (ADD). FICI values of >1.25–4 were categorized as indifferent, and FICI values of > 4 were categorized as antagonistic [54]. The experiment was repeated three independent times.

## Supporting information

S1 File ^1^H and ^13^C NMR spectra of all new described compounds, description of strains used in this study, toxicity data for amphotericin B against Vero cells, and MICs and FICI values obtained in the checkerboard assay. (PDF)

S1 Fig (TIF)

## Abbreviations

MFC: minimum fungicidal concentration
MIC: minimum inhibitory concentration
NAC: non-albicans *Candida* species
