# Supplementary material for: Evaluation of bisphenylthiazoles as a promising class for combating multidrug-resistant fungal infections
Source: PLoS One. 2021 Nov 4;16(11):e0258465. doi: 10.1371/journal.pone.0258465 (PMC8568133; doi:10.1371/journal.pone.0258465)
Supplement: S1 File — (PDF) [file pone.0258465.s001.pdf]

Supporting information

Evaluation of Bisphenylthiazoles as a Promising Class for Combating multidrug resistant fungal infections

a. Mohamed Hagras[1¶], Nader S. Abutaleb[2,3,4¶], Ahmed M. [Sayed1], Ehab A. Salama[2,3], Mohamed N. Seleem[2,3,5**] and Abdelrahman S. Mayhoub[1,6*]

b. Department of Pharmaceutical Organic Chemistry, College of Pharmacy, Al-Azhar University, Cairo, Egypt.

c. Department of Comparative Pathobiology, College of Veterinary Medicine, Purdue University, West Lafayette, USA.

d. Department of Biomedical Sciences and Pathobiology, Virginia-Maryland College of Veterinary Medicine, Virginia Polytechnic Institute and State University, Blacksburg,

e. Department of Microbiology and Immunology, Faculty of Pharmacy, Zagazig University, Zagazig, Egypt.

f. Center for Emerging, Zoonotic and Arthropod-borne Pathogens, Virginia Polytechnic Institute and State University, Blacksburg, USA.

g. 6. University of Science and Technology, Nanoscience Program, Zewail City of Science and Technology, Ahmed Zewail Street, Giza, Egypt.

h. Corresponding Authors

i. ** MNS; email, seleem@vt.edu

j. * ASM; e-mail, amayhoub@azhar.edu.eg

¶ These authors contributed equally to this work.

Corresponding Authors

** MNS; email, mseleem@purdue.edu; * ASM; e-mail, amayhoub@azhar.edu.eg

Table of contents

Figure S1 S2

Table S1 S3

Table S2 S4

Methods S6

NMR spectra S8

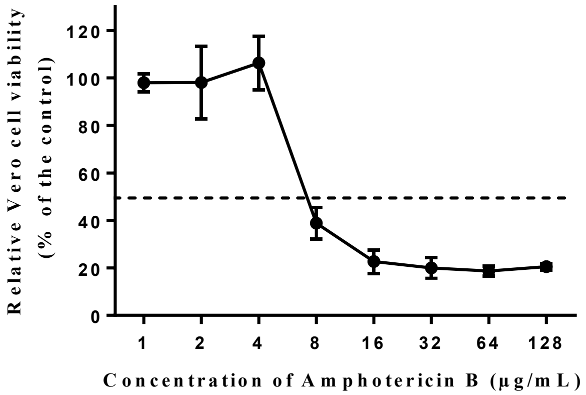

Fig S1. ***In vitro*** **cytotoxicity assay of amphotericin B in monkey kidney epithelial cells (Vero) using an MTS assay.**

Table S1. Effect of compound **17** and the standard antifungals (fluconazole, amphotericin B, 5-fluorocytosine and caspofungin) alone and in combination against: A) *Candida auris* 381 (azole-sensitive), and B) *Candida auris* 385 (azole-resistant)

A

	**MIC (μg/mL)**				**FICI***	**Interpretation**
	**Alone**		**Combined**			
	**Antifungal**	**17**	**Antifungal**	**17**		
Fluconazole	1	1	1	0.25	1.250	ADD
5-Fluorocytosine	0.125	1	0.125	0.25	1.250	ADD
Amphotericin B	1	1	1	0.25	1.250	ADD
Caspofungin	0.5	1	0.5	0.25	1.250	ADD

B

	**MIC (μg/mL)**				**FICI**	**Interpretation**
	**Alone**		**Combined**			
	**Antifungal**	**17**	**Antifungal**	**17**		
Fluconazole	>128	2	0.25	2	1.001	ADD
5-Fluorocytosine	0.25	2	0.25	0.25	1.125	ADD
Amphotericin B	1	2	1	0.25	1.125	ADD
Caspofungin	0.063	2	0.063	0.25	1.125	ADD

*FICI, fractional inhibitory concentration index.

An FICI ≤ 0.5 was considered synergistic; FICI of >0.5-1.25 was categorized as additive (ADD); FICI > 1.25 – 4 was considered indifference; and FICI > 4 was considered antagonistic.

Table S2. Microbial strains used in the study

Strains	Source/Description
**MRSA USA300**	Isolated from a wound, Mississippi, USA.<br>Community-acquired MRSA strain<br>Resistant to erythromycin and tetracycline
**C. difficile ATCC BAA 1870**	Clinical isolate<br>tcdA, tcdB and CDT genes<br>ribotype 027
***E. coli* JW55031**	*tolC-mutant*
***E. coli* BW25113**	Wild-type strain
C. albicans SS5314	Wild-type isolate
***Lactobacillus gasseri* HM-400**	Isolated from vaginal wall, Virginia, USA, 2010<br>Reference genome for the Human Microbiome Project
Lactobacillus casei ATCC**-334**	Isolated from dairy products
***Lactobacillus crispatus* HM-370**	Isolated from vaginal wall, Virginia, USA, 2010<br>Reference genome for the Human Microbiome Project
C. albicans ATCC 10231	Quality control strain<br>Isolated from bronchomycosis patient
C. albicans NR-29448	Isolated from a bloodstream infection patient, Arizona, USA
C. glabrata ATCC 66032	Quality control strain
C. glabrata ATCC MYA-2950	Quality control strain
C. parapsilosis ATCC 22019	Quality control strain<br>Isolated in Puerto Rico
C. parapsilosis CAB 502638	Isolated from human blood, Missouri, USA, 2012<br>Reference genome for the Human Microbiome Project
C. tropicalis ATCC 1369	Used in biofuel production
C. krusei CAB 396420	Isolated from human blood, Missouri, USA, 2012<br>Reference genome for the Human Microbiome Project
C. krusei ATCC 34135	Clinical isolate<br>Isolated in Minnesota, USA
C. neoformans NR 41298	Isolated from human cerebrospinal fluid, China, 2012
C. neoformans NR 41300	Isolated from human cerebrospinal fluid, China, 2011
C. neoformans NR 48770	Isolated from human cerebrospinal fluid, North Carolina, USA, 1978
C. gattii NR 43210	Obtained from a genotypic cross between *C. gattii* R265 and *C. gattii* CBS1930.
C. gattii NR 43209	Isolated from a goat in Aruba, Canada.

***C. auris* 381**	Obtained from CDC & FDA antibiotic resistance isolate bank
***C. auris* 382**	Obtained from CDC & FDA antibiotic resistance isolate bank
***C. auris* 383**	Obtained from CDC & FDA antibiotic resistance isolate bank<br>Fluconazole-resistant
***C. auris* 384**	Obtained from CDC & FDA antibiotic resistance isolate bank<br>Resistant to fluconazole, caspofungin, anidulafungin and micafungin
***C. auris* 385**	Obtained from CDC & FDA antibiotic resistance isolate bank<br>Fluconazole-resistant
***C. auris* 386**	Obtained from CDC & FDA antibiotic resistance isolate bank<br>Fluconazole-resistant
***C. auris* 387**	Obtained from CDC & FDA antibiotic resistance isolate bank
***C. auris* 388**	Obtained from CDC & FDA antibiotic resistance isolate bank<br>Fluconazole-resistant
***C. auris* 389**	Obtained from CDC & FDA antibiotic resistance isolate bank<br>Resistant to fluconazole and flucytosine
***C. auris* 390**	Obtained from CDC & FDA antibiotic resistance isolate bank<br>Resistant to fluconazole and flucytosine
A. fumigatus NR 35303	Isolated from human sputum, California, USA, 1998
A. fumigatus NR 35304	Isolated from human sputum, California, USA, 1998

Methods

Initial screening of the bisphenylthiazoles against Gram-positive and Gram-negative bacteria. Bacterial strains, (except *C. difficile* that was grown anaerobically on brain heart infusion supplemented agar at 37° C for 48 hours), were grown aerobically overnight on tryptone soy agar plates at 37° C. Afterwards, a bacterial solution equivalent to 0.5 McFarland standard was prepared and diluted in cation-adjusted Mueller-Hinton broth (CAMHB) (for *S. aureus,* and *E. coli*) to achieve a bacterial concentration of about 5×10^5 CFU/mL. *C. difficile* was diluted in brain heart infusion supplemented broth, supplemented with yeast extract, hemin and vitamin K to achieve a bacterial concentration of about 5×10^5 CFU/mL. Compounds and control drugs were added in the first row of the 96-well plates, and serially diluted with the corresponding media containing bacteria. Plates were then, incubated aerobically at 37° C for 18-20 hours (for *S. aureus* and *E. coli*). *C. difficile* was incubated anaerobically at 37° C for 48 hours. MICs reported in Table (1) are the minimum concentration of the compounds and control drugs that could completely inhibit the visual growth of bacteria.

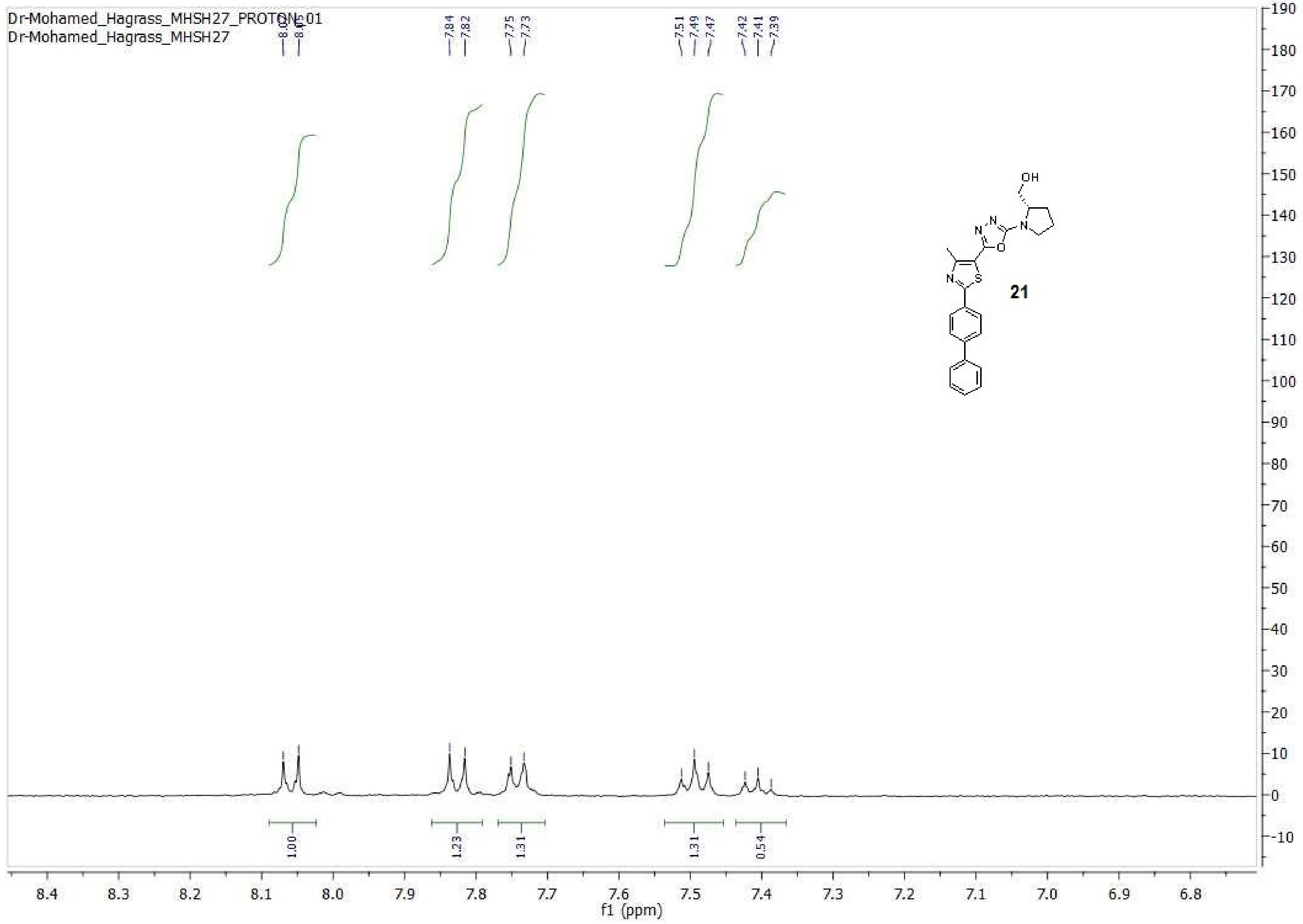
Dr-Mohamed_Hagrass_MHSH27_PROTON_01
Dr-Mohamed_Hagrass_MHSH27
21
f1 (ppm)

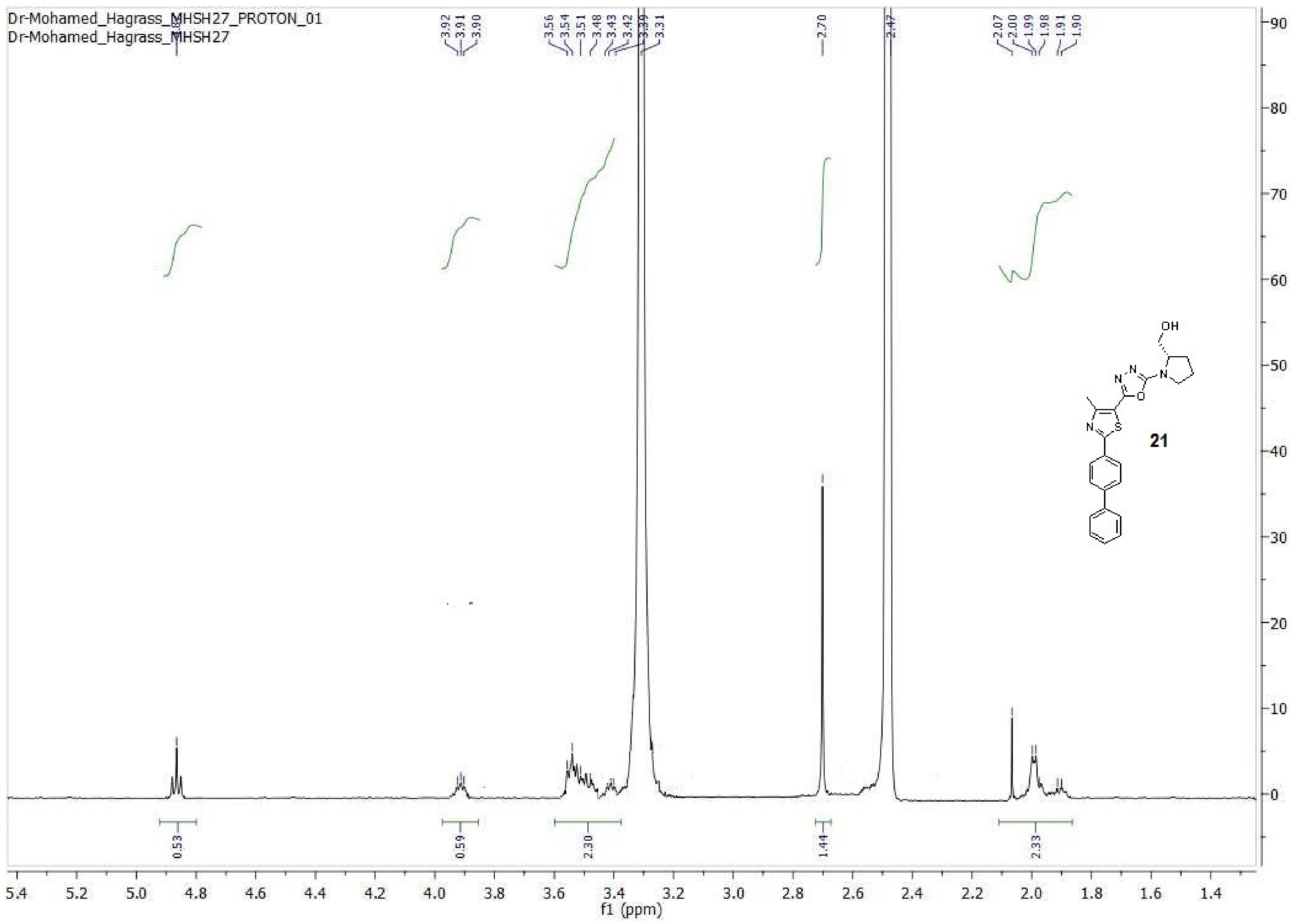
Dr-Mohamed_Hagrass_MHSH27_PROTON_01
Dr-Mohamed_Hagrass_MHSH27
21
f1 (ppm)

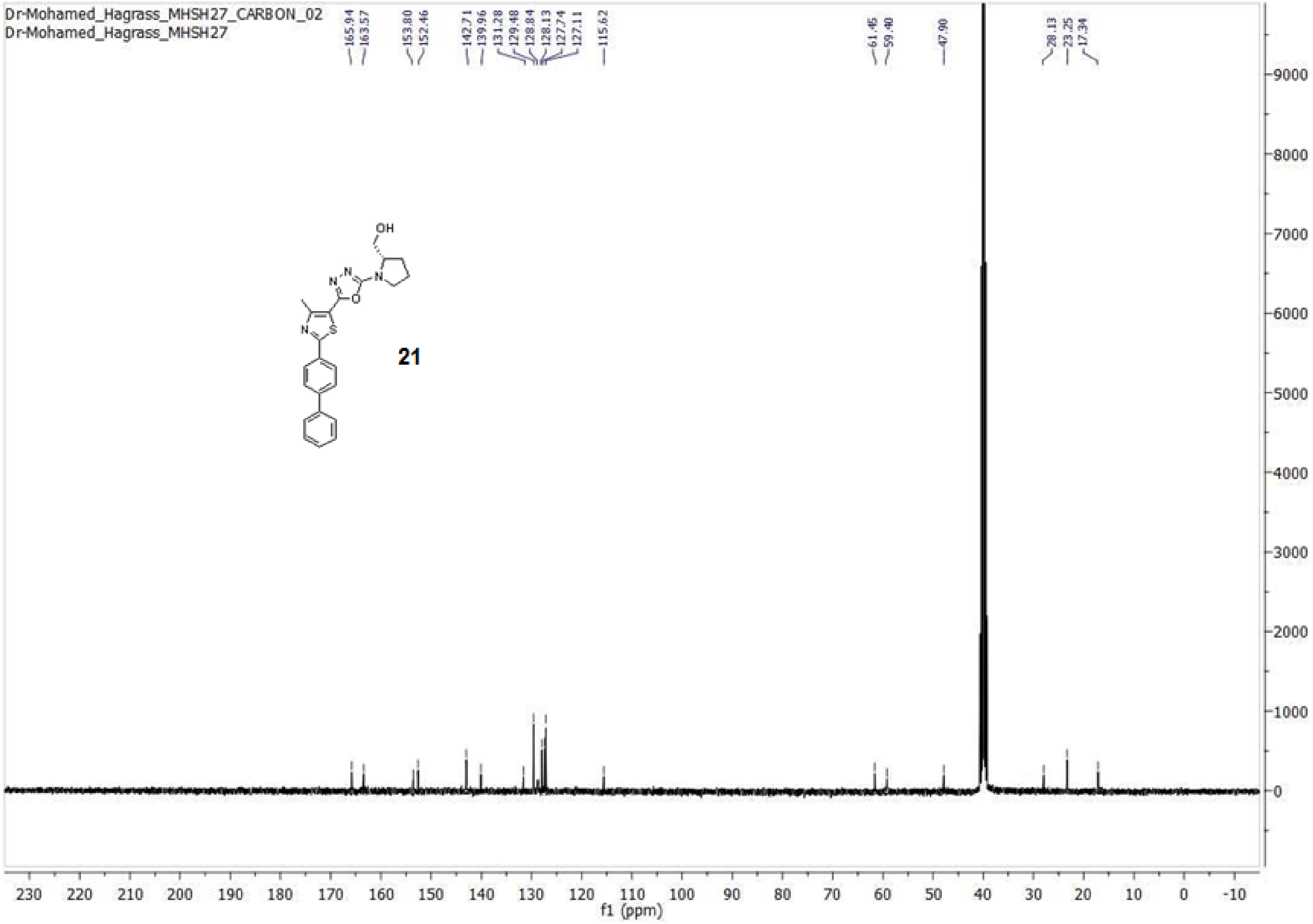
Dr-Mohamed_Hagrass_MHSH27_CARBON_02
Dr-Mohamed_Hagrass_MHSH27
165.94
163.57
153.80
152.46
142.71
139.96
131.28
129.48
128.84
128.13
127.74
127.11
115.62
61.45
59.40
47.90
28.13
23.25
17.34
OH
N
N
N
O
N
S
21
f1 (ppm)

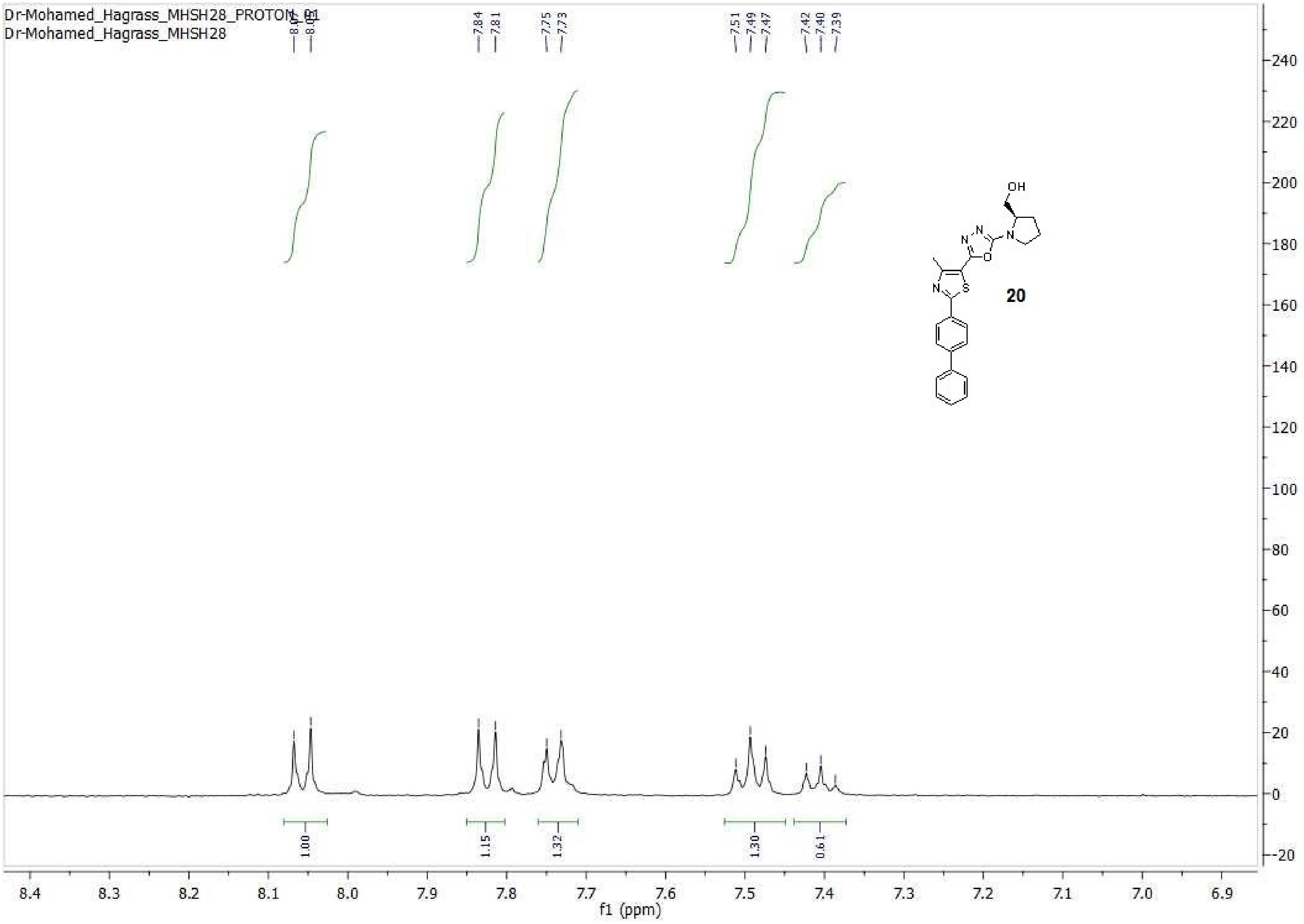
Dr-Mohamed_Hagrass_MHSH28_PROTON_01
Dr-Mohamed_Hagrass_MHSH28
7.84
7.81
7.75
7.73
7.51
7.49
7.47
7.42
7.40
7.39
OH
N
N
N
O
N
S
20
1.00
1.15
1.32
1.30
0.61
f1 (ppm)

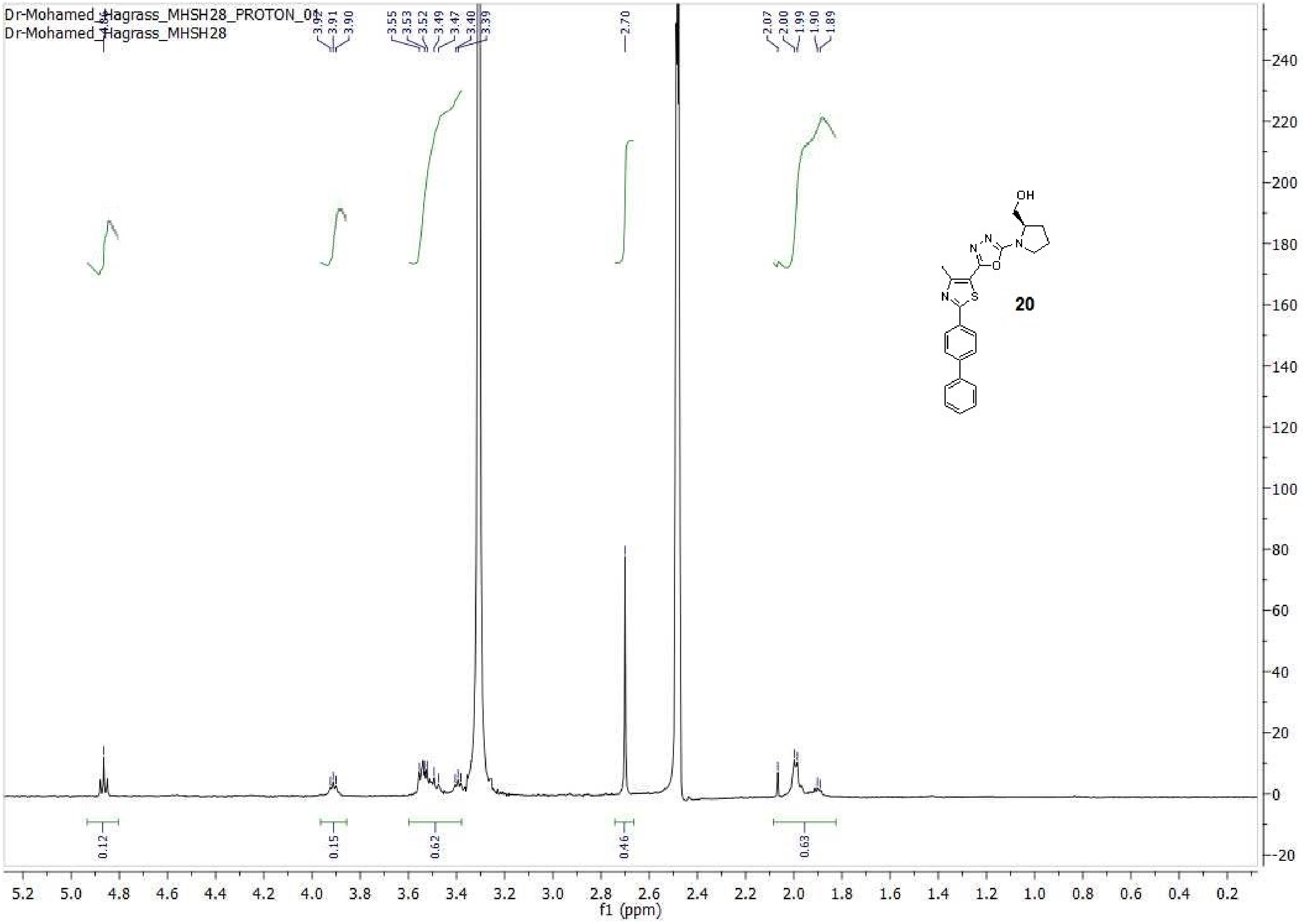
Dr-Mohamed_Hagrass_MHSH28_PROTON_01
Dr-Mohamed_Hagrass_MHSH28
3.91
3.90
3.55
3.53
3.52
3.49
3.47
3.40
3.39
2.70
2.07
2.00
1.99
1.90
1.89
OH
N
N
N
O
N
S
N
20
0.12
0.15
0.62
0.46
0.63
f1 (ppm)

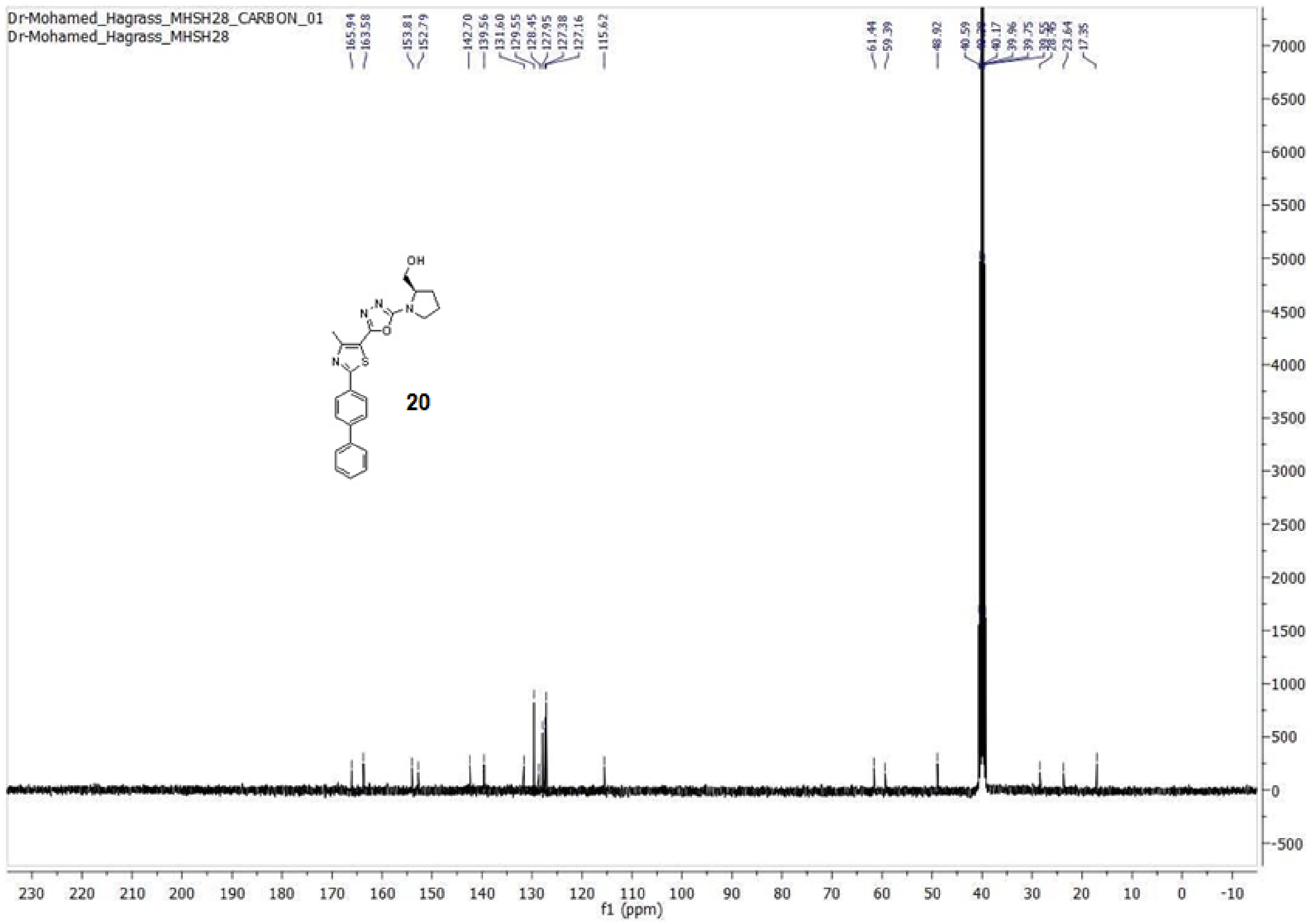

Dr-Mohamed_Hagrass_MHSH28_CARBON_01
Dr-Mohamed_Hagrass_MHSH28
165.94
163.58
153.81
152.79
142.70
139.56
131.60
129.55
128.45
127.95
127.38
127.16
115.62
61.44
59.39
48.92
40.59
40.17
39.96
39.75
23.64
17.35
OH
N
N
N
O
N
S
20
f1 (ppm)

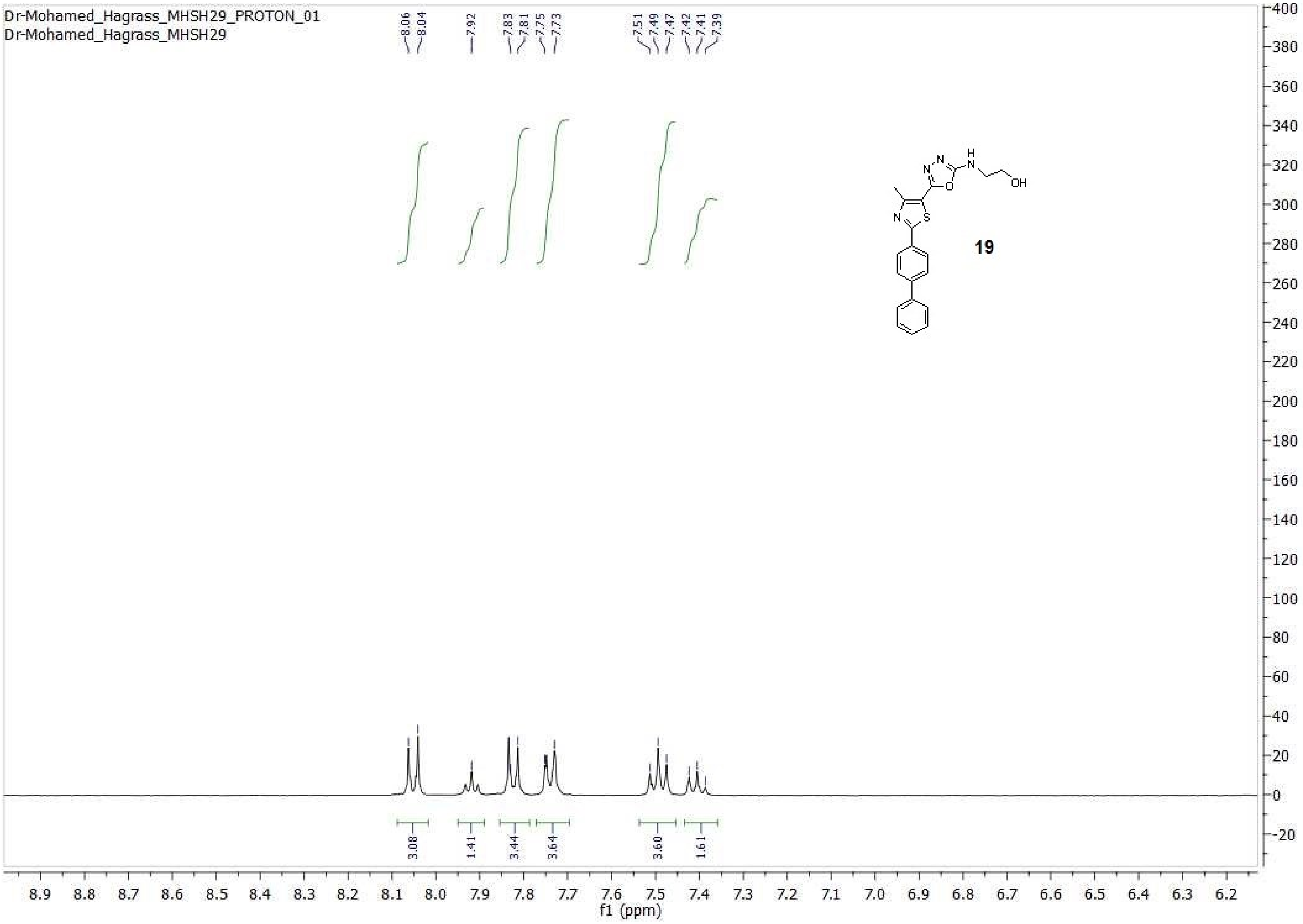
Dr-Mohamed_Hagrass_MHSH29_PROTON_01
Dr-Mohamed_Hagrass_MHSH29
8.06
8.04
7.92
7.83
7.81
7.75
7.73
7.51
7.49
7.47
7.42
7.41
7.39
19
3.08
1.41
3.44
3.64
3.60
1.61
f1 (ppm)

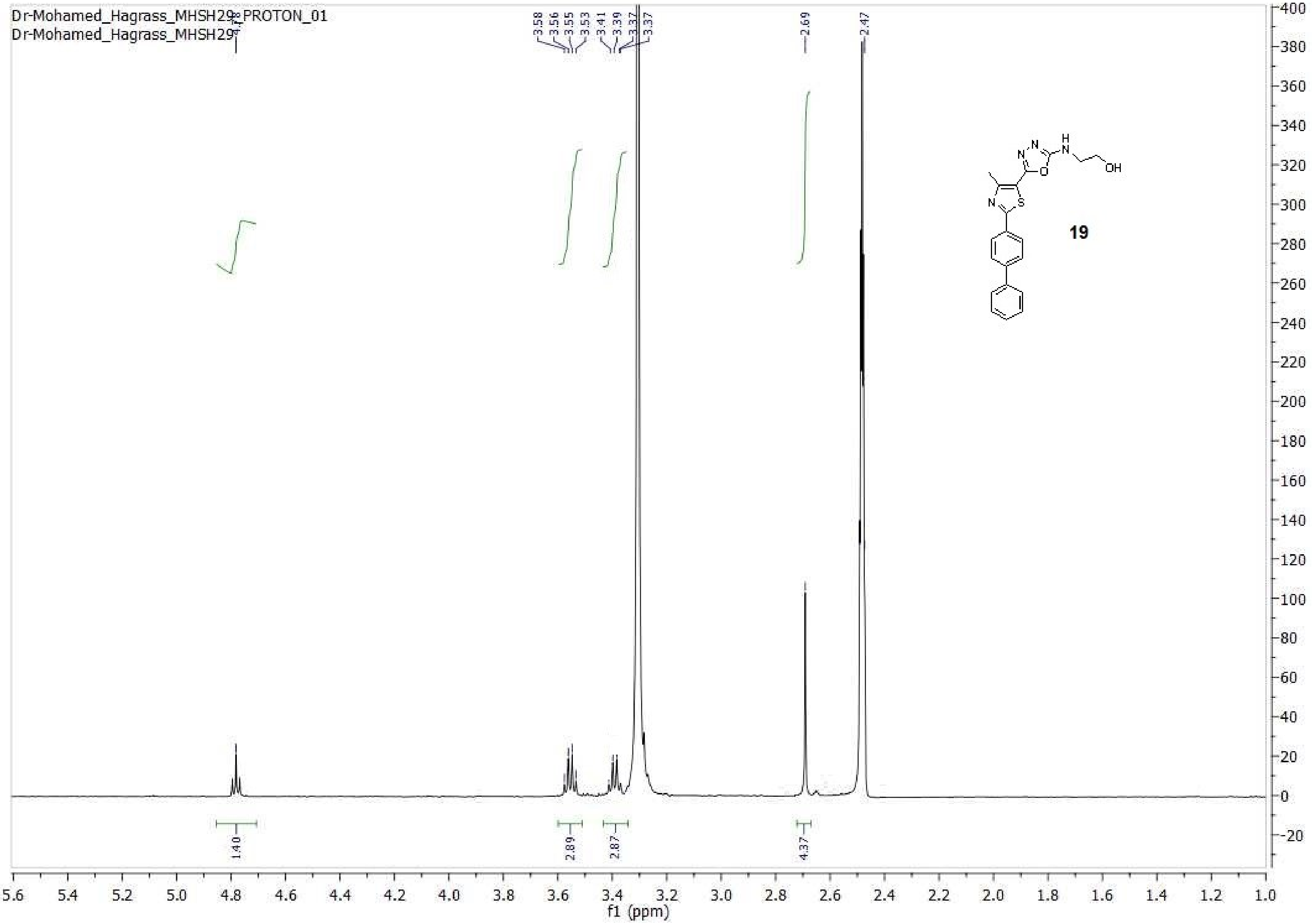
Dr-Mohamed_Hagrass_MHSH29_PROTON_01
Dr-Mohamed_Hagrass_MHSH29
3.58
3.56
3.55
3.53
3.41
3.39
3.37
3.37
2.69
2.47
1.40
2.89
2.87
4.37
f1 (ppm)
N
N
H
N
O
OH
N
S
19

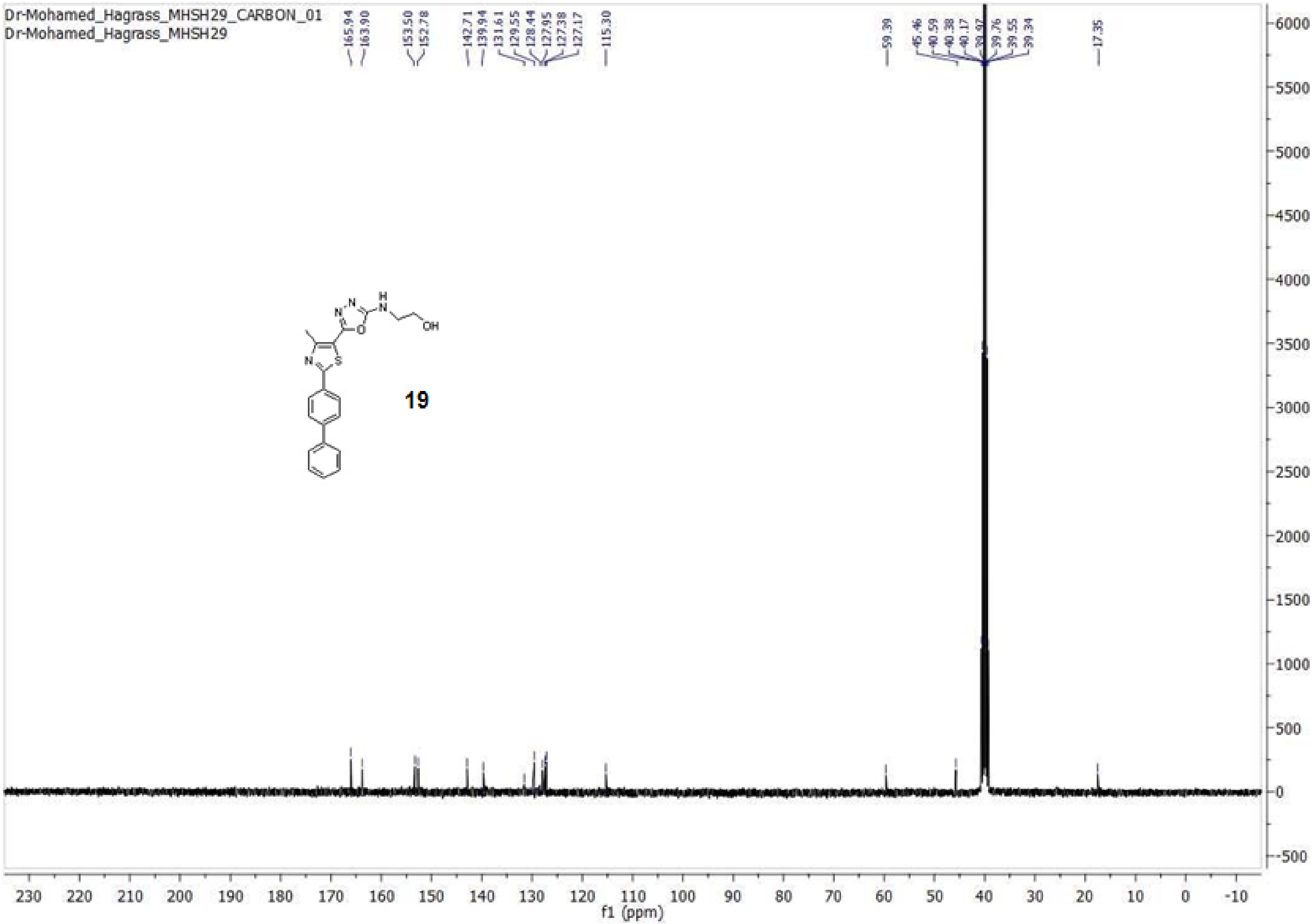

Dr-Mohamed_Hagrass_MHSH29_CARBON_01
Dr-Mohamed_Hagrass_MHSH29
165.94
163.90
153.50
152.78
142.71
139.94
131.61
129.55
128.44
127.95
127.38
127.17
115.30
59.39
45.46
40.59
40.38
40.17
39.97
39.76
39.55
39.34
17.35
f1 (ppm)

19

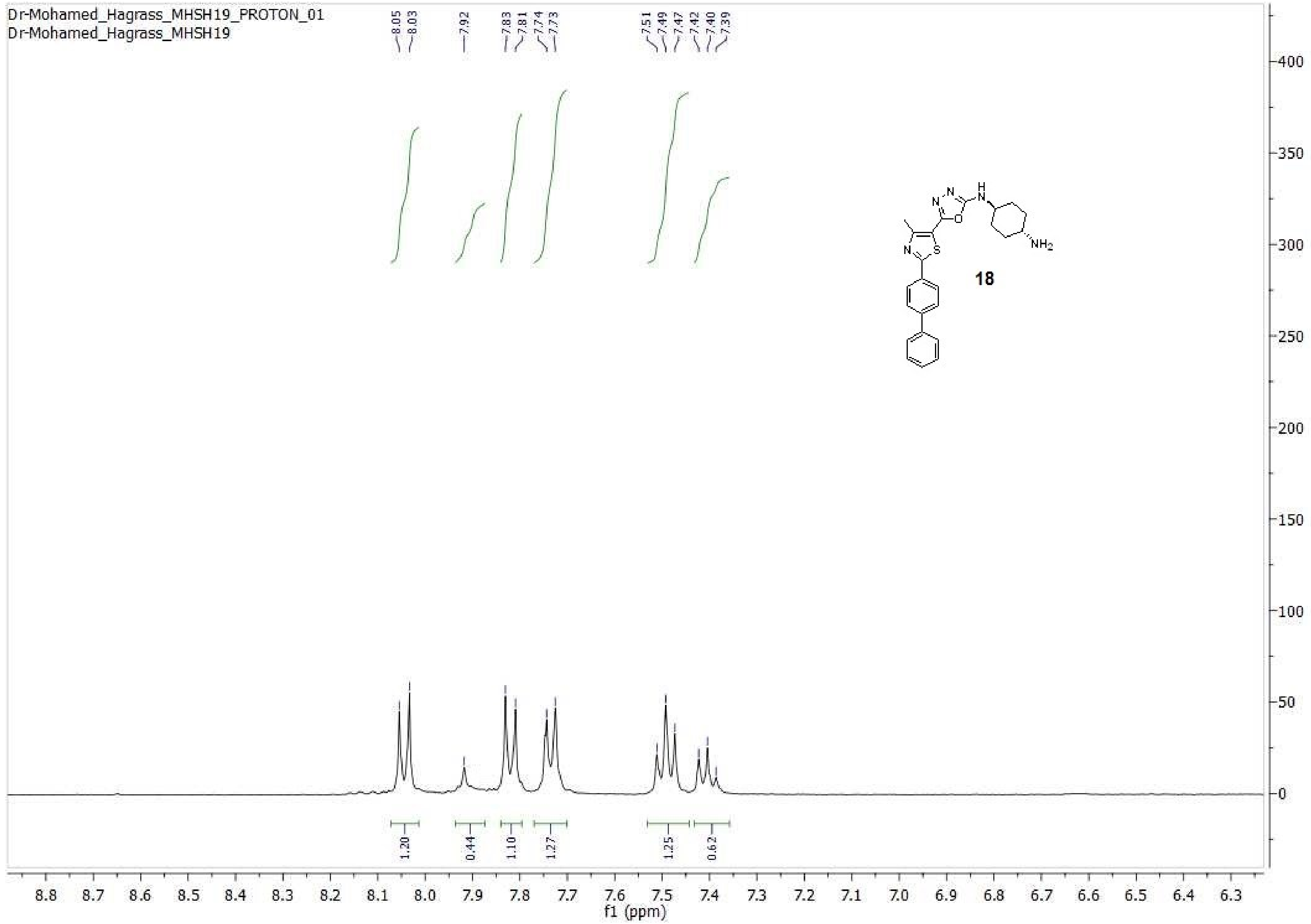
Dr-Mohamed_Hagrass_MHSH19_PROTON_01
Dr-Mohamed_Hagrass_MHSH19
8.05
8.03
7.92
7.83
7.81
7.74
7.73
7.51
7.49
7.47
7.42
7.40
7.39
1.20
0.44
1.10
1.27
1.25
0.62
f1 (ppm)
NH2
18

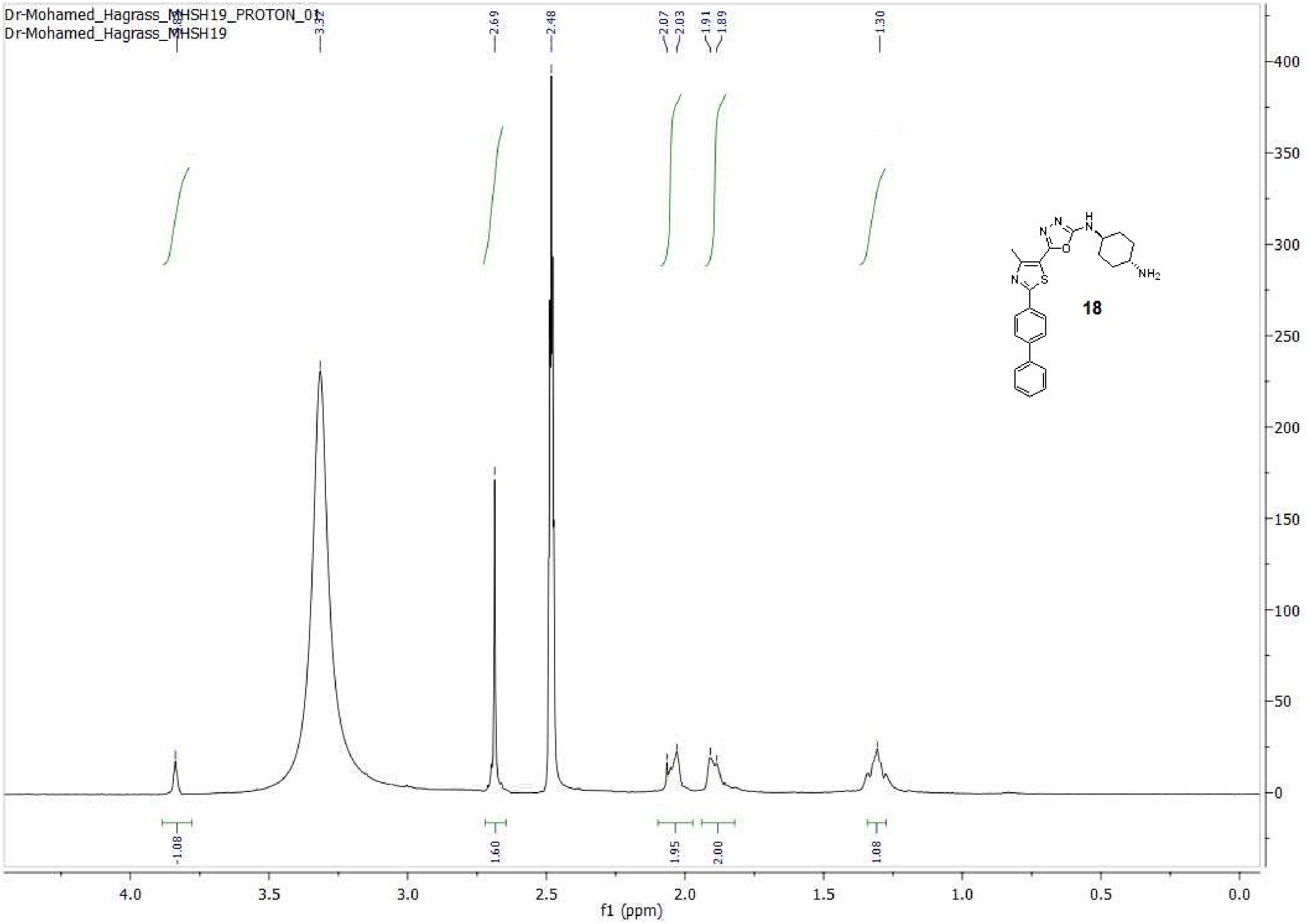
Dr-Mohamed_Hagrass_MHSH19_PROTON_01
Dr-Mohamed_Hagrass_MHSH19
3.83
3.32
2.69
2.48
2.07
2.03
1.91
1.89
1.30
1.08
1.60
1.95
2.00
1.08
18
f1 (ppm)

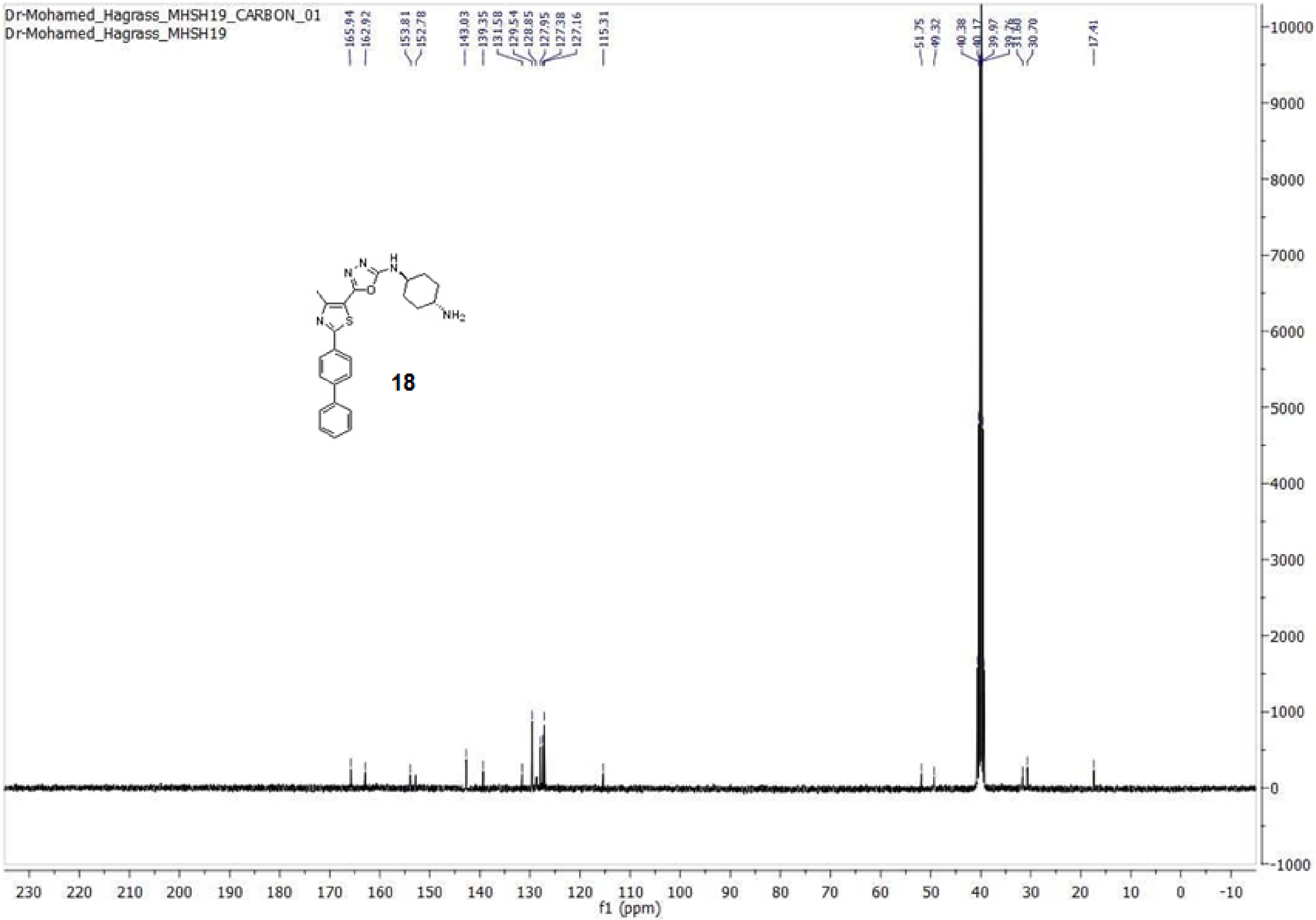

Dr-Mohamed_Hagrass_MHSH19_CARBON_01
Dr-Mohamed_Hagrass_MHSH19
165.94
162.92
153.81
152.78
143.03
139.35
131.58
129.54
128.85
127.95
127.38
127.16
115.31
51.75
49.32
40.38
40.17
39.97
30.70
17.41
18
f1 (ppm)

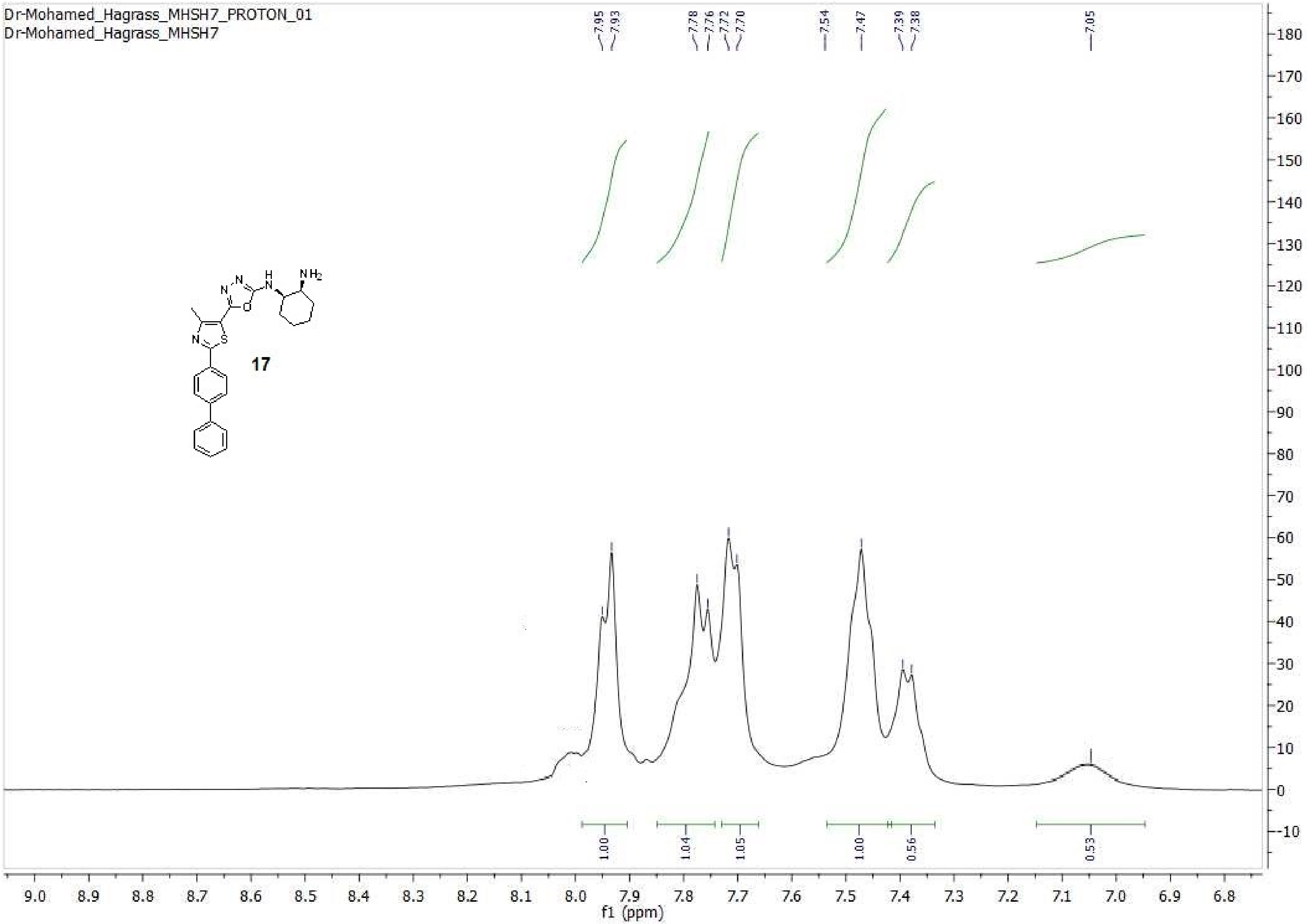

Dr-Mohamed_Hagrass_MHSH7_PROTON_01
Dr-Mohamed_Hagrass_MHSH7
7.95
7.93
7.78
7.76
7.72
7.70
7.54
7.47
7.39
7.38
7.05
17
1.00
1.04
1.05
1.00
0.56
0.53
f1 (ppm)

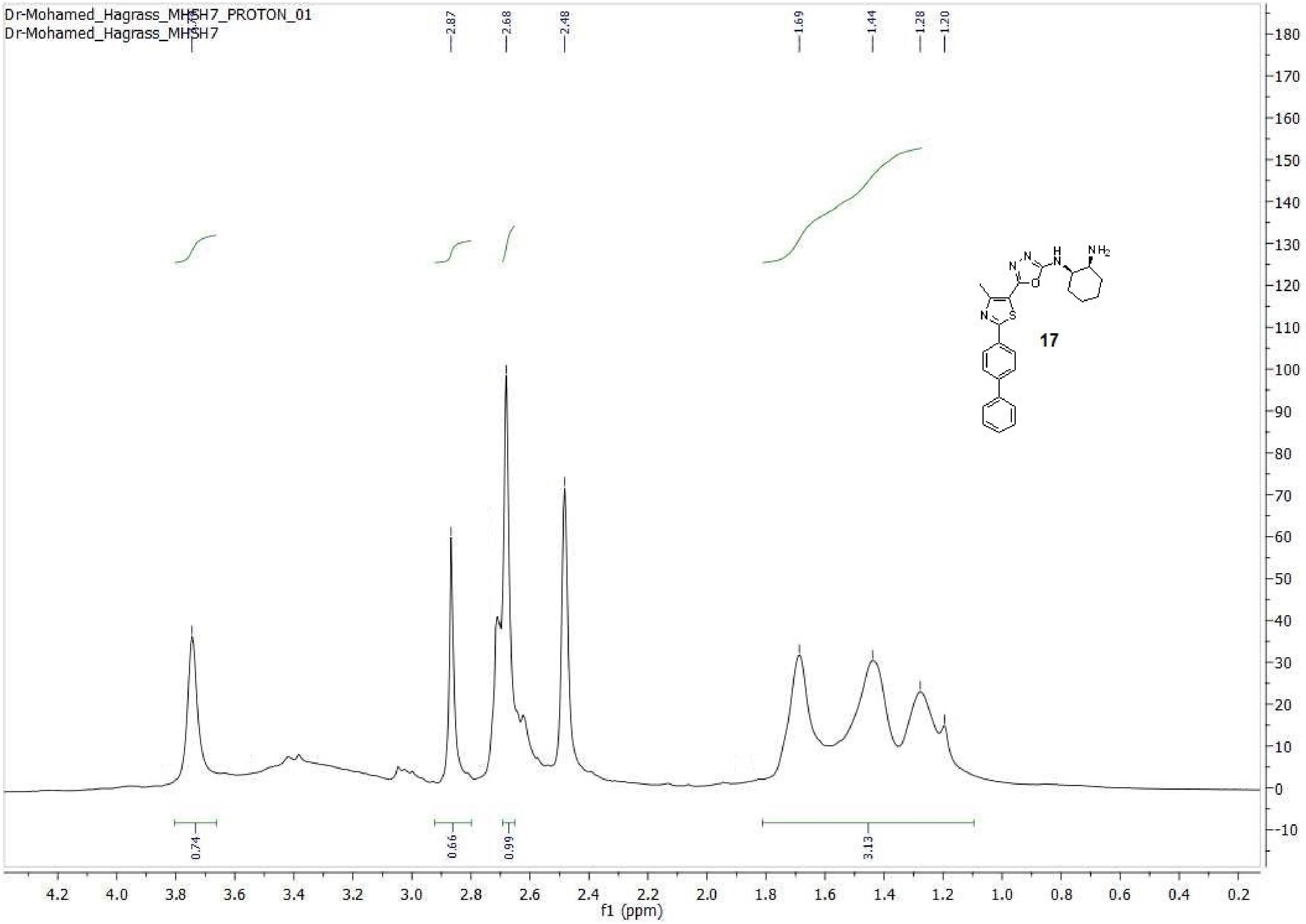
Dr-Mohamed_Hagrass_MHSH7_PROTON_01
Dr-Mohamed_Hagrass_MHSH7
2.87
2.68
2.48
1.69
1.44
1.28
1.20
0.74
0.66
0.99
3.13
17
f1 (ppm)

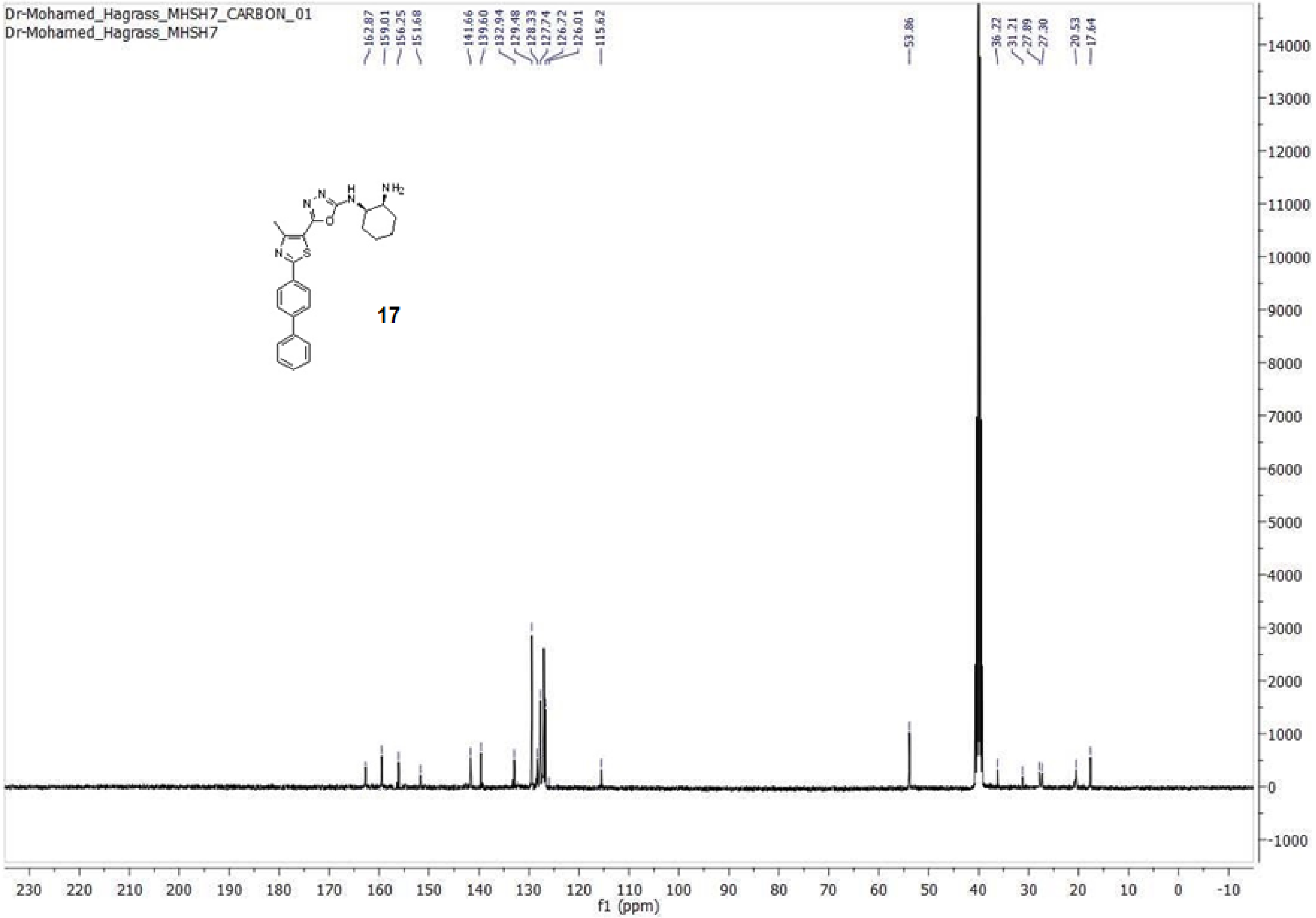
Dr-Mohamed_Hagrass_MHSH7_CARBON_01
Dr-Mohamed_Hagrass_MHSH7
162.87
159.01
156.25
151.68
141.66
139.60
132.94
129.48
128.33
127.74
126.72
126.01
115.62
53.86
36.22
31.21
27.89
27.30
20.53
17.64
17
f1 (ppm)

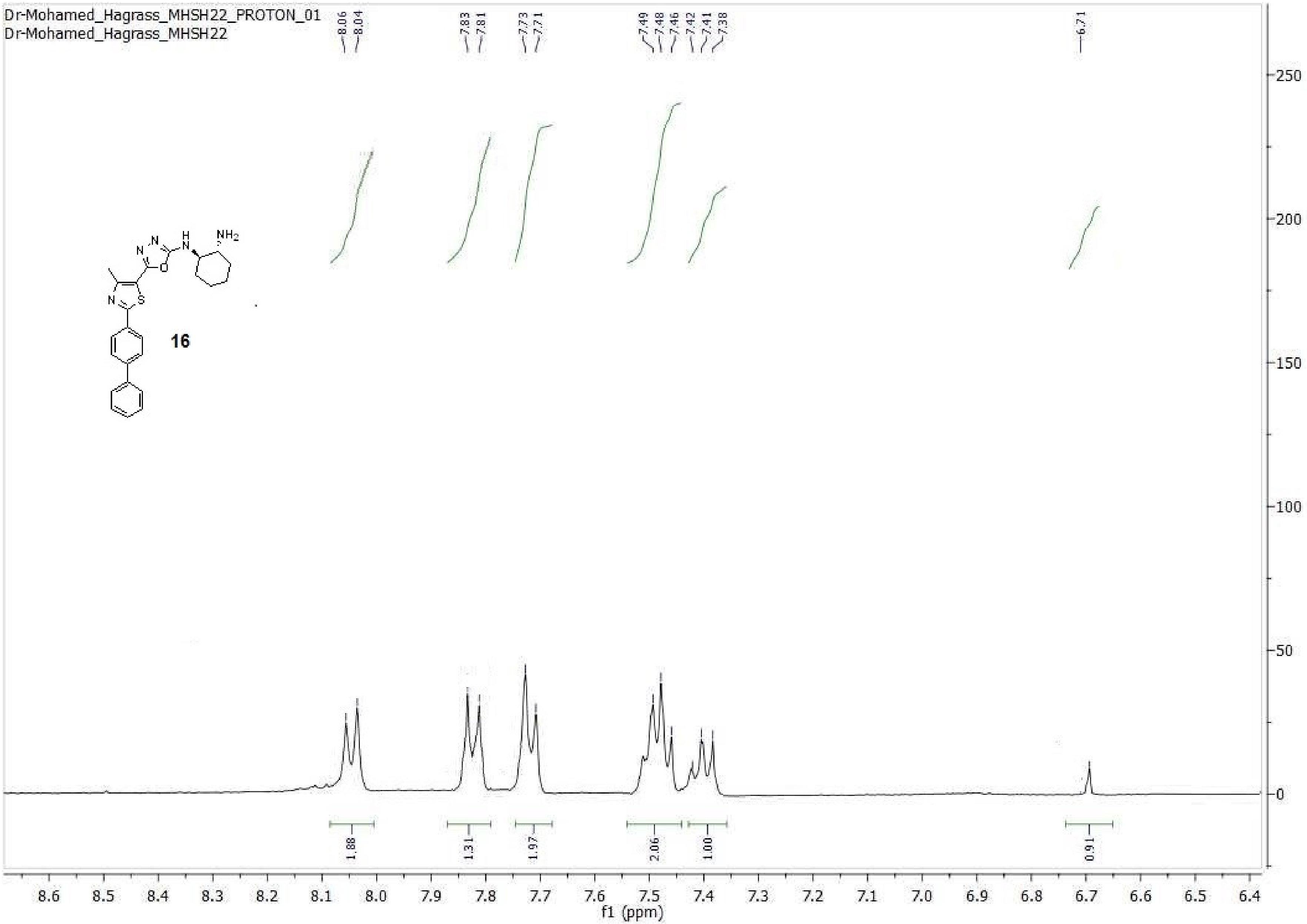

Dr-Mohamed_Hagrass_MHSH22_PROTON_01
Dr-Mohamed_Hagrass_MHSH22
8.06
8.04
7.83
7.81
7.73
7.71
7.49
7.48
7.46
7.42
7.41
7.38
6.71
16
1.88
1.31
1.97
2.06
1.00
0.91
f1 (ppm)

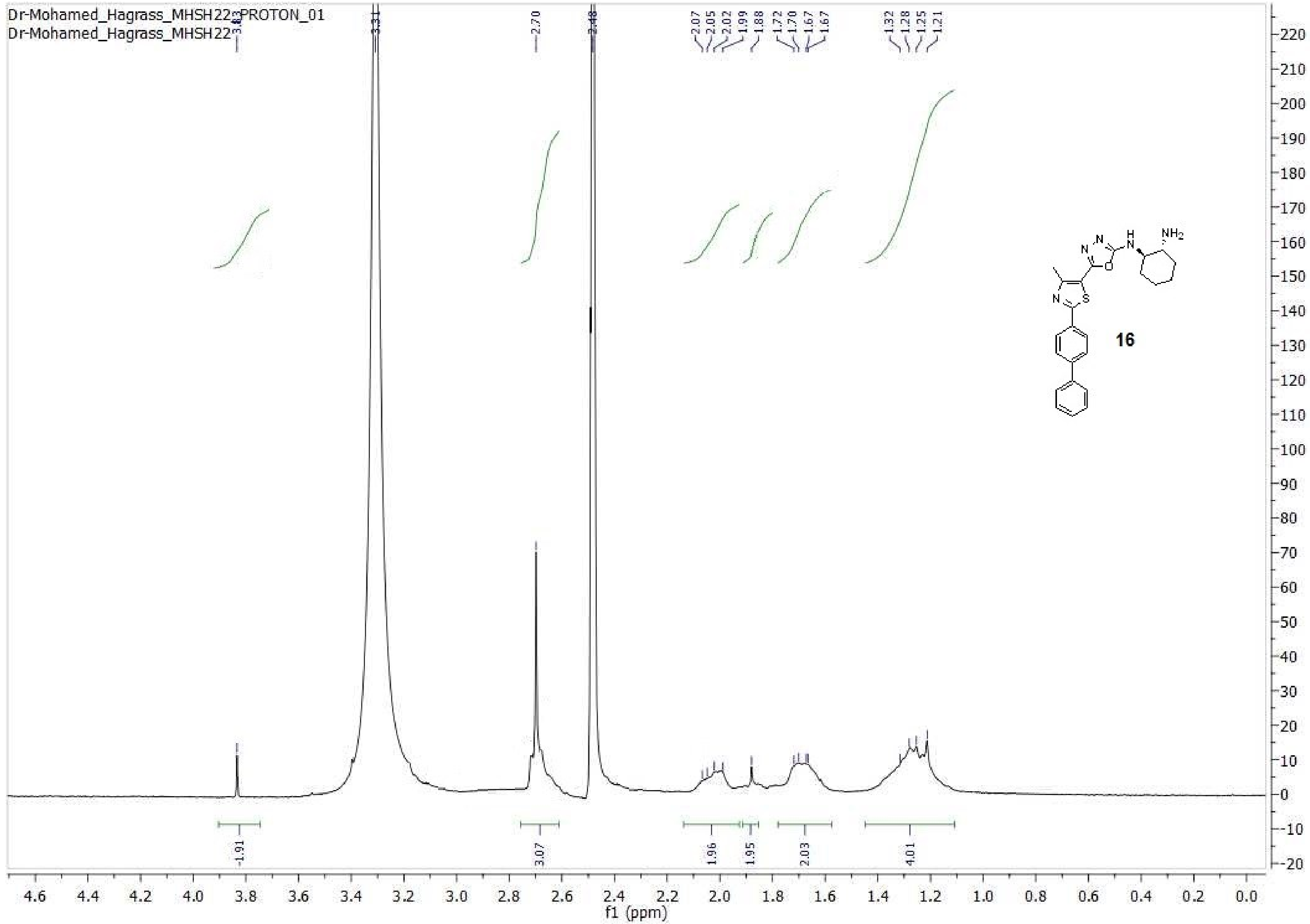
Dr-Mohamed_Hagrass_MHSH22_PROTON_01
Dr-Mohamed_Hagrass_MHSH22
16
f1 (ppm)

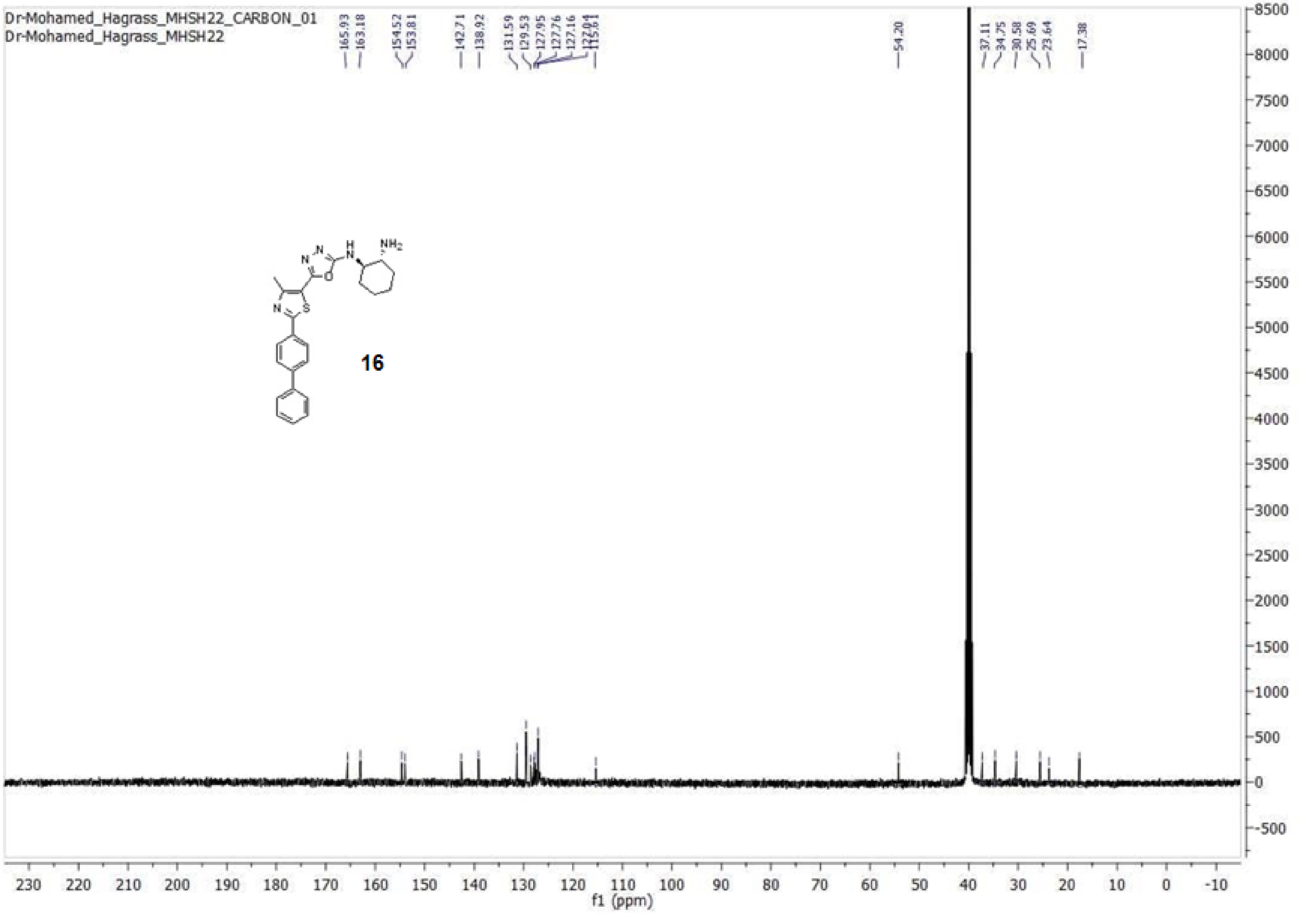
Dr-Mohamed_Hagrass_MHSH22_CARBON_01
Dr-Mohamed_Hagrass_MHSH22
165.93
163.18
154.52
153.81
142.71
138.92
131.59
129.53
127.95
127.76
127.16
54.20
37.11
34.75
30.58
25.69
23.64
17.38
16
f1 (ppm)

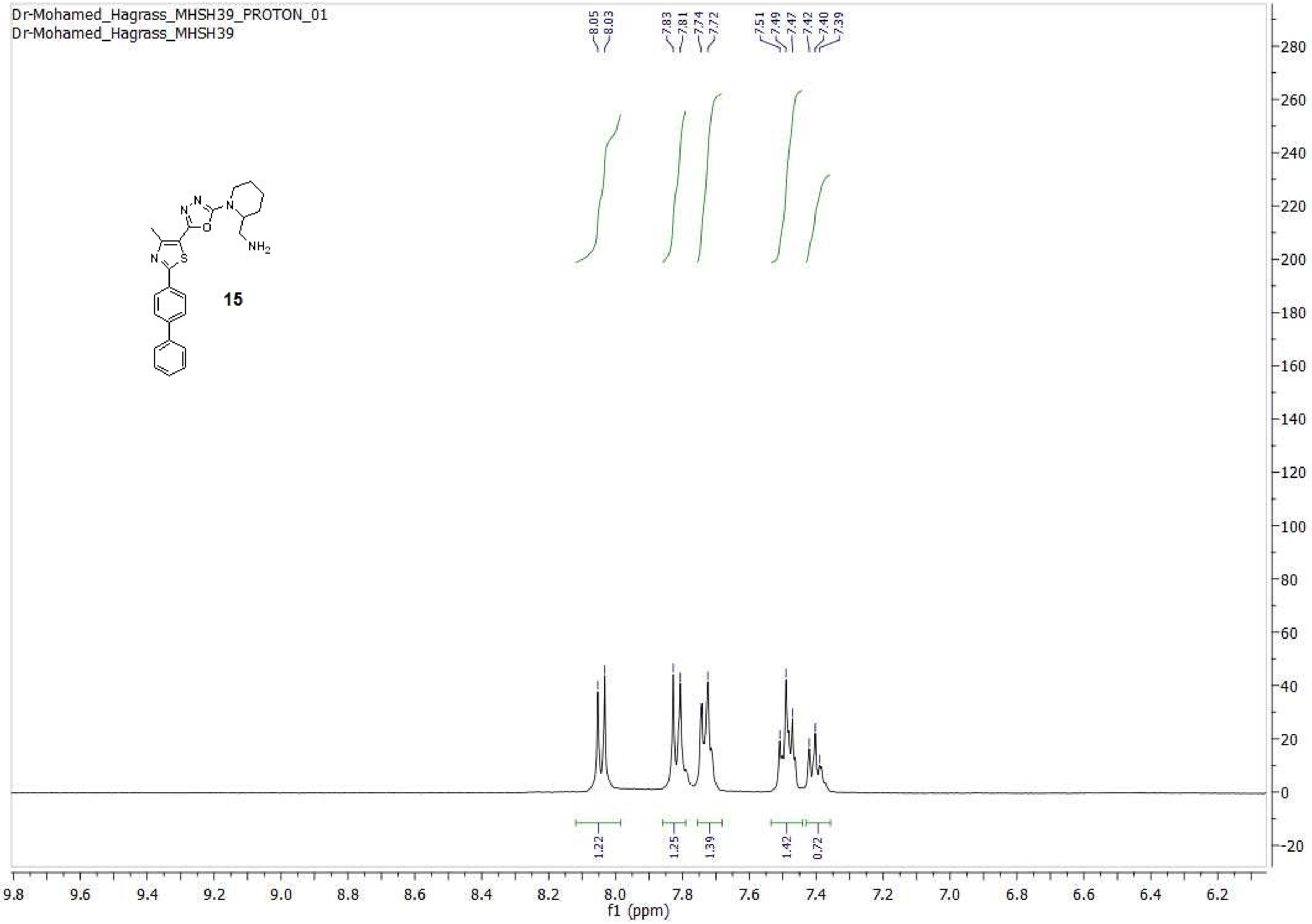
Dr-Mohamed_Hagrass_MHSH39_PROTON_01
Dr-Mohamed_Hagrass_MHSH39
8.05
8.03
7.83
7.81
7.74
7.72
7.51
7.49
7.47
7.42
7.40
7.39
15
1.22
1.25
1.39
1.42
0.72
f1 (ppm)

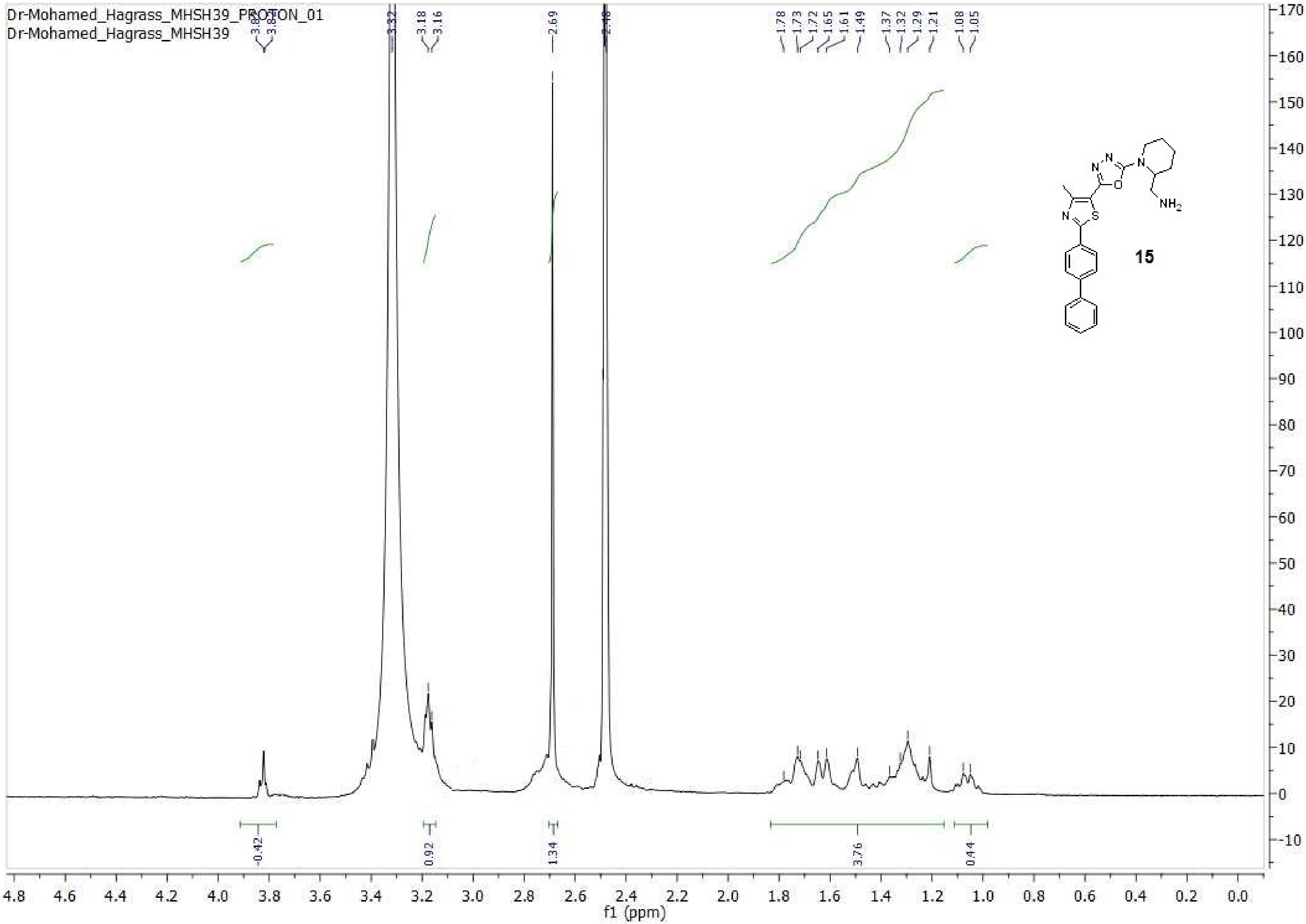
Dr-Mohamed_Hagrass_MHSH39_PROTON_01
Dr-Mohamed_Hagrass_MHSH39
3.32
3.18
3.16
2.69
1.78
1.73
1.72
1.65
1.61
1.49
1.37
1.32
1.29
1.21
1.08
1.05
0.42
0.92
1.34
3.76
0.44
N
N
O
N
S
N
NH_2
15
f1 (ppm)

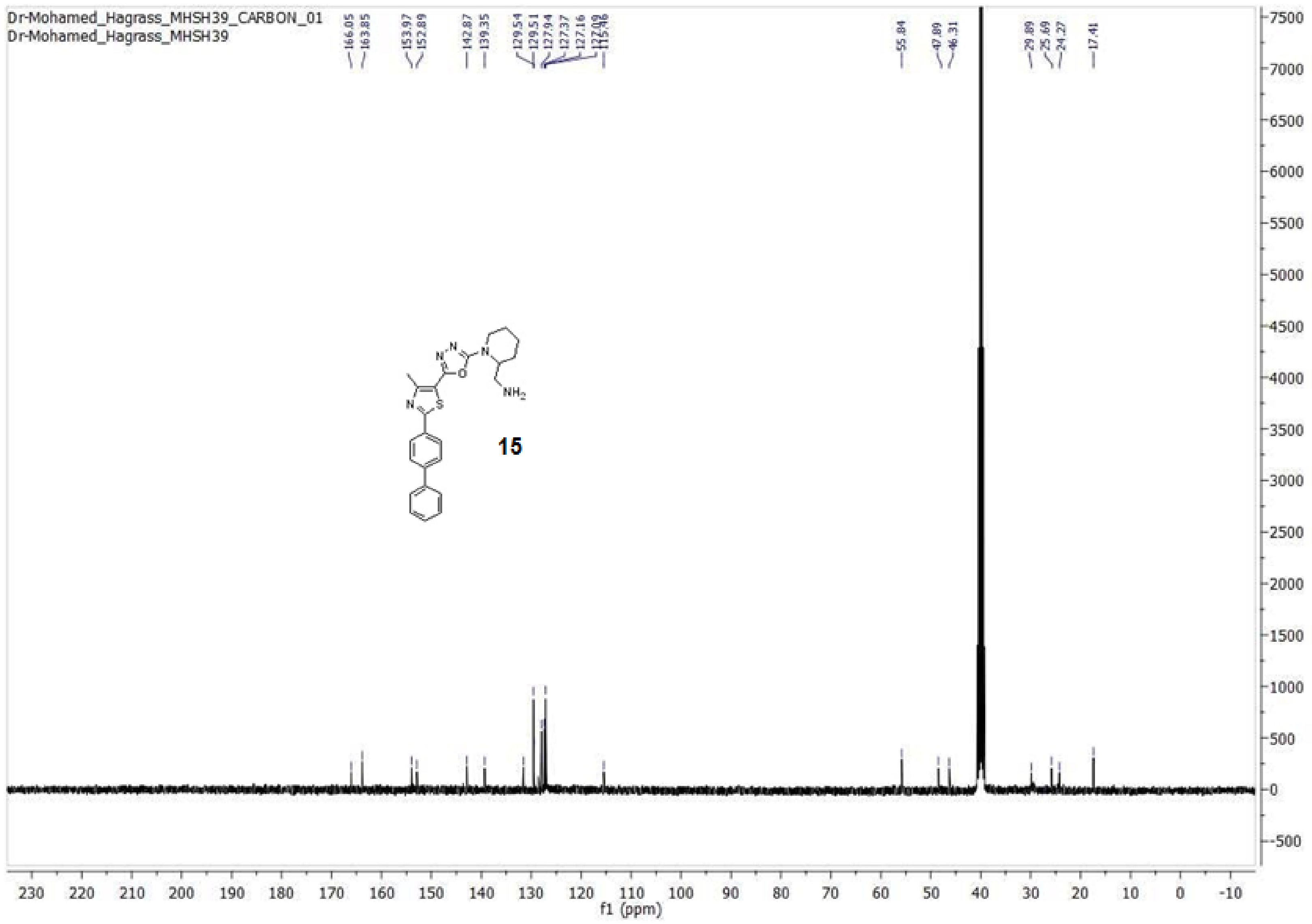
Dr-Mohamed_Hagrass_MHSH39_CARBON_01
Dr-Mohamed_Hagrass_MHSH39
166.05
163.85
153.97
152.89
142.87
139.35
129.54
129.51
127.94
127.37
127.16
55.84
47.89
46.31
29.89
25.69
24.27
17.41
NH2
15
f1 (ppm)

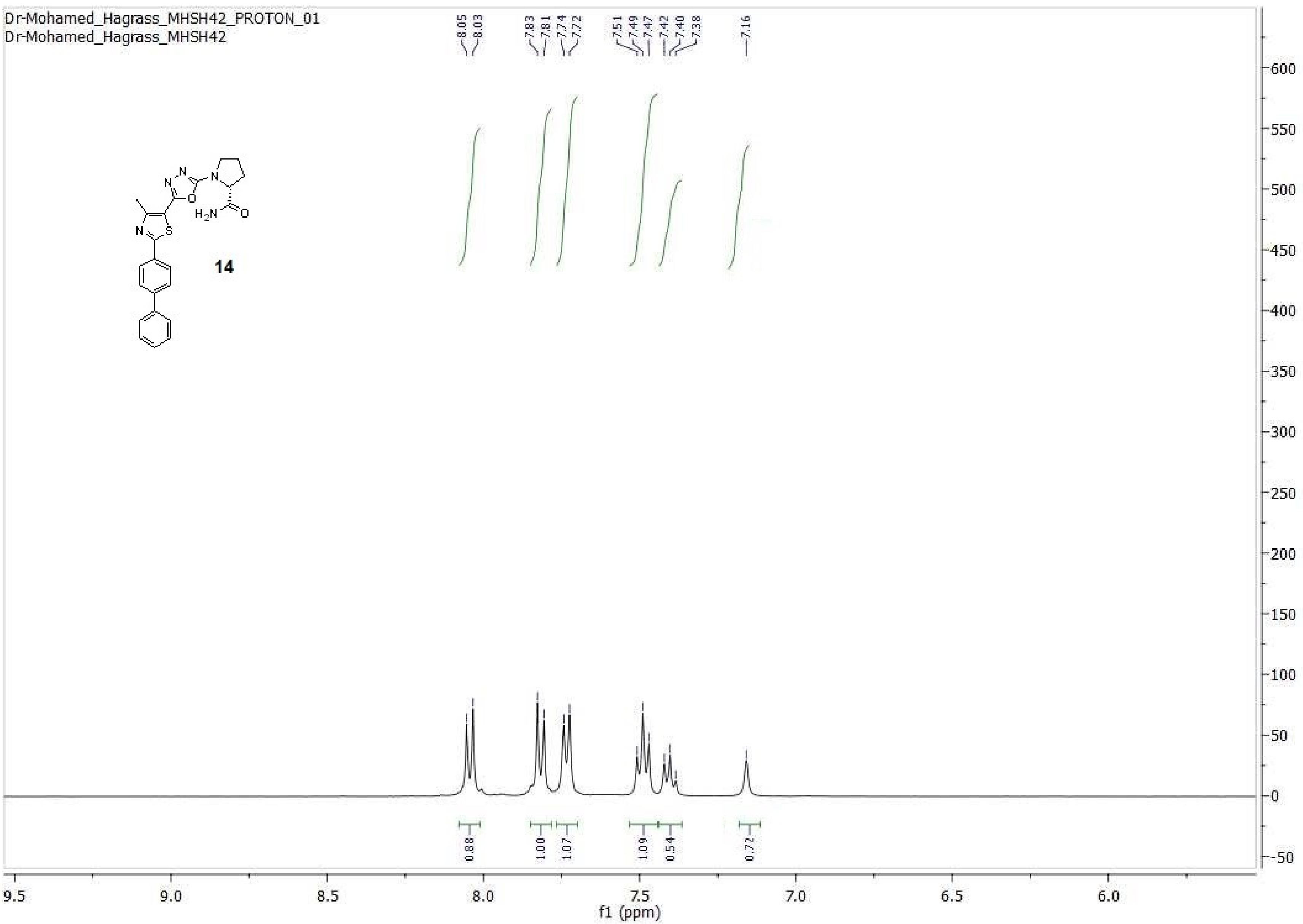
Dr-Mohamed_Hagrass_MHSH42_PROTON_01
Dr-Mohamed_Hagrass_MHSH42
8.05
8.03
7.83
7.81
7.74
7.72
7.51
7.49
7.47
7.42
7.40
7.38
7.16
14
0.88
1.00
1.07
1.09
0.54
0.72
f1 (ppm)

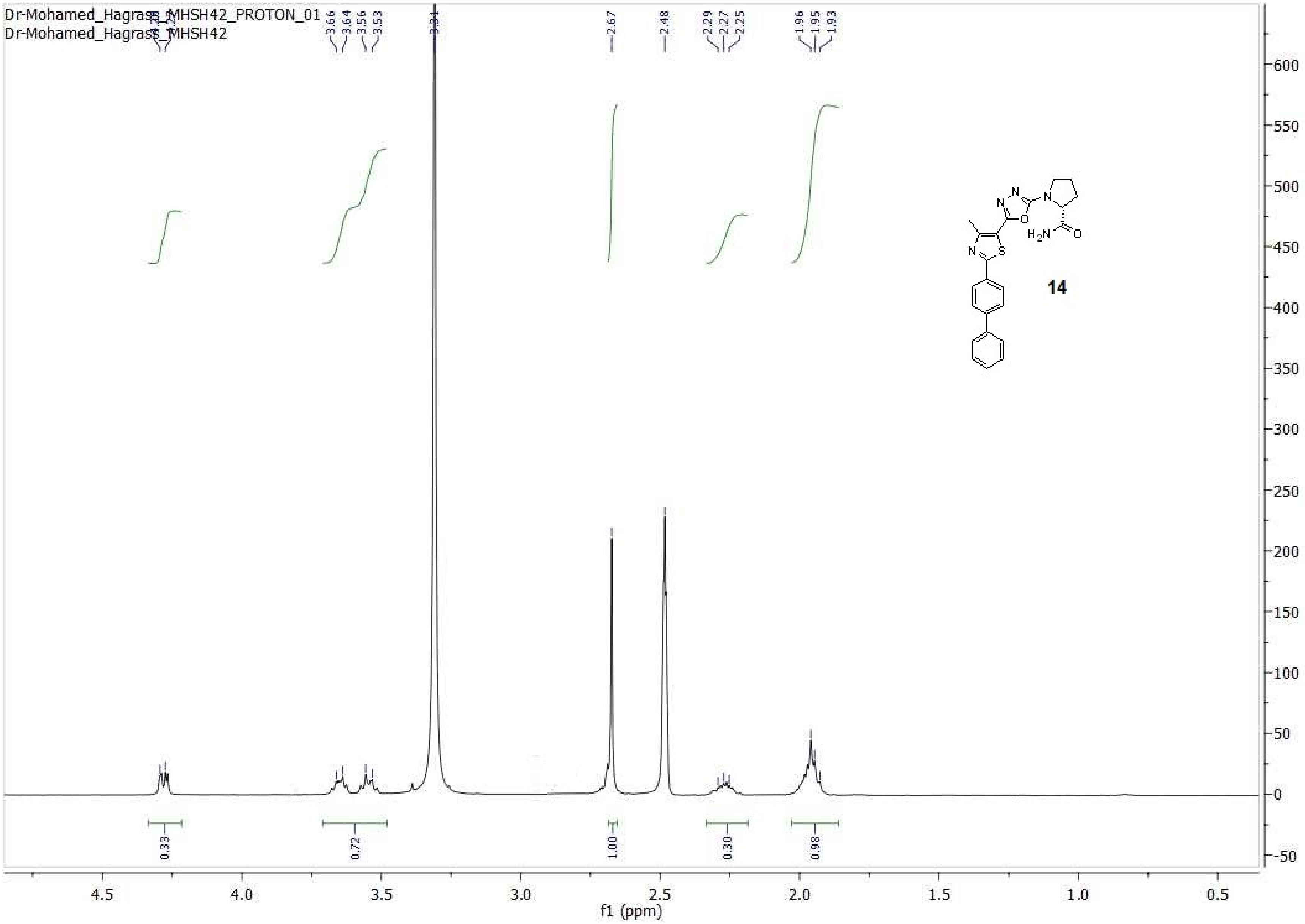
Dr-Mohamed_Hagrass_MHSH42_PROTON_01
Dr-Mohamed_Hagrass_MHSH42
3.66
3.64
3.56
3.53
3.31
2.67
2.48
2.29
2.27
2.25
1.96
1.95
1.93
14
0.33
0.72
1.00
0.30
0.98
f1 (ppm)

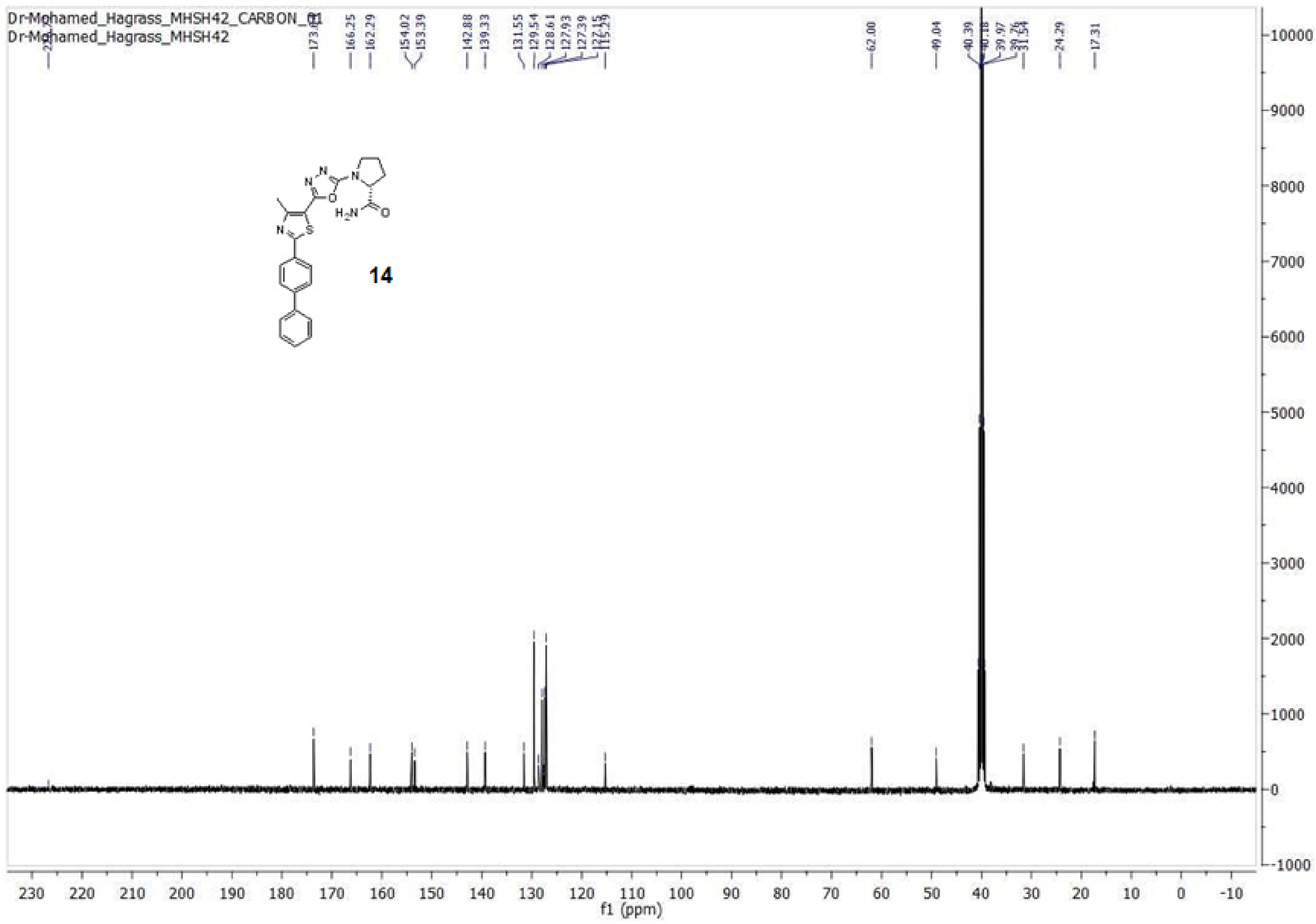
Dr-Mohamed_Hagrass_MHSH42_CARBON_01
Dr-Mohamed_Hagrass_MHSH42
14
f1 (ppm)

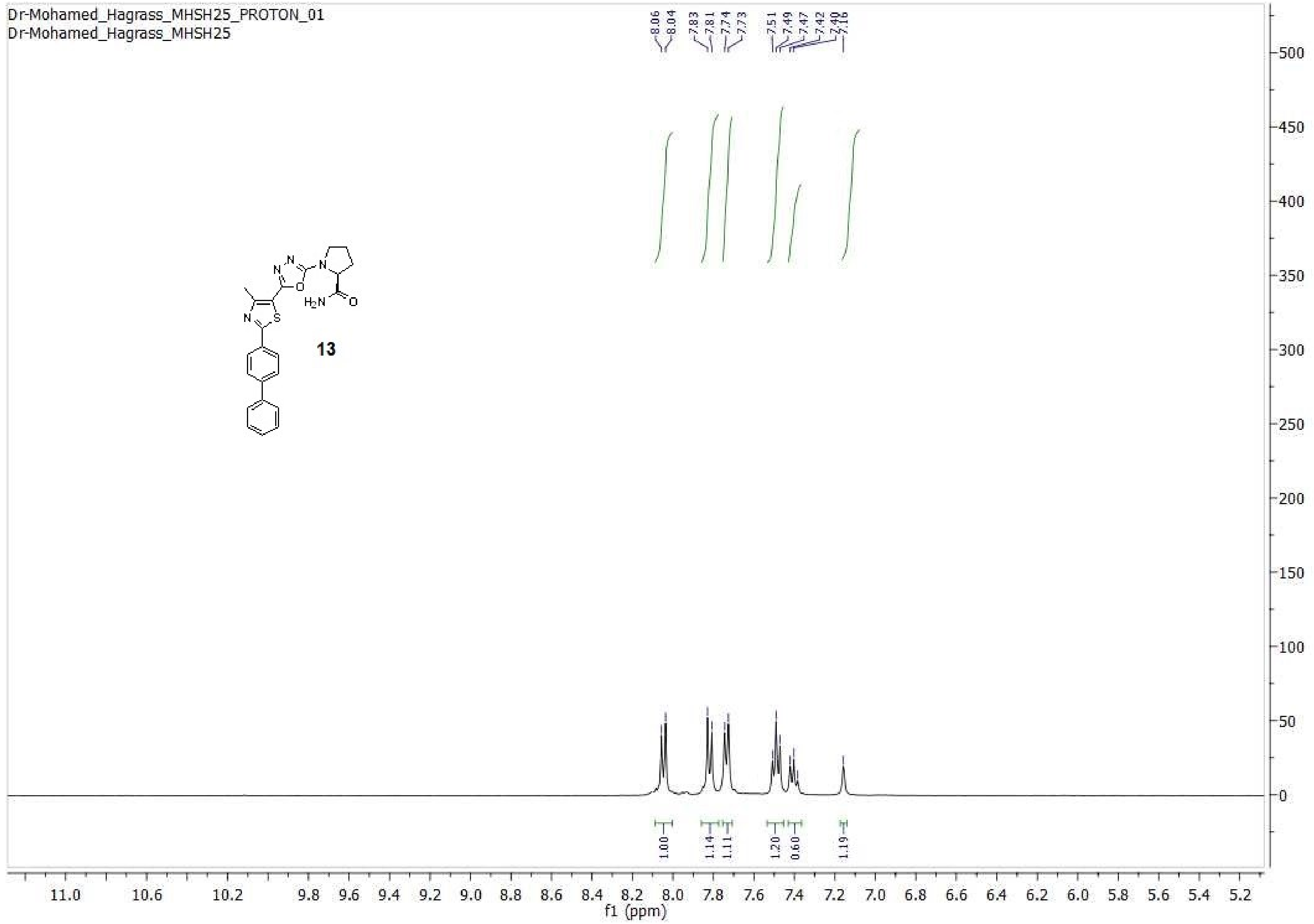
Dr-Mohamed_Hagrass_MHSH25_PROTON_01
Dr-Mohamed_Hagrass_MHSH25
8.06
8.04
7.83
7.81
7.74
7.73
7.51
7.49
7.47
7.42
7.40
7.16
13
1.00
1.14
1.11
1.20
0.60
1.19
f1 (ppm)

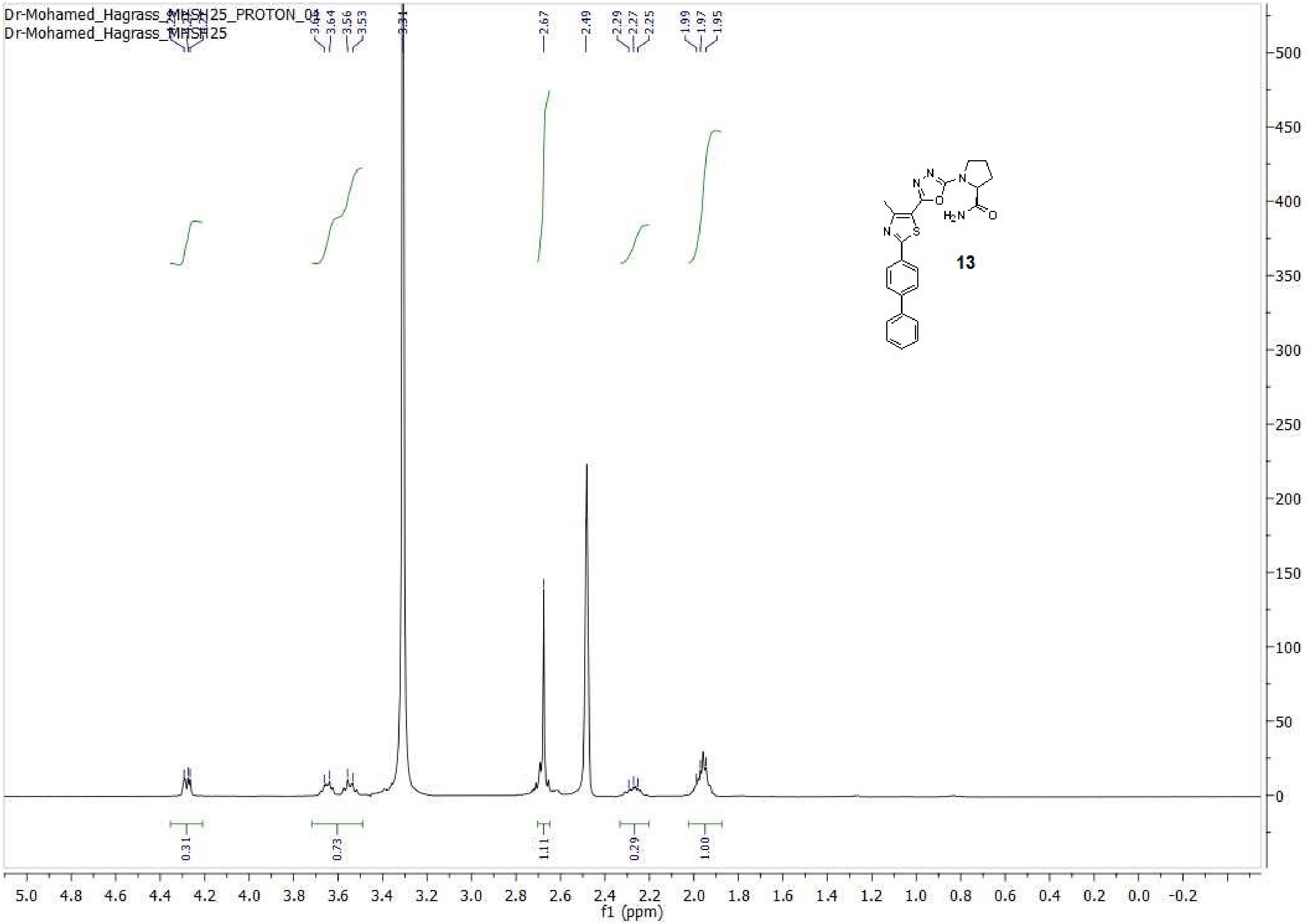
Dr-Mohamed_Hagrass_MHSH25_PROTON_01
Dr-Mohamed_Hagrass_MHSH25
3.64
3.56
3.53
3.31
2.67
2.49
2.29
2.27
2.25
1.99
1.97
1.95
0.31
0.73
1.11
0.29
1.00
f1 (ppm)
13

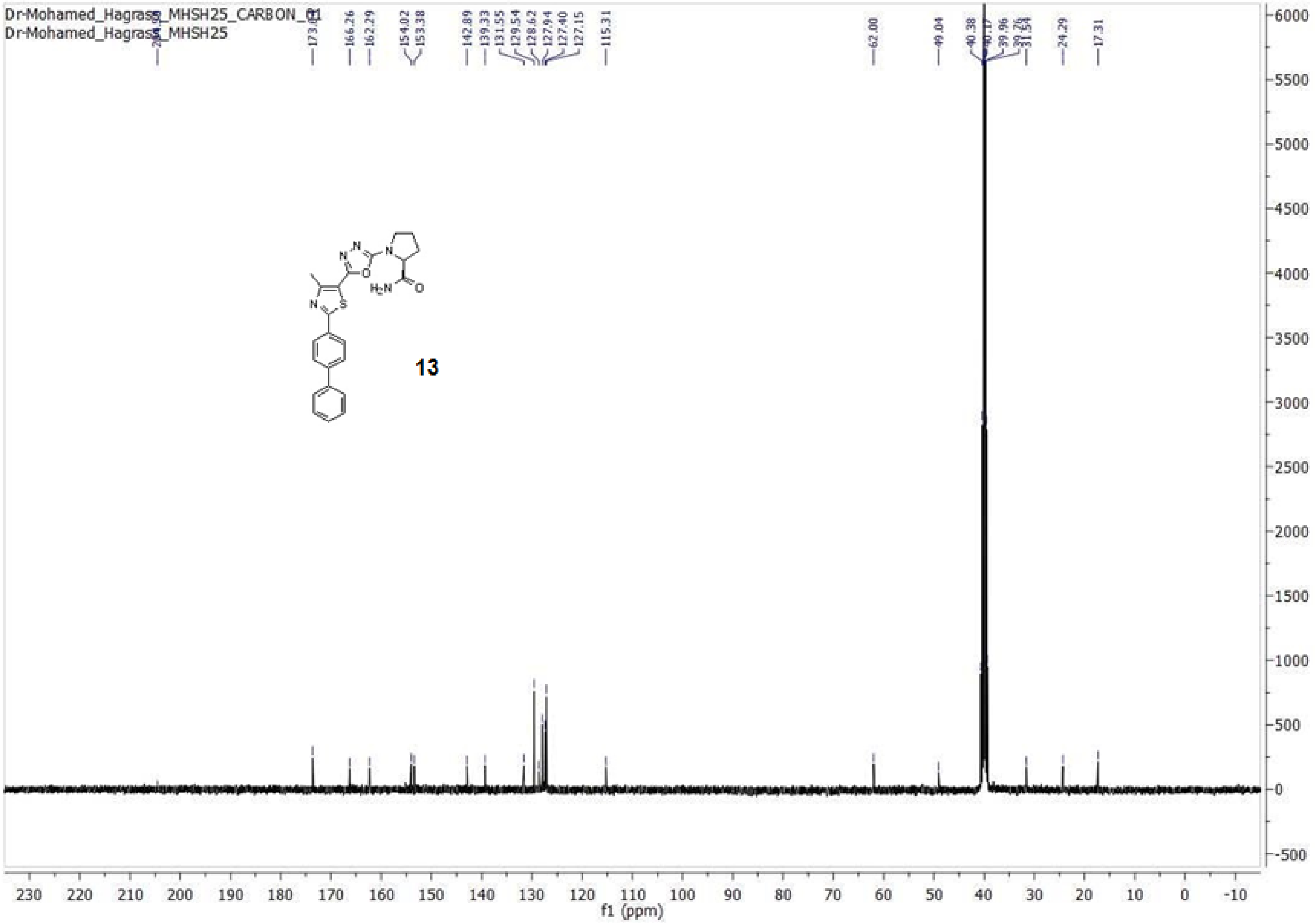
Dr-Mohamed_Hagrass_MHSH25_CARBON_01
Dr-Mohamed_Hagrass_MHSH25
173.01
166.26
162.29
154.02
153.38
142.89
139.33
131.55
129.54
128.62
127.94
127.40
127.15
115.31
62.00
49.04
40.38
40.17
39.96
31.54
24.29
17.31
13
f1 (ppm)

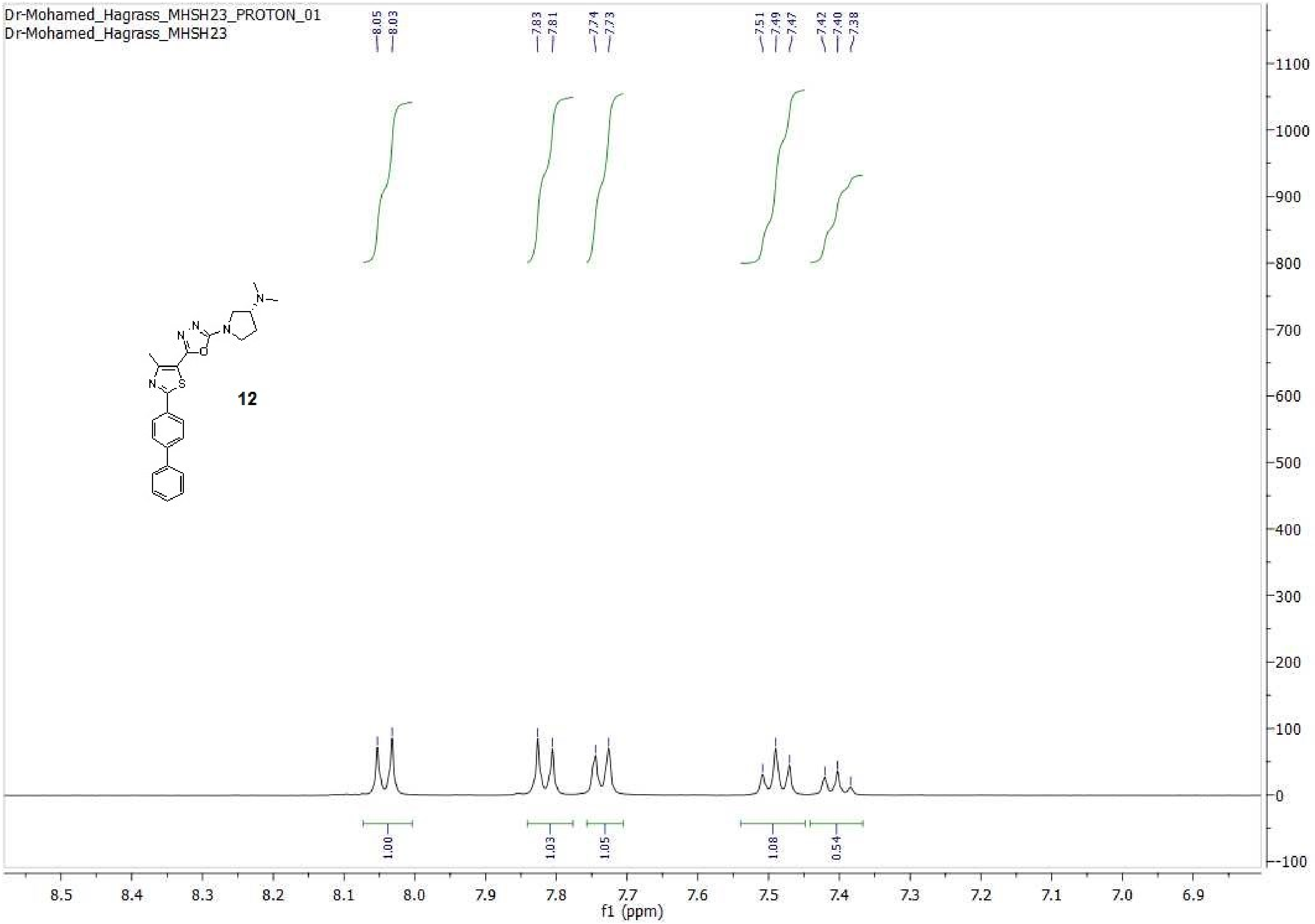

Dr-Mohamed_Hagrass_MHSH23_PROTON_01
Dr-Mohamed_Hagrass_MHSH23
8.05
8.03
7.83
7.81
7.74
7.73
7.51
7.49
7.47
7.42
7.40
7.38
12
1.00
1.03
1.05
1.08
0.54
f1 (ppm)

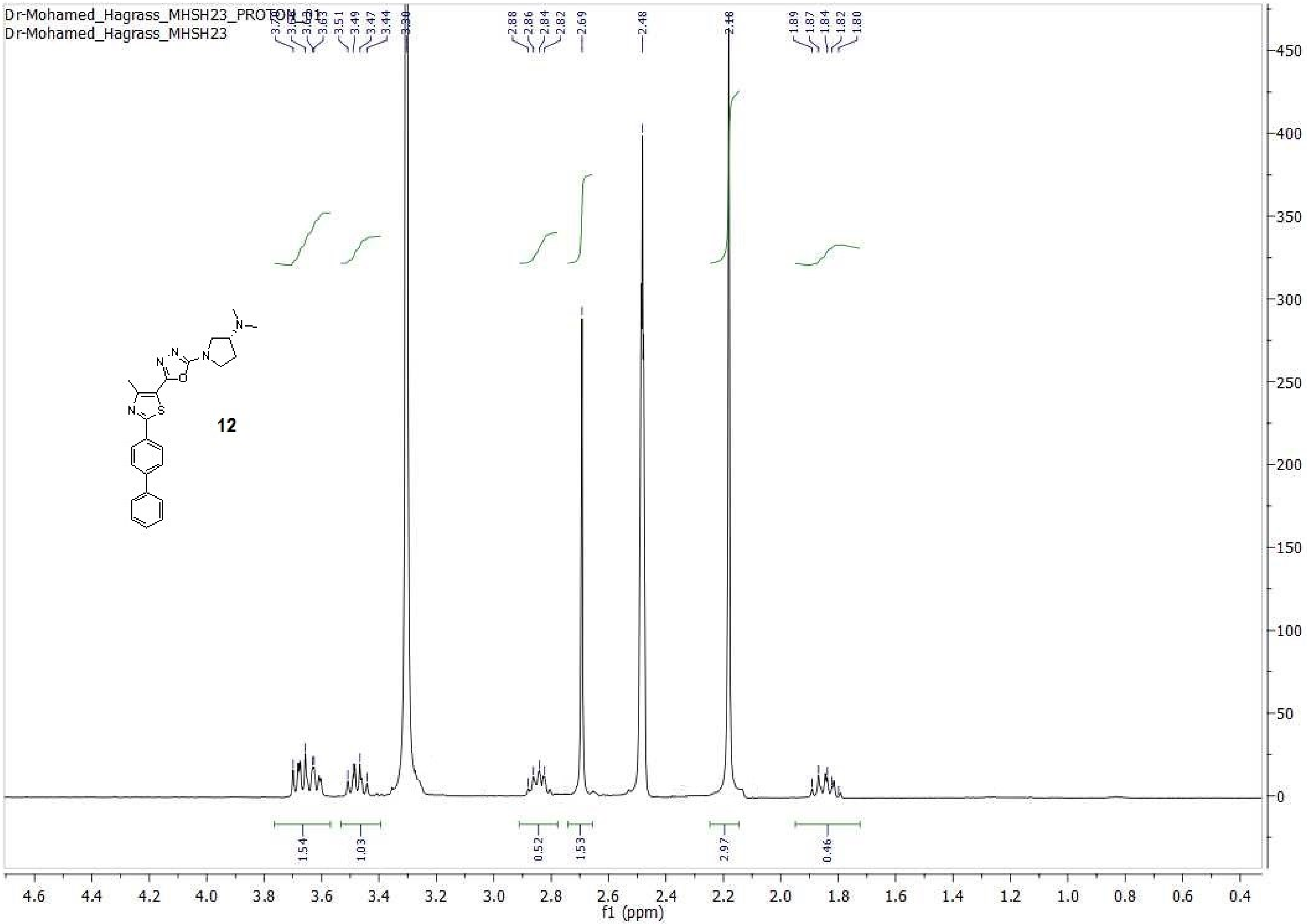

Dr-Mohamed_Hagrass_MHSH23_PROTON_01
Dr-Mohamed_Hagrass_MHSH23
12
f1 (ppm)
1.54
1.03
0.52
1.53
2.97
0.46

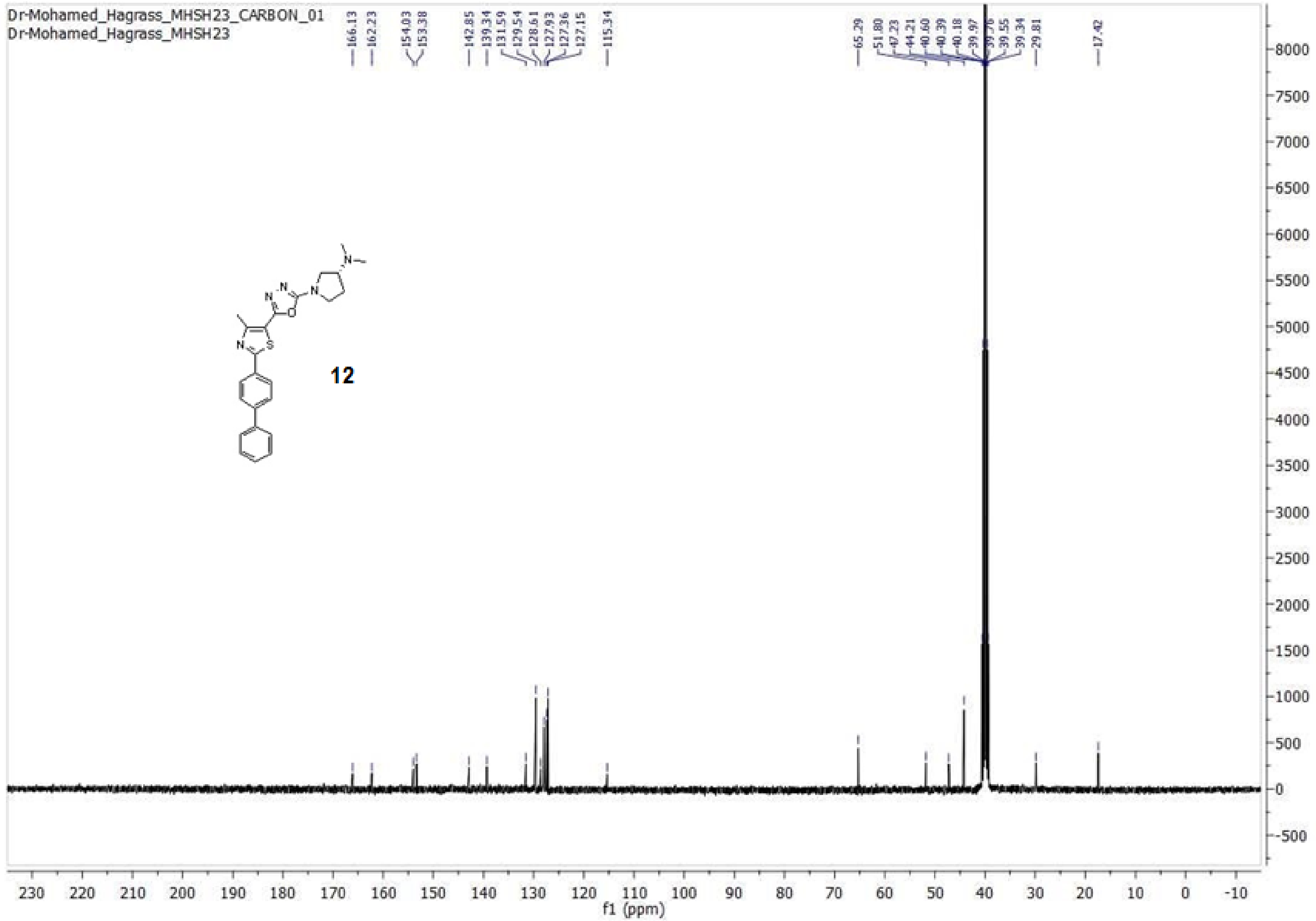
Dr-Mohamed_Hagrass_MHSH23_CARBON_01
Dr-Mohamed_Hagrass_MHSH23
166.13
162.23
154.03
153.38
142.85
139.34
131.59
129.54
128.61
127.93
127.36
127.15
115.34
65.29
51.80
47.23
44.21
40.60
40.39
40.18
39.97
39.55
39.34
29.81
17.42
12
f1 (ppm)

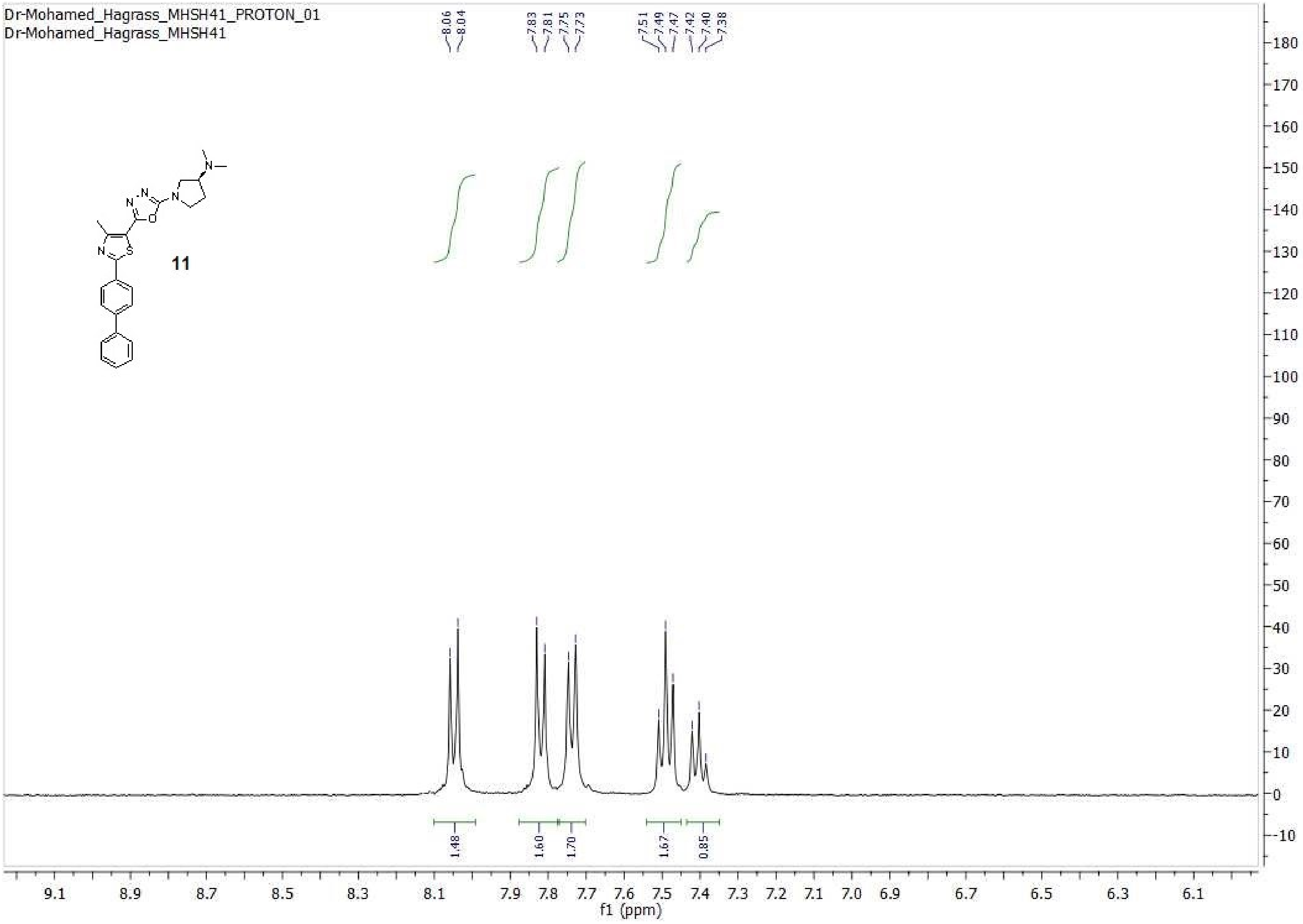
Dr-Mohamed_Hagrass_MHSH41_PROTON_01
Dr-Mohamed_Hagrass_MHSH41
8.06
8.04
7.83
7.81
7.75
7.73
7.51
7.49
7.47
7.42
7.40
7.38
11
1.48
1.60
1.70
1.67
0.85
f1 (ppm)

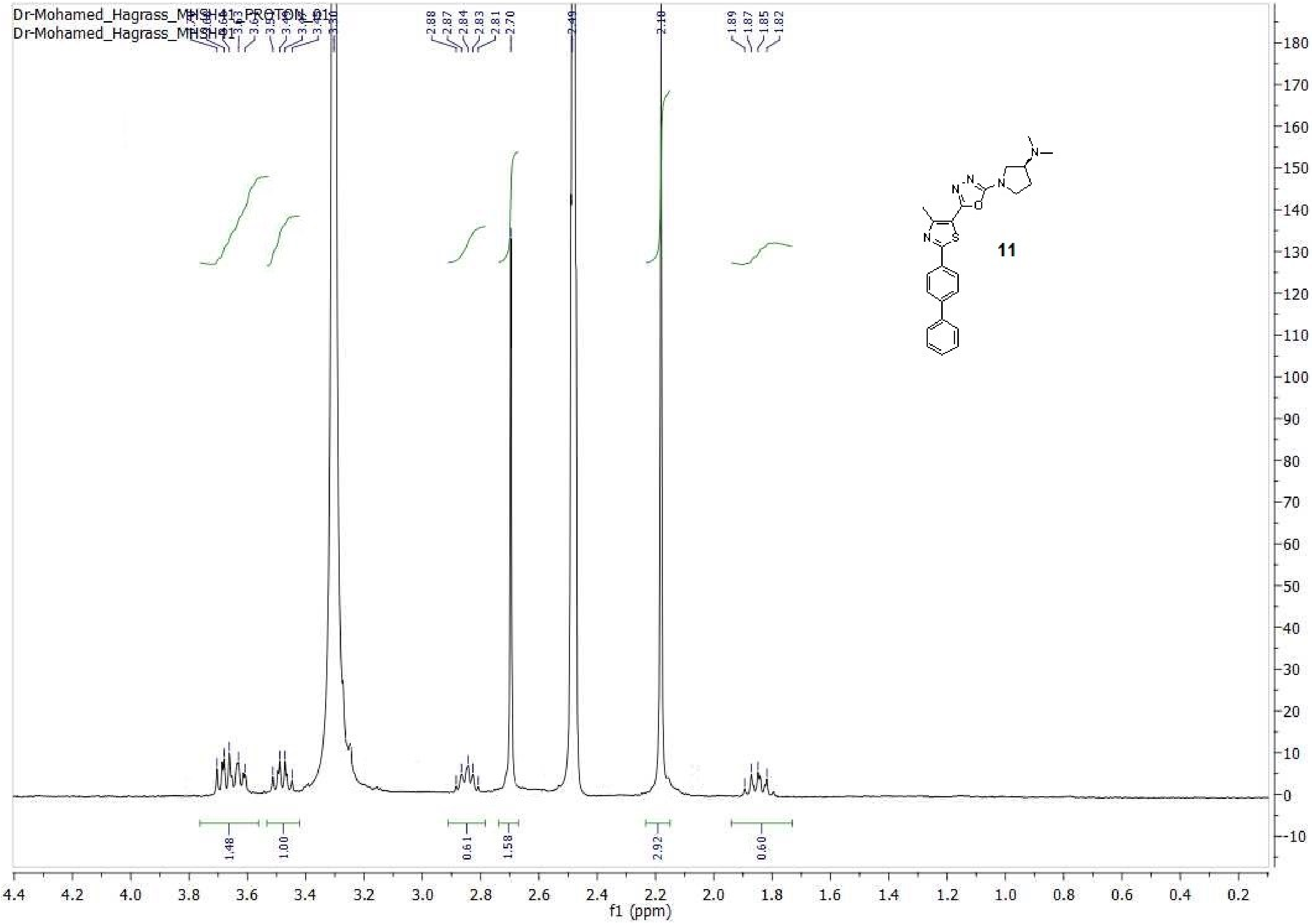
Dr-Mohamed_Hagrass_MHSH41_PROTON_01
Dr-Mohamed_Hagrass_MHSH41
2.88
2.87
2.84
2.83
2.81
2.70
2.49
2.18
1.89
1.87
1.85
1.82
11
1.48
1.00
0.61
1.58
2.92
0.60
f1 (ppm)

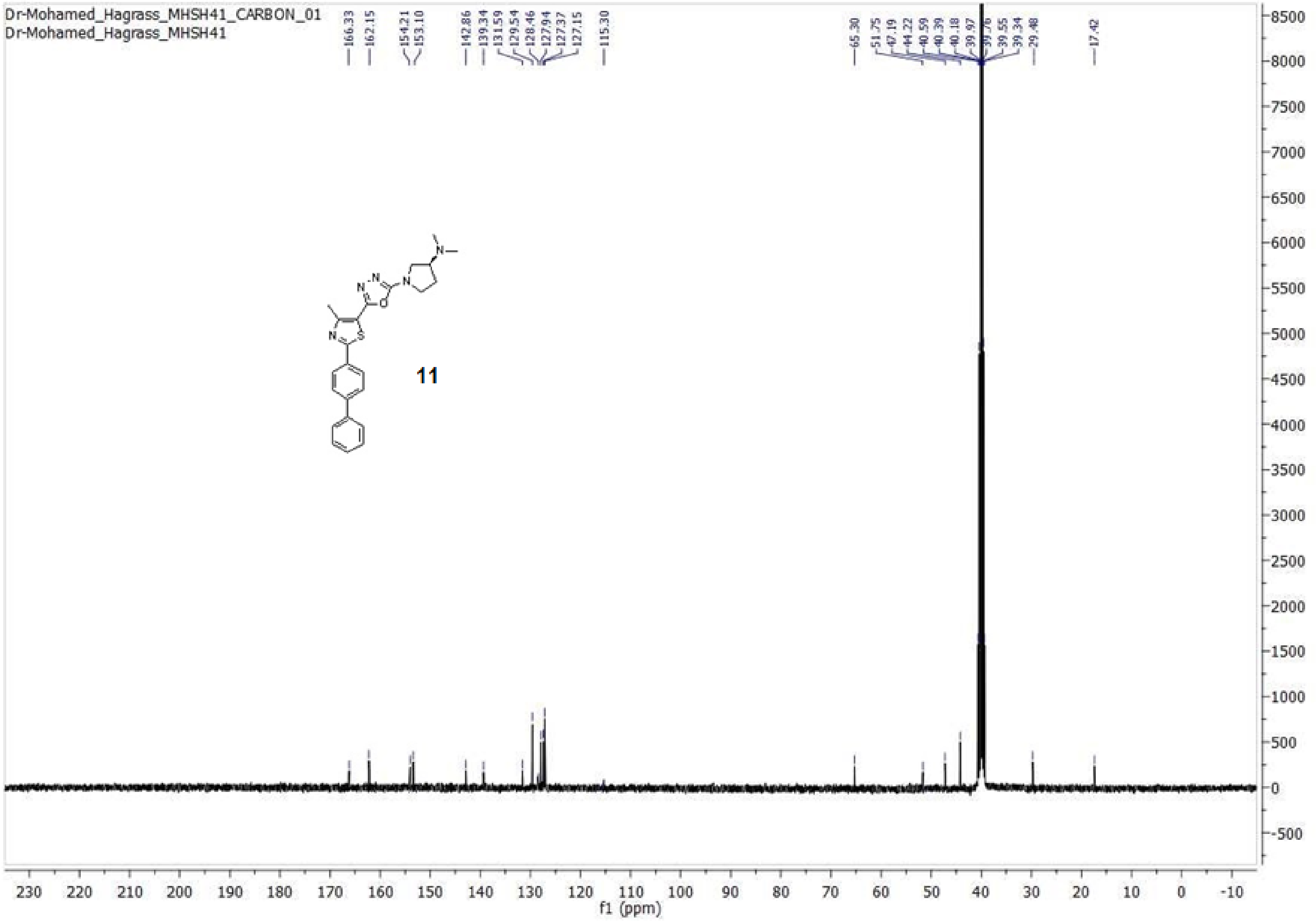
Dr-Mohamed_Hagrass_MHSH41_CARBON_01
Dr-Mohamed_Hagrass_MHSH41
11
f1 (ppm)

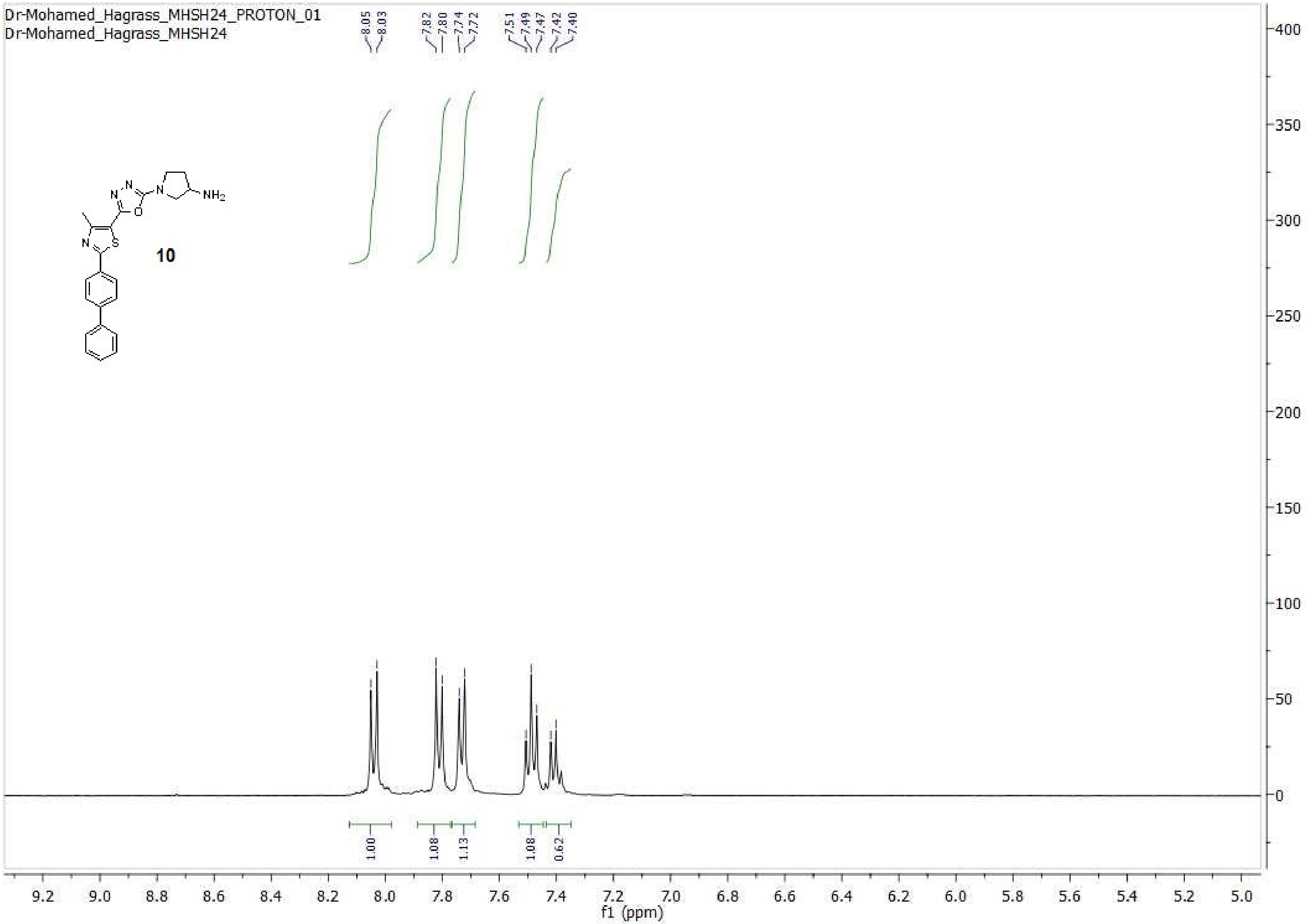
Dr-Mohamed_Hagrass_MHSH24_PROTON_01
Dr-Mohamed_Hagrass_MHSH24
8.05
8.03
7.82
7.80
7.74
7.72
7.51
7.49
7.47
7.42
7.40
10
1.00
1.08
1.13
1.08
0.62
f1 (ppm)

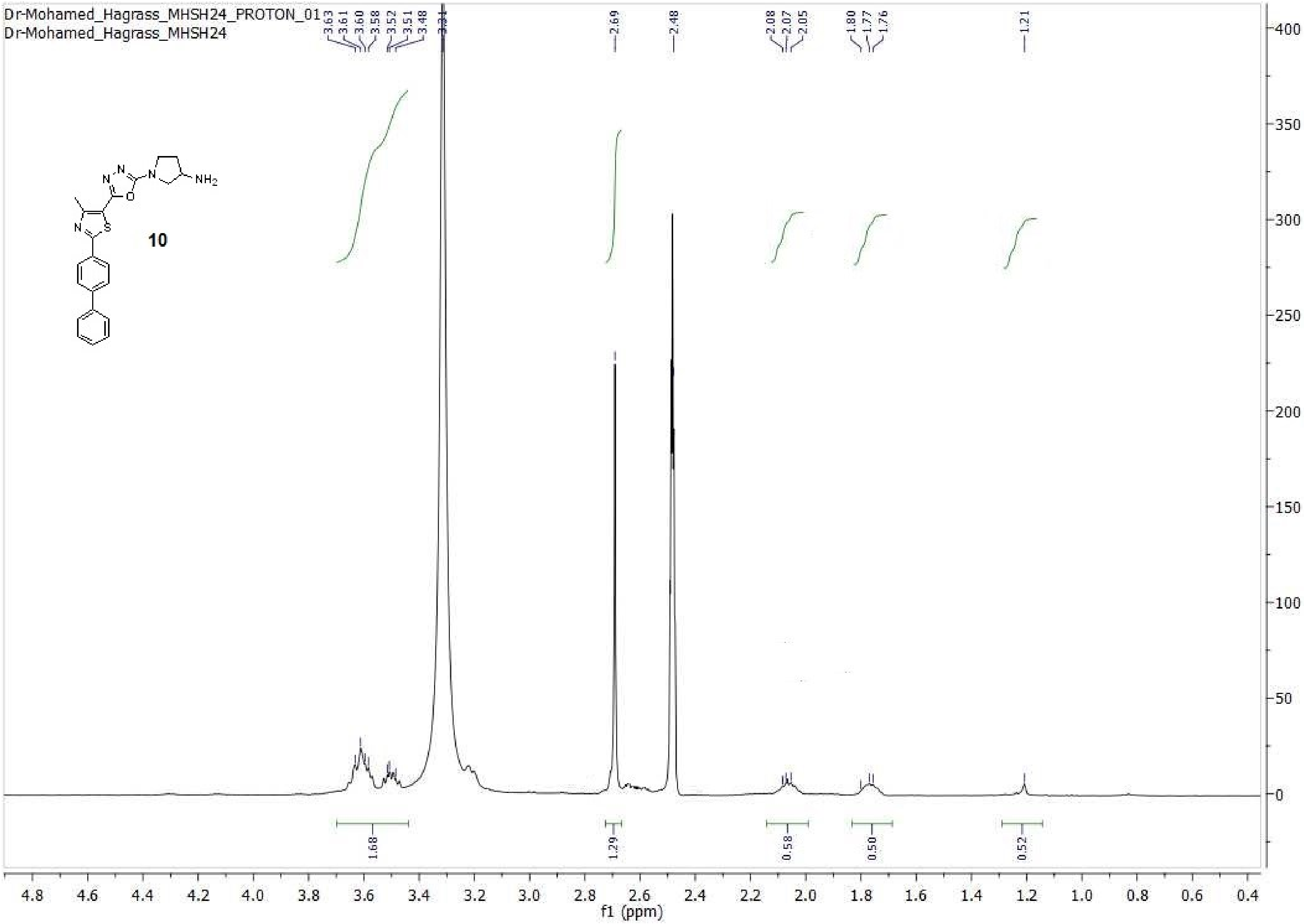
Dr-Mohamed_Hagrass_MHSH24_PROTON_01
Dr-Mohamed_Hagrass_MHSH24
3.63
3.61
3.60
3.58
3.52
3.51
3.48
2.69
2.48
2.08
2.07
2.05
1.80
1.77
1.76
1.21
NH2
N
O
S
10
1.68
1.29
0.58
0.50
0.52
f1 (ppm)

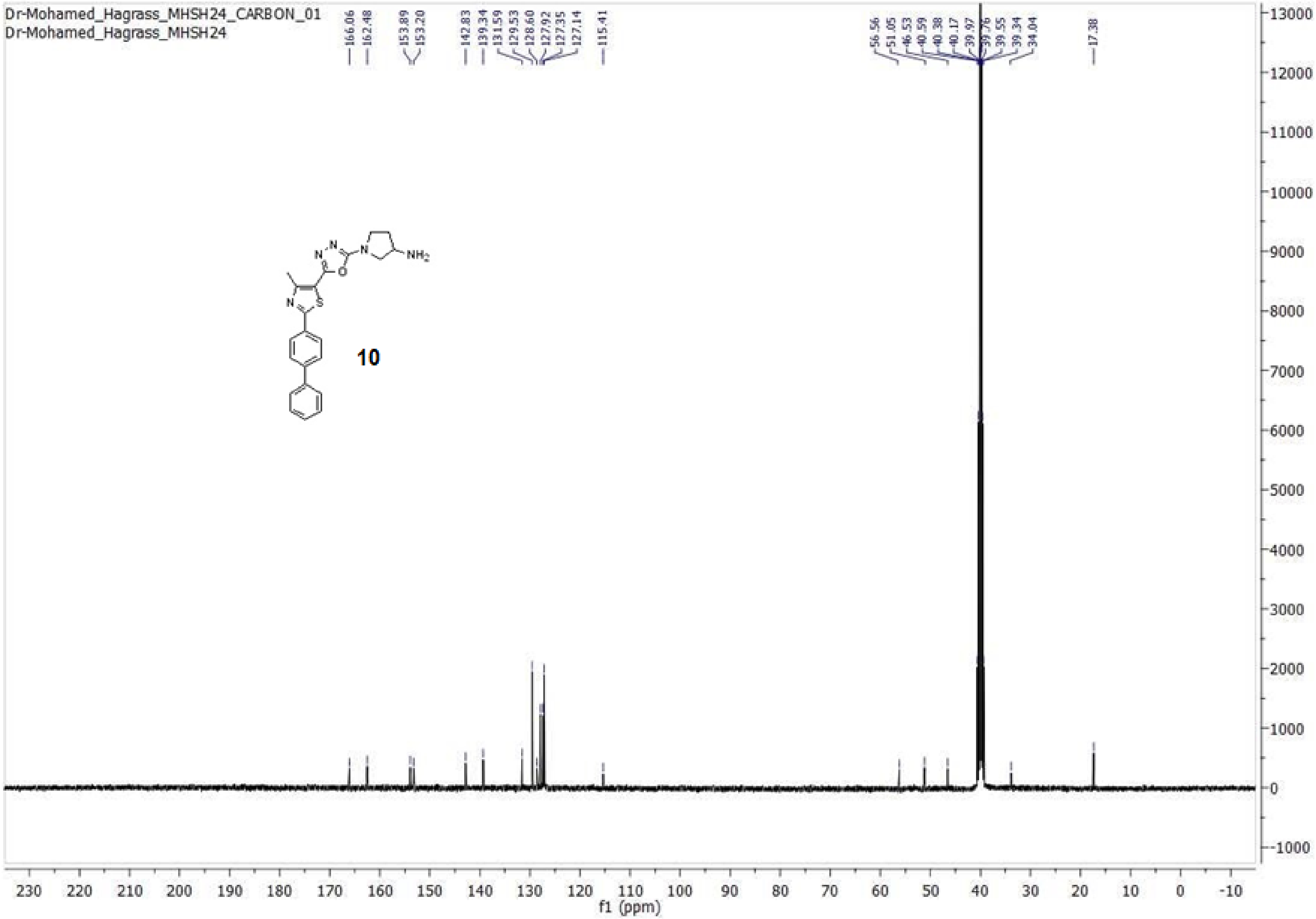

Dr-Mohamed_Hagrass_MHSH24_CARBON_01
Dr-Mohamed_Hagrass_MHSH24
166.06
162.48
153.89
153.20
142.83
139.34
131.59
129.53
128.60
127.92
127.35
127.14
115.41
56.56
51.05
46.53
40.59
40.38
40.17
39.97
39.76
39.55
39.34
34.04
17.38
10
f1 (ppm)

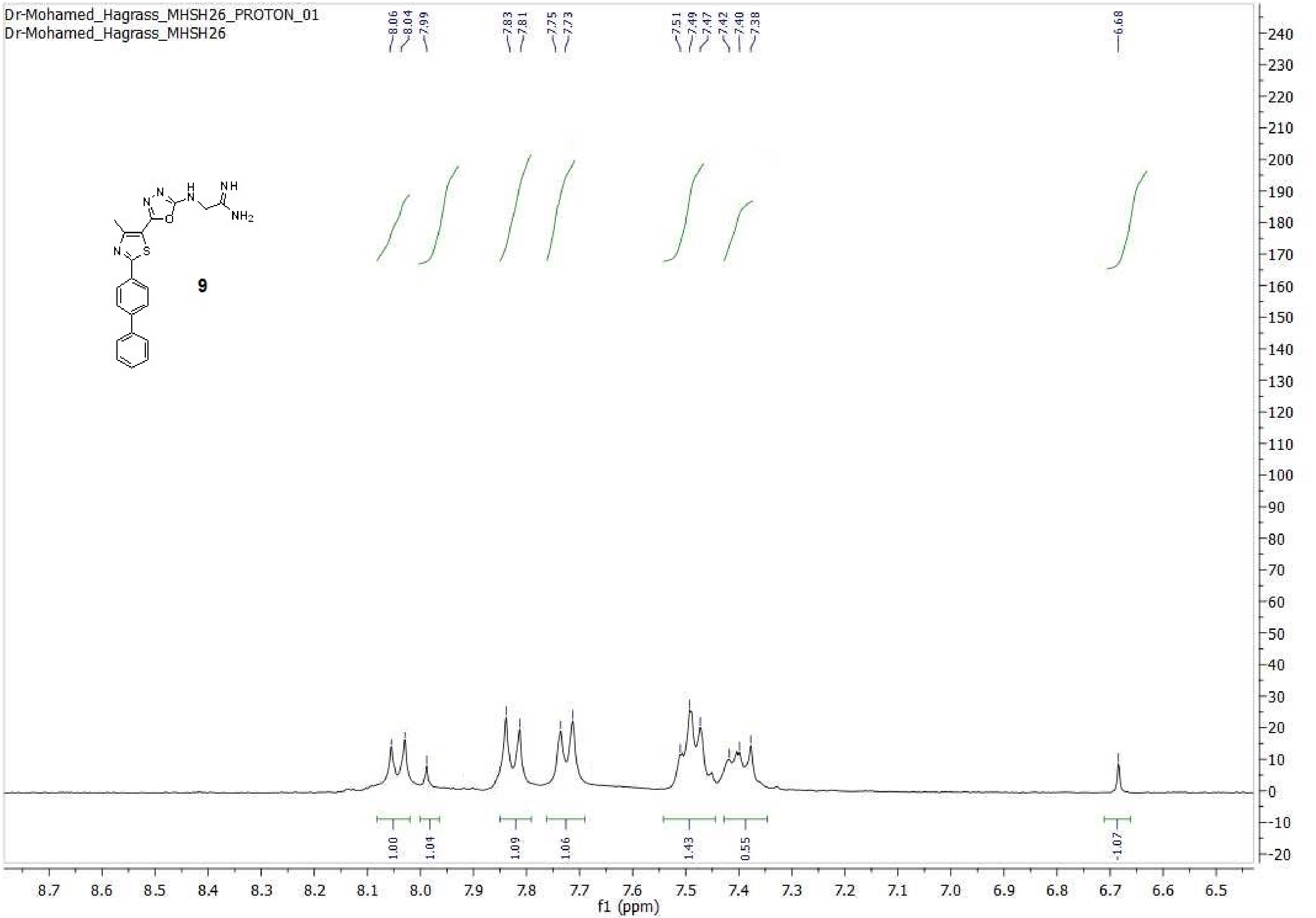
Dr-Mohamed_Hagrass_MHSH26_PROTON_01
Dr-Mohamed_Hagrass_MHSH26
8.06
8.04
7.99
7.83
7.81
7.75
7.73
7.51
7.49
7.47
7.42
7.40
7.38
6.68
1.00
1.04
1.09
1.06
1.43
0.55
-1.07
NH
NH2
9
f1 (ppm)

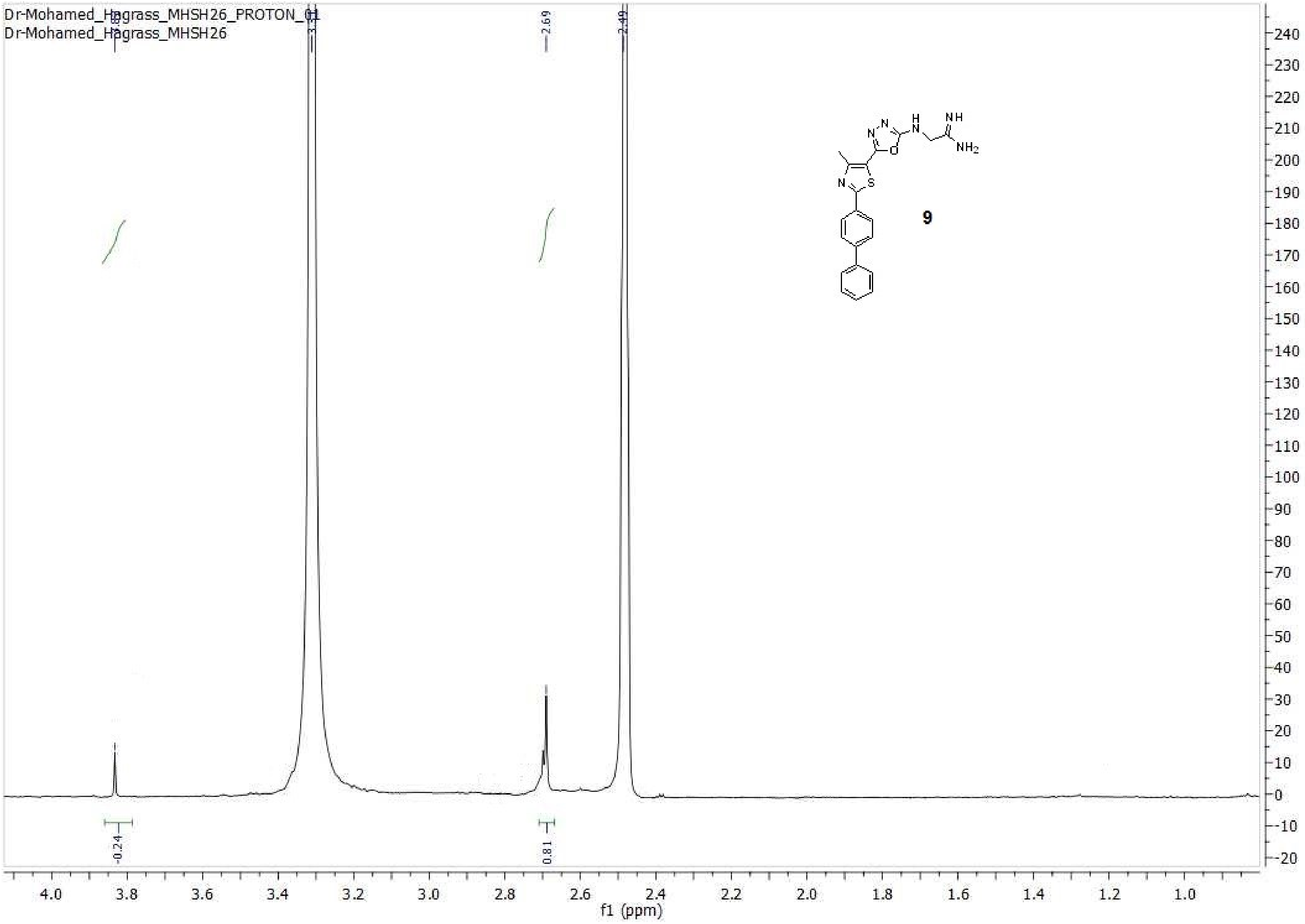

Dr-Mohamed_Hagrass_MHSH26_PROTON_01
Dr-Mohamed_Hagrass_MHSH26
2.69
2.49
9
-0.24
0.81
f1 (ppm)

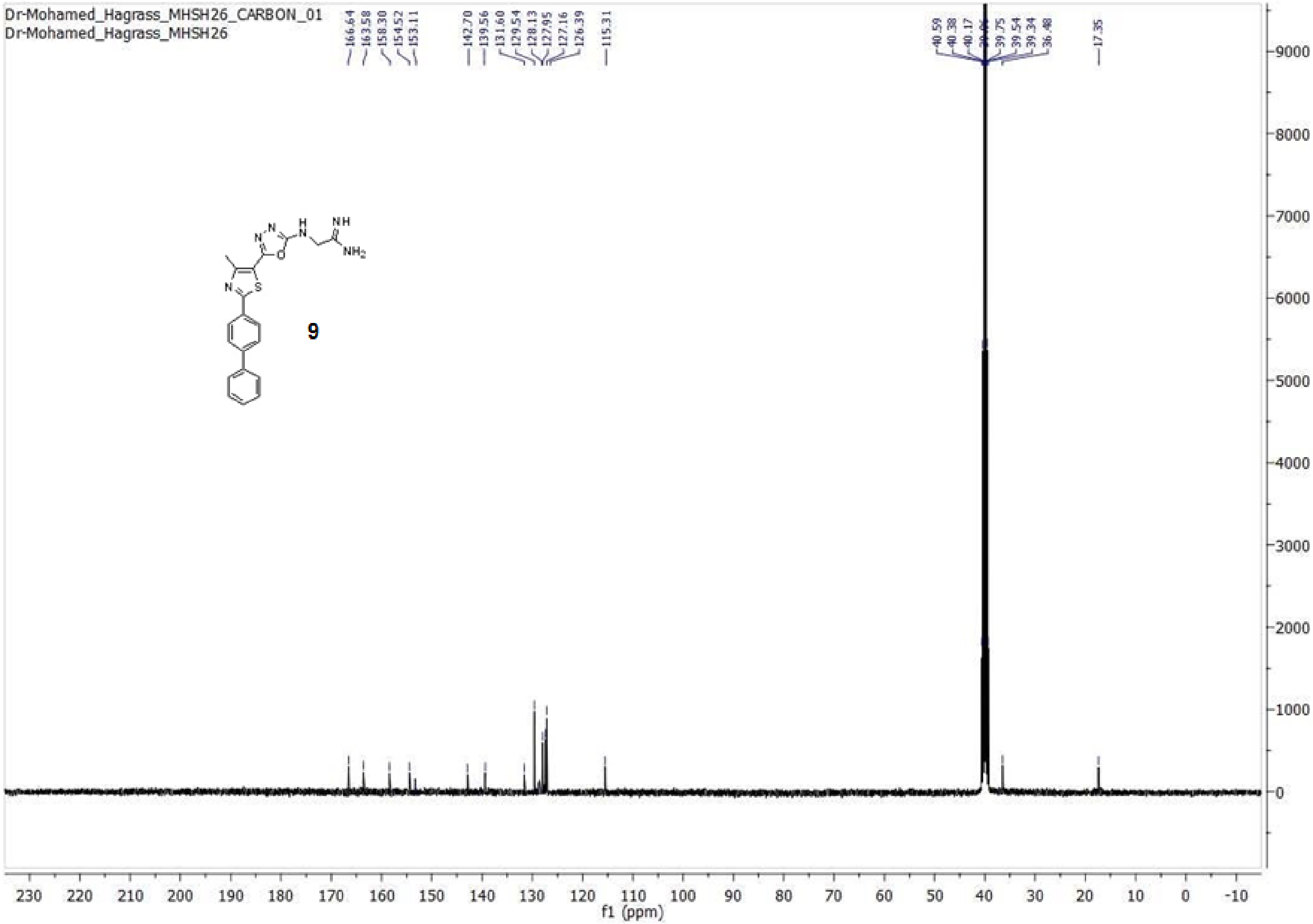
Dr-Mohamed_Hagrass_MHSH26_CARBON_01
Dr-Mohamed_Hagrass_MHSH26
166.64
163.58
158.30
154.52
153.11
142.70
139.56
131.60
129.54
128.13
127.95
127.16
126.39
115.31
40.59
40.38
40.17
39.75
39.54
39.34
36.48
17.35
9
f1 (ppm)

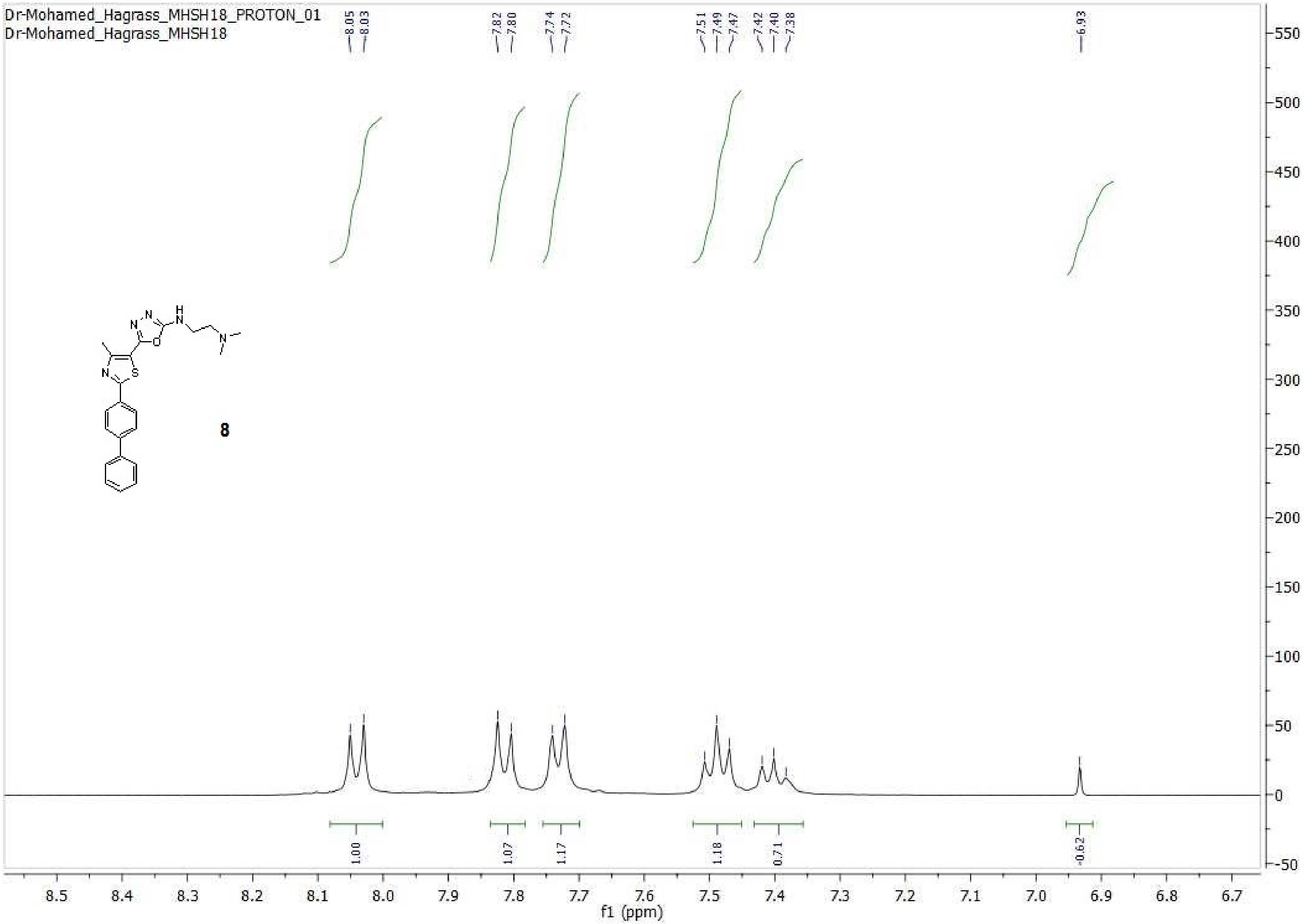
Dr-Mohamed_Hagrass_MHSH18_PROTON_01
Dr-Mohamed_Hagrass_MHSH18
8.05
8.03
7.82
7.80
7.74
7.72
7.51
7.49
7.47
7.42
7.40
7.38
6.93
8
1.00
1.07
1.17
1.18
0.71
0.62
f1 (ppm)

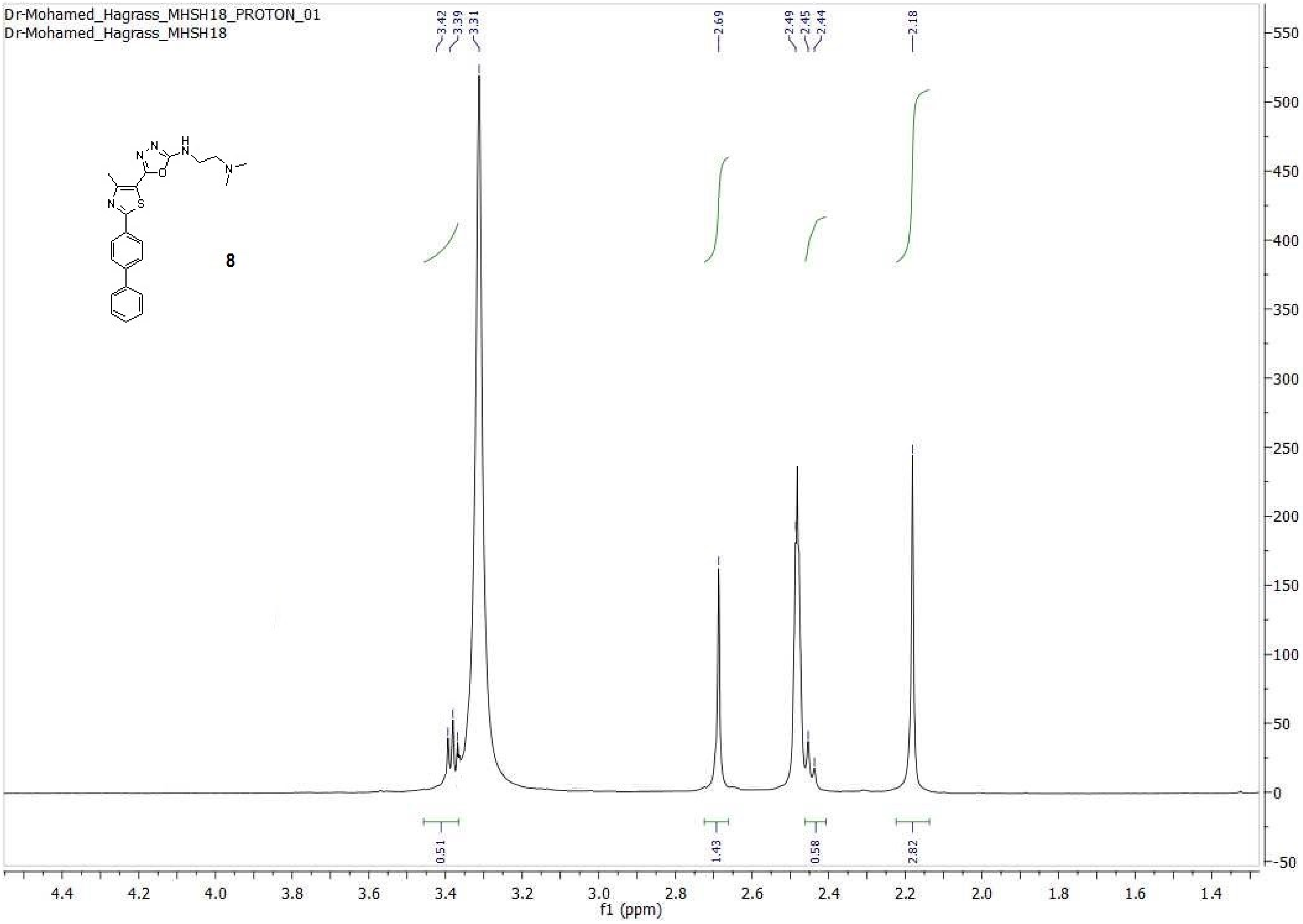
Dr-Mohamed_Hagrass_MHSH18_PROTON_01
Dr-Mohamed_Hagrass_MHSH18
3.42
3.39
3.31
2.69
2.49
2.45
2.44
2.18
8
0.51
1.43
0.58
2.82
f1 (ppm)

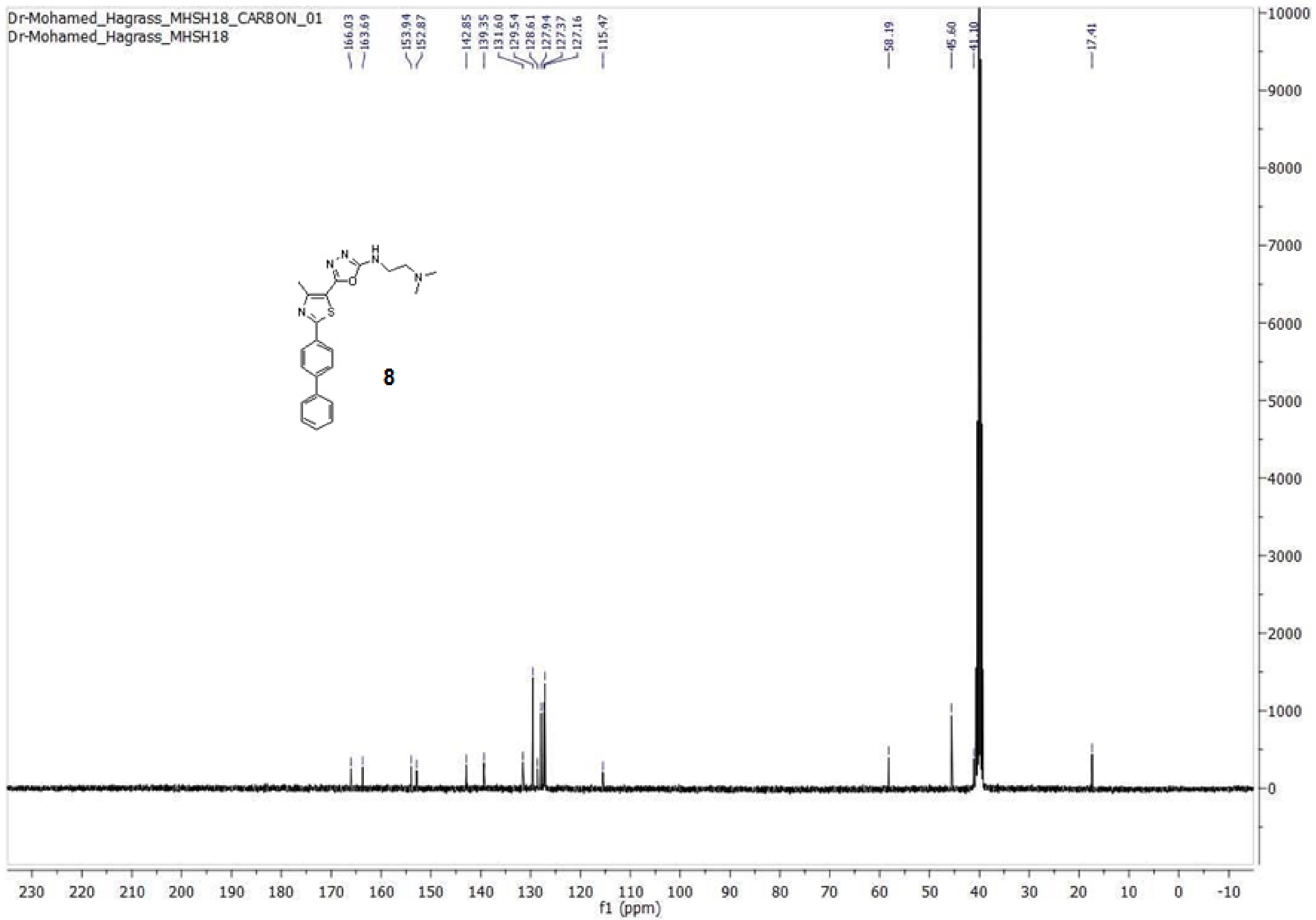
Dr-Mohamed_Hagrass_MHSH18_CARBON_01
Dr-Mohamed_Hagrass_MHSH18
166.03
163.69
153.94
152.87
142.85
139.35
131.60
129.54
128.61
127.94
127.37
127.16
115.47
58.19
45.60
41.10
17.41
8
f1 (ppm)

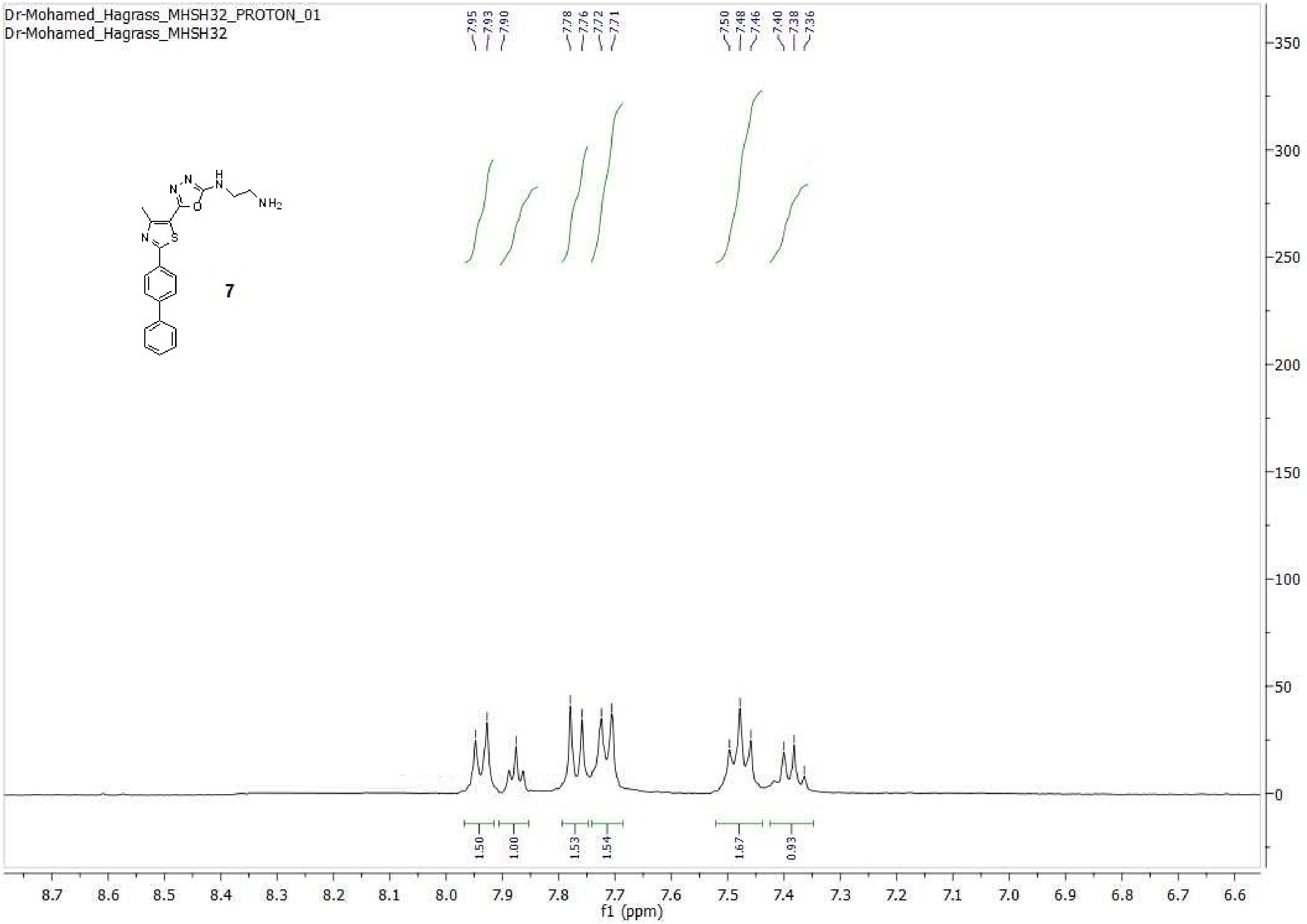
Dr-Mohamed_Hagrass_MHSH32_PROTON_01
Dr-Mohamed_Hagrass_MHSH32
7.95 7.93 7.90
7.78 7.76 7.72 7.71
7.50 7.48 7.46 7.40 7.38 7.36
7
1.50 1.00 1.53 1.54 1.67 0.93
8.7 8.6 8.5 8.4 8.3 8.2 8.1 8.0 7.9 7.8 7.7 7.6 7.5 7.4 7.3 7.2 7.1 7.0 6.9 6.8 6.7 6.6
f1 (ppm)
350 300 250 200 150 100 50 0

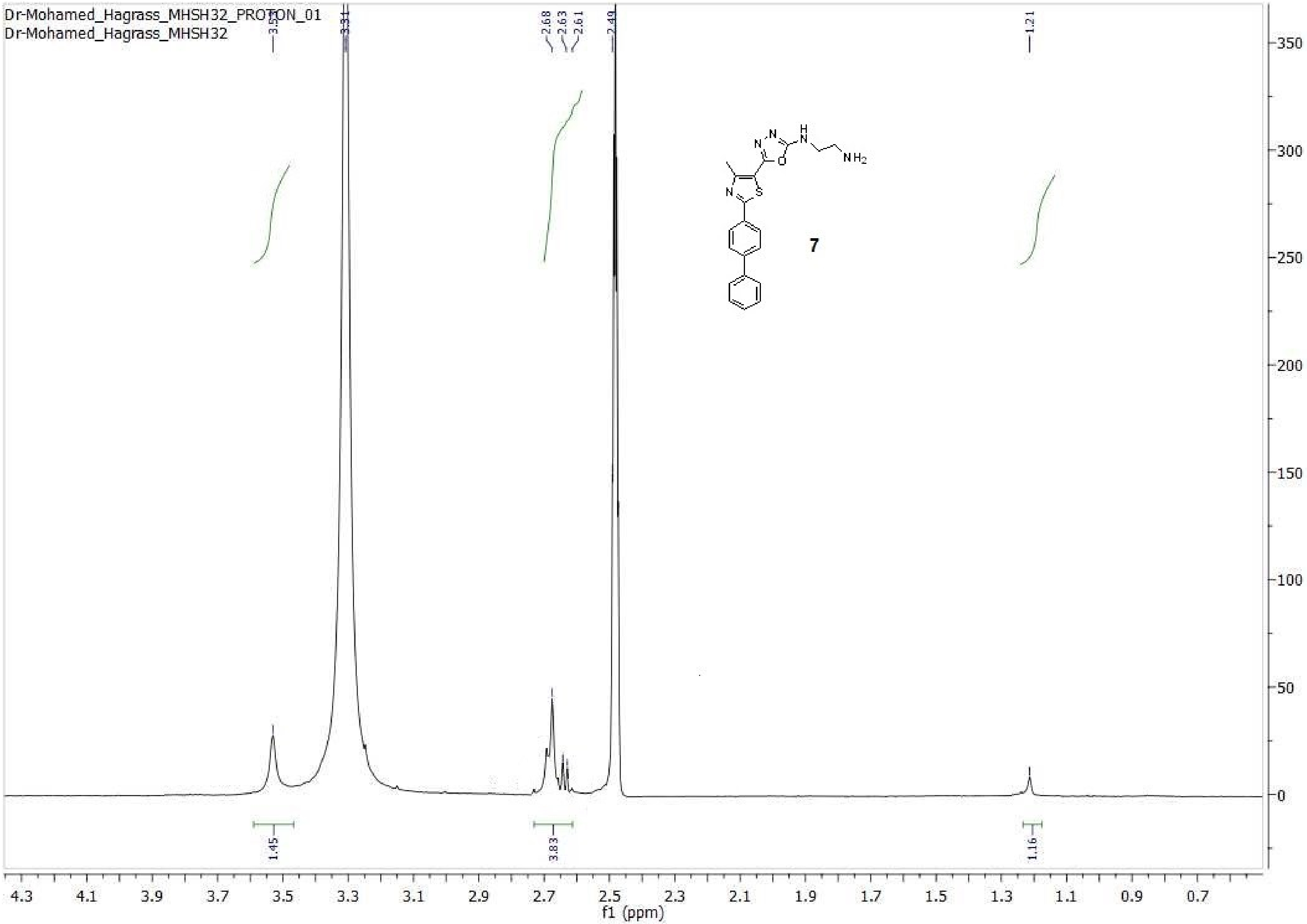
Dr-Mohamed_Hagrass_MHSH32_PROTON_01
Dr-Mohamed_Hagrass_MHSH32
3.53
3.31
2.68
2.63
2.61
2.49
1.21
7
1.45
3.83
1.16
f1 (ppm)

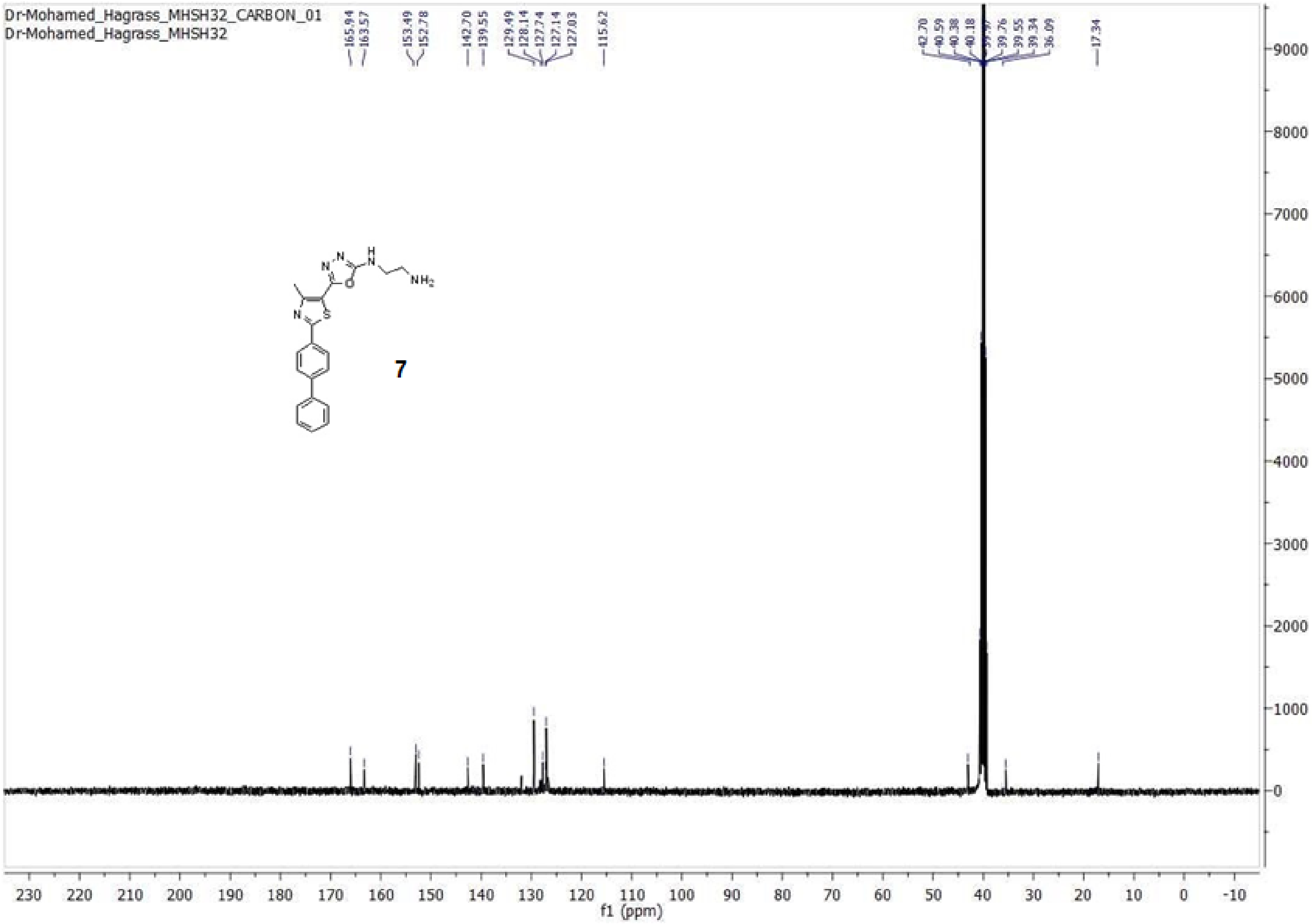
Dr-Mohamed_Hagrass_MHSH32_CARBON_01
Dr-Mohamed_Hagrass_MHSH32
165.94
163.57
153.49
152.78
142.70
139.55
129.49
128.14
127.74
127.14
127.03
115.62
42.70
40.59
40.38
40.18
39.76
39.55
39.34
36.09
17.34
7
f1 (ppm)
