# Supplementary figures and images for: Evaluation of bisphenylthiazoles as a promising class for combating multidrug-resistant fungal infections
Source: PLoS One. 2021 Nov 4;16(11):e0258465. doi: 10.1371/journal.pone.0258465 (PMC8568133; doi:10.1371/journal.pone.0258465)

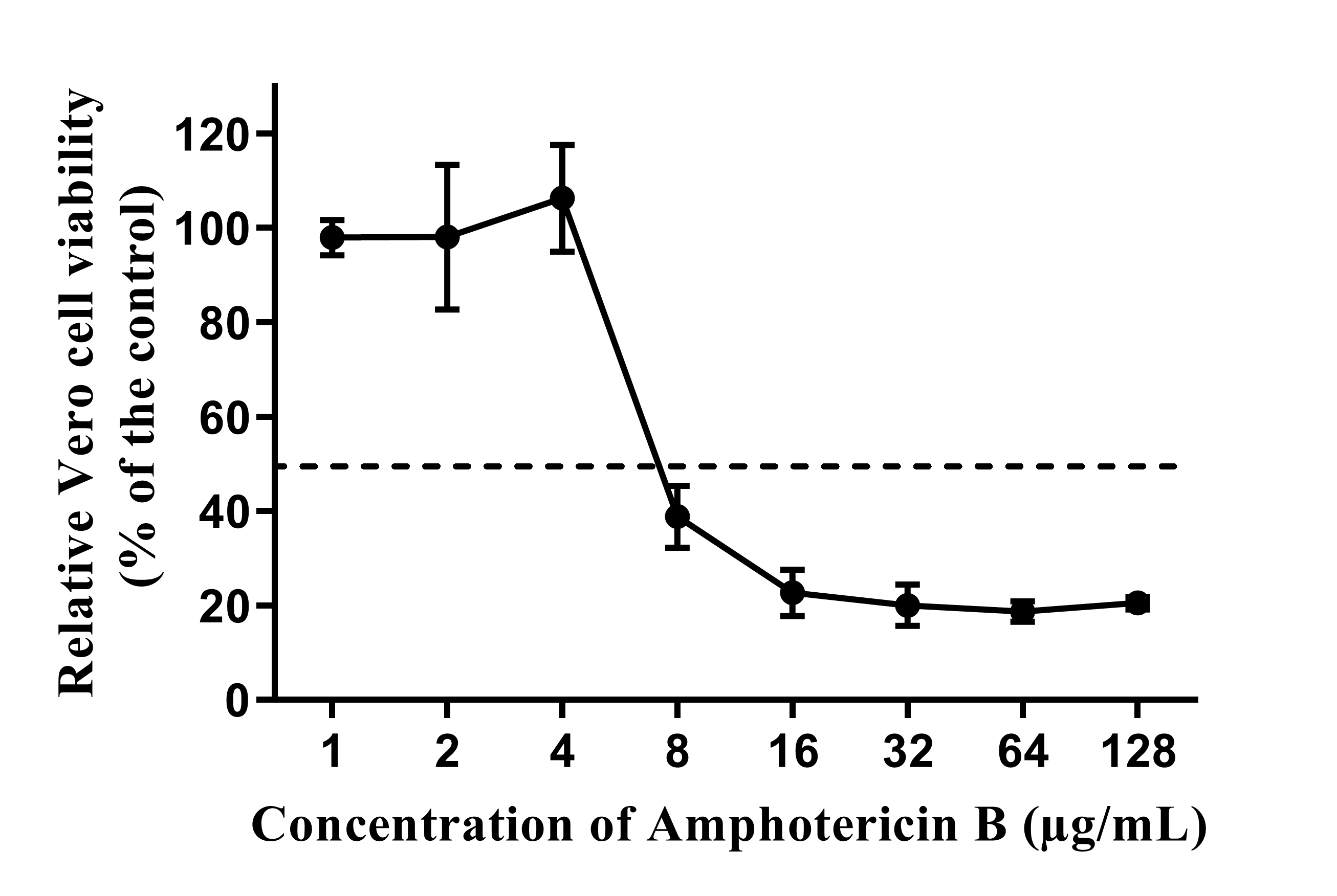

Supplement: S1 Fig — (TIF) [file pone.0258465.s002.tif]
